# Conformal inference for reliable single cell RNA-seq annotation

**DOI:** 10.1093/bioinformatics/btaf521

**Published:** 2025-09-18

**Authors:** Marcos López-De-Castro, Alberto García-Galindo, José González-Gomariz, Rubén Armañanzas

**Affiliations:** Institute of Data Science and Artificial Intelligence (DATAI), University of Navarra, Pamplona, Navarra 31009, Spain; TECNUN School of Engineering, University of Navarra, Donostia-San Sebastián, Basque Country, 20018, Spain; Cancer Center CCUN, Clínica Universidad de Navarra, Pamplona, Navarra, 31080, Spain; Institute of Data Science and Artificial Intelligence (DATAI), University of Navarra, Pamplona, Navarra 31009, Spain; TECNUN School of Engineering, University of Navarra, Donostia-San Sebastián, Basque Country, 20018, Spain; Cancer Center CCUN, Clínica Universidad de Navarra, Pamplona, Navarra, 31080, Spain; Institute of Data Science and Artificial Intelligence (DATAI), University of Navarra, Pamplona, Navarra 31009, Spain; TECNUN School of Engineering, University of Navarra, Donostia-San Sebastián, Basque Country, 20018, Spain; Cancer Center CCUN, Clínica Universidad de Navarra, Pamplona, Navarra, 31080, Spain; Institute of Data Science and Artificial Intelligence (DATAI), University of Navarra, Pamplona, Navarra 31009, Spain; TECNUN School of Engineering, University of Navarra, Donostia-San Sebastián, Basque Country, 20018, Spain; Cancer Center CCUN, Clínica Universidad de Navarra, Pamplona, Navarra, 31080, Spain

## Abstract

**Motivation:**

Despite the inherent complexity associated to automatic cell type assignments, most supervised learning models overlook rigorous uncertainty quantification on the annotations. Although some existing pipelines incorporate rejection options under predefined circumstances, they usually rely on arbitrary assumptions and do not provide statistical guarantees. In this work, we propose a methodology based on the conformal prediction framework to provide reliable single-cell annotations. Conformal prediction provides statistical guarantees on the outcome predictions without making any assumption about the underlying distribution of the data. Our methodological proposal leverages conformal inference to address two critical challenges in single-cell RNA sequencing annotations: (i) detect out-of-distribution cell types in the query data; and, (ii) perform reliable uncertainty quantification of the cell annotations through well-calibrated prediction sets.

**Results:**

We evaluated the anomaly detector and the uncertainty-aware annotator in 10 batched experiments derived from various tissues. Specifically, we studied three different annotation taxonomies (standard, classwise, and cluster) alongside three different non-conformity measures. The results showed that our anomaly detector effectively identified previously unseen cell types, producing well-calibrated prediction sets. This rigorous annotation helped maintain coverage probabilities at the expected significance level. Finally, we illustrate how the integration of conformal prediction outputs enhanced further downstream analyses.

**Availability and implementation:**

The automatic scRNA-seq annotator is available at https://github.com/digital-medicine-research-group-UNAV/conformalized_single_cell_annotator and https://doi.org/10.5281/zenodo.15870599.

## 1 Introduction

Single-cell RNA sequencing (scRNA-seq) enables the profiling of human tissues at individual cell resolution, allowing the study of complex cell population dynamics along different processes (Zhu *et al.* 2020, Jovic *et al.* 2022). Exploring transcriptomic patterns with this unprecedented resolution has become an essential tool for understanding cellular diversity, as well as for assessing the role of specific cell populations in disease progression and therapeutic response.

A key step in the analysis of scRNA-seq data often involves the accurate categorization of cells into cell types (Heumos *et al.* 2023). The identification of cell types from similar transcriptomic signatures has been mainly addressed through two different data-driven approaches. The first approach employs unsupervised clustering methods to group cells with similar gene-expression patterns. These clusters are then manually annotated by analysing relevant biomarkers with the help of expert-domain knowledge (Aran *et al.* 2019). This approach, however, presents several drawbacks, such as the labor-intensive nature of the annotation process and the lack of reproducibility (Kiselev *et al.* 2019). To overcome these limitations, a second approach based on supervised classification algorithms was recently introduced as a promising alternative to automatic annotation of cell types (Abdelaal *et al.* 2019, Pliner *et al.* 2019, Clarke *et al.* 2021). These annotators usually rely on learning transcriptomic patterns from well-annotated reference datasets and exploit them to classify new cells from query datasets. Most of the existing automatic annotators are built on top of conventional machine learning methods, which presents a common limitation: they lack reliable uncertainty estimates of the cell type annotation. This becomes especially problematic in single-cell classification, where uncertainty can originate from multiple sources, including biological variability and the presence of batch effects (Butler *et al.* 2018).

In this study, we propose a method for reliable single-cell annotation with integrated uncertainty quantification. In particular, we present a novel procedure based on conformal prediction, a robust framework designed to endow machine learning models with statistical guarantees by means of rigorous prediction sets, without making any assumption about the underlying probability distribution (Toccaceli 2022, Vovk *et al.* 2022, Angelopoulos and Bates 2023). For each new query cell, a set of plausible labels covering the true type with a confidence level is returned. The size of the prediction sets generated by the conformal predictor can be directly interpreted as uncertainty estimates. A large prediction set indicates an ambiguous profile, less confidence in the prediction, and, therefore, should be treated with caution. Conversely, a narrow prediction set reflects high confidence in the cell type assignment, supported by well-characterized gene-expression signatures.

Since our approach is built on conformal prediction, the statistical guarantees hold as long as the independent and identically distributed (i.i.d.) assumption is satisfied. However, scRNA-seq experiments often involve the analysis of different tissues in which novel cell populations may not have been available during the training phase. In such cases, the i.i.d. assumption is violated, potentially leading to untrustworthy prediction sets failing to retrieve the desired confidence. These cells should remain unassigned since their cell type has not been considered during the training step (Theunissen *et al.* 2025). Existing annotators have usually performed the identification of novel populations by analysing the classifier’s posterior scores and defining threshold-based rejection strategies (Goyal *et al.* 2022, Theunissen *et al.* 2024). Unfortunately, it is well-known that most of the classification algorithms, including neural networks, tend to produce miscalibrated scores that do not reflect true probabilities (Boström 2008, Minderer *et al.* 2021). In contrast, we have coupled our annotator with an unsupervised anomaly detector to exclude those cell types that were not present in the reference data from the annotation process. This anomaly detector combines the power of black-box unsupervised one-class models with the explainability of the hypothesis testing theory (i.e. decision accordingly with a valid null distribution). This is achieved using the conformal prediction framework again, which derives valid *P*-values for the hypothesis tests.

Although conformal prediction has been applied in many fields, including spatial transcriptomics data (Sun *et al.* 2024), to the best of our knowledge only one work has previously discussed the benefits of using conformal prediction for single-cell label annotation (Khatri and Bonn 2022). In their work, the application is limited to the standard conformal taxonomy, whereas the problem of detecting unknown cell types is not addressed. Alternatively, we here provide a practical and statistically principled methodology for scRNA‐seq annotation that leverage conformal prediction to (i) detect out-of-distribution (OOD) cells from the ontology reference using a fully non-parametric approach; and, (ii) annotate scRNA‐seq data with statistical guarantees through prediction sets. Our approach was validated across diverse reference and query datasets, and it is suitable for use with any well-established single-cell ontology.

## 2 Materials and methods

### 2.1 Notation and problem setup

Formally, let us assume a labeled reference dataset $D_{\text{ref}}=(x_{1},y_{1}),\ldots ,(x_{n},y_{n})$, where $x_{i}$ encodes the transcriptomic expression profiles, and $y_{i}$ denotes the cell type. We assume that $D_{\text{ref}}$ is i.i.d. sampled from an unknown distribution $P_{XY}$, where $X\in R^{p}$ is the feature space, and $Y=\{c_{1},\ldots ,c_{K}\}$ is a discrete label space. Additionally, let us consider a query dataset $D_{\text{test}}=(x_{1},y_{1}),\ldots ,(x_{m},y_{m})$, where $y_{i}$ denotes the type label for each cell, which is initially unknown and to be inferred. Without loss of generality, we assume that $D_{\text{ref}}$ and $D_{\text{test}}$ share a common feature space X (i.e. the sequenced genes are the same in both datasets).

We face the task of reliable supervised cell-type annotation through the lens of predictive uncertainty quantification. The goal is to construct a set-valued predictor $C:X\to 2^{Y}$ that, for a new sequenced cell $x_{n+1}\in D_{\text{test}}$, produce a prediction set $C(x_{n+1})\subseteq Y$ of plausible types with the following coverage guarantee:


(1) $$P(y_{n+1}\in C(x_{n+1}))\geq 1-\alpha ,$$

where $y_{n+1}$ is the ground truth, and α∈[0,1] is a nominal error level. The construction of C is approached through the lens of conformal prediction (Vovk *et al.* 2022), which provides formal coverage guarantees as long as the i.i.d. assumption holds.

However, a common scenario in single-cell annotation is the presence of novel cell populations in $D_{\text{test}}$ that may have not been present on the reference $D_{\text{ref}}$ (i.e. $y_{n+1}\notin Y$). This situation violates the i.i.d. assumption, breaking the conformal guarantee. Hence, we also consider the problem of anomalous cell type detection and formulate it as a statistical hypothesis test (Laxhammar and Falkman 2010, Bates *et al.* 2023):


(2) $$H_{0}:x_{n+1}∼P_{XY}.$$

The construction of valid *p*-values for testing (2) is our second goal. Thus, we propose a sequential machine learning pipeline comprising two modules: a novel cell type OOD detector, followed by a conformal classifier. Our proposal integrates recent advances from conformal inference to provide distinct types of statistical guarantees. Specifically, we employ an inductive scheme (Papadopoulos *et al.* 2002), such that $D_{\text{ref}}$ is partitioned into a proper training set $D_{\text{train}}$ and a calibration set $D_{\text{cal}}$. Under this approach, $D_{\text{train}}$ is used to train the underlying models, whereas $D_{\text{cal}}$ is then employed to calibrate them and provide a reliable prediction (see Fig. S1, available as supplementary data at *Bioinformatics* online). Throughout this work, we adopt the following convention: OOD observations refer to cells that are OOD as defined previously. In-distribution (ID) observations refer to cells whose cell types are present in the reference data but may not necessarily satisfy the exchangeability assumption. Lastly, i.i.d. observations refer to cells meeting the exchangeability condition.

### 2.2 Reliable unsupervised identification of novel cell types

#### 2.2.1 Underlying OOD detector

We propose an autoencoder as the underlying novel cell type OOD detector: X→R that, for each new sample, produces an anomaly score. Intuitively, larger scores indicate higher evidence that a sample may be an outlier. The autoencoder comprises two feed-forward multi-layer neural networks: the encoder and the decoder. The encoder maps the original input data $x_{i}\in R^{p}$ to a low-dimensional representation $z_{i}\in R^{q}$, with q<p. The decoder reconstructs the low-dimensional representation back again to the original space, yielding $x_{i}^{\prime }\in R^{p}$. This architecture is trained in an unsupervised way on $D_{\text{train}}$, clean of any outliers, to minimize the mean squared reconstruction error using an Adam optimizer. Additional details about the implementation are given in Section S3.1, available as supplementary data at *Bioinformatics* online.

#### 2.2.2 Induction of conformal valid *p*-values

The procedure to determine valid *p*-values for testing (2) is a statistical wrapper around the underlying autoencoder. Once trained, we computed the anomaly scores on the calibration data $s_{i}=\text{OOD}(x_{i}),x_{i}\in D_{\text{cal}}$. In the realm of conformal inference, these values are referred to as the non-conformity scores. Since the calibration samples are i.i.d., the ranking of the non-conformity scores is uniformly distributed. In this way, if we compute a conformal *p*-value for a new sample as


(3) $$p_{n+1}=\frac{1+\sum_{i=1}^{n}1(s_{i}\geq s_{n+1})}{n_{\text{cal}}},$$

where 1(·) is the indicator function and $s_{n+1}$ is the non-conformity score for the new sample, this *p*-value is *marginally valid* for testing (2). In particular,


(4) $$P(p_{n+1}\leq \alpha_{o}\;)\leq \alpha_{o},\;\;\text{for}\;\text{any}\;\alpha_{o}\in [0,1]$$

is satisfied, allowing to evaluate whether each new sample from $D_{\text{test}}$ is a novel cell type in a more statistical informed way than traditional one-class models (Pimentel *et al.* 2014).

A powerful advantage of the conformal approach is that the derived conformal *p*-values directly control the marginal False Positive Rate (FPR). For any chosen significance level $\alpha_{o}$, the expected FPR is bounded by $\alpha_{o}$ over all possible calibration sets $D_{\text{cal}}$:


(5) $$E[\mathrm{FPR}(\alpha_{o},D_{\text{cal}})]\leq \alpha_{o}.$$

This means that, if we set a decision threshold $\alpha_{o}$, the long-run proportion of ID cells that are falsely flagged as OOD is guaranteed to not exceed $\alpha_{o}$, provided the exchangeability condition between the reference and query data holds. Additional guarantees can be introduced through the calibration-conditional conformal *p*-values (Bates *et al.* 2023), at expenses of losing statistical power. Calibration-conditional conformal *p*-values guarantees that, for our selected $D_{\text{cal}}$, FPR is never above $\alpha_{o}$ with some probability 1−δ (Bates *et al.* 2023).

### 2.3 Uncertainty-aware cell type annotation

We perform experiments with three different underlying multi-class annotators; Scmap, CellTypist, and a feed-forward deep neural network which we will refer to as ‘TorchNet’. We impose the following condition to the underlying annotator: for a new cell, a softmax score for each of the *K* types must be returned $\hat{f}:X\to \Delta^{k}$. This allows us to use well-established non-conformity functions.

#### 2.3.1 Conformal calibration

As with the anomaly detector calibration, the non-conformity function is a key element in the development of a conformal set-valued predictor. The choice of a suitable non-conformity function is a critical step to induce effective prediction sets. We have implemented three state-of-the-art non-conformity functions derived from the outputted softmax scores of the underlying classifier:

-
THR (Sadinle *et al.* 2019): The threshold-based non-conformity score at an input *x* and a label *y* is defined as
  (6) $$s_{\mathrm{THR}}(x,y)=1-{\hat{f}}_{y}(x)$$

where ${\hat{f}}_{y}(x)$ is the softmax score for the true class.

-
APS (Romano *et al.* 2020): Unlike the THR function, the adaptive prediction sets employs the softmax scores of all classes. Specifically, it computes the cumulative sum of the sorted scores as the non-conformity score:
  (7) $$\begin{array}{c} s_{\mathrm{APS}}(x,y)=\sum_{y^{\prime }\in Y}{\hat{f}}_{y^{\prime }}(x)1(r_{f}(x,y^{\prime })<r_{f}(x,y))+u\cdot {\hat{f}}_{y^{\prime }}(x) \end{array}$$

where $r_{f}$ is the rank of the softmax scores, and *u* is a random variable derived from a uniform distribution between [0, 1].

-
RAPS (Angelopoulos *et al.* 2021): The regularized adaptive prediction sets function adds a regularization term to the APS to penalize noisy scores and reduce the prediction set sizes:
  (8) $$s_{\mathrm{RAPS}}(x,y)=s_{\mathrm{APS}}(x,y)+\eta {\left(r_{f}(x,y)-\kappa \right)}^{+},$$

where η represents the weight of the regularization, κ is a regularization parameter, and ${\left(\right)}^{+}$ refers to the positive part.

For each function, non-conformity scores are computed on $D_{\text{cal}}$. Then, instead of computing a single *p*-value for a new query sample, we calculate a set of *p*-values as in (4), one for each of the possible types a cell may belong to. In that way, each *p*-value represents how plausible each cell type is for a specific sample. For a given α∈[0,1], we compute a prediction set given by all the types whose *p*-value is greater than α, formally:


(9) $$C(x_{n+1})=\{c_{k}\in Y|p_{k}\geq \alpha \}$$

These prediction sets satisfy marginal coverage guarantees (1) under the i.i.d. assumption. Note that the guarantees are exclusively marginal, on average, across the entire population. We also consider Mondrian conformal prediction (Vovk *et al.* 2022), a modification of the original procedure that satisfy validity conditioned on each of the categories defined by a chosen taxonomy. One of the most powerful taxonomies relies on mapping each sample to the label space κ:X×Y→Y. This taxonomy is known as classwise conformal predictors, and the resulting set-valued predictor achieves class-conditional coverage guarantees:


(10) $$P(y_{n+1}\in C(x_{n+1})\;|\;y_{n+1}=c)\geq 1-\alpha ,\;\;\forall c\in Y$$

The coverage is here satisfied for every cell type in the reference dataset. This taxonomy is particularly useful for imbalanced datasets, as it guarantees long-term control of the error rate across all cell types. However, many single-cell reference data exhibit severe class imbalance, where a minority of cell types have a few dozens of observations and major cell types have thousands. In such highly imbalanced scenarios, classwise conformal predictors can become unstable for the rare classes. Introducing a cluster taxonomy within Mondrian conformal predictors can help in these situations (Ding *et al.* 2023), because it offers a balance between standard and classwise approaches, thereby improving the efficiency of the set predictions. Formally, this approach aims to satisfy,


(11) $$\begin{array}{c} P(y_{n+1}\in C(x_{n+1})\;|\;\hat{h}(y_{n+1})=m)\geq 1-\alpha ,\\ \;\;\forall m\in \{1,\ldots ,m\}, \end{array}$$

where $\hat{h}$ is a clustering function that maps each class of Y to one of the possible *M* clusters. The three described taxonomies, i.e. standard, classwise, and cluster, are included in our annotator.

### 2.4 Datasets and preprocessing

We assessed the performance of our approach with several publicly available datasets. Three were gathered from different human tissues, including lung, pancreas, and breast, and one from mouse tissue at gastrulation period.

For the lung-tissue analysis, we used a cross-tissue immune-cell atlas from healthy adult donors as a reference (Domínguez Conde *et al.* 2022). Immune transcriptional profiles are highly state-dependent and represents a challenge in single-cell annotation. To test our approach, we selected two independent query datasets. The first query consisted of single-cell RNA sequencing data from healthy lung tissue (GEO: GSE178360—accessions GSM5388411, GSM5388412, and GSM5388413) (Kadur *et al.* 2022). The second query comprised data from lung cancer patients (accessions GSE127465) (Zilionis *et al.* 2019), where we expect alterations in immune-cell transcriptomic profiles. As the nomenclature in the query datasets differed from the reference, we harmonized the cell population labels to enable direct comparison (see Section S4, available as supplementary data at *Bioinformatics* online).

For the pancreas tissue, we used the GSE84133 study (Baron *et al.* 2016) to create a batched experiment. From the four different subjects in the study, we manually built four reference datasets with three subjects each time, leaving the corresponding fourth subject as the query dataset. This splitting introduces biological variability between reference and query data when samples are gathered with the same technology and conditions, but on different individuals. For the breast tissue, we considered the GSE180878 study, covering a human breast atlas. The reference dataset was built from 12 assayed samples with prophylactic mastectomies, whereas the remaining four samples with reductive mammoplasties were set as query. Finally, the gastrulation dataset was collected from mouse embryos at nine sequential time points (Pijuan-Sala *et al.* 2019). We divided the data into two sequencing experimental batches, mimicking reference and query datasets.

In total, 10 different experiments were run (see Tables S1–S8, available as supplementary data at *Bioinformatics* online, for additional details). We preprocessed each experiment for quality control. First, we excluded cell type populations with less than 60 observations due to sample size considerations. We flagged doublets and low quality cells as those with a mitochondrial gene ratio >10%, a ribosomal gene ratio >60%, or a hemoglobin gene ratio >60%, and filtered them out. Then the raw count data was normalized to counts per million and then log-transformed with ${\;\text{log}}_{2}(x+1)$.

For experiments using Scmap and Celltypist, the preprocessing concluded at this stage, in accordance with their original publications. For experiments with Torchnet, feature selection identified the top 2000 genes exhibiting the highest cell-to-cell variation using the dispersion-based method implemented in Seurat (Satija *et al.* 2015). Finally, we integrated the reference and query datasets using Harmony (Korsunsky *et al.* 2019) to correct for batch effects.

### 2.5 Evaluation metrics and experimental setup

The calibration set $D_{\mathrm{cal}}$ was set to 40% of the available training samples over all experiments. We first evaluated the performance of the anomaly detector through its statistical Power (Recall), FPR, and FDR. We used the well-established leave-one-cell-type-out strategy, i.e. exclude all observations of one cell type from the reference dataset, while they were retained in the query set.

The annotator outputs were assessed in terms of both overall performance and the quality of the conformal prediction. The quality of the set-valued annotations was quantified by measuring the average empirical coverage


(12) $$\text{Cov}=\frac{1}{n_{\text{test}}}\sum_{i=1}^{n_{\text{test}}}1(y\in C(x_{i})),$$

where $n_{\text{test}}$ is the query size. The class coverage gap is then defined as


(13) $$\text{CovGap}=\frac{1}{K}\sum_{c_{k}\in Y}|{\text{Cov}}_{k}-(1-\alpha )|,$$

where ${\text{Cov}}_{k}$ is the empirical class-conditional coverage for class $c_{k}\in Y$. The performance of the set predictions was measured through the average set size,


(14) $$\text{Average}\;\text{size}=\frac{1}{n_{\text{test}}}\sum_{i=1}^{n_{\text{test}}}|C(x_{i})|.$$

In addition to the query data, we reserved a small subset (10%) of reference samples as internal test set (containing no OOD samples). We also set aside another subset (15%) as a validation set to guide model training and prevent overfitting. The remaining samples were split into training and calibration subsets. The former subset was used to fit the underlying classifier and anomaly detector, whereas the latter was used to calibrate the conformal predictor.

We considered three nominal error levels for the annotator, namely α=[0.01,0.05,0.1]. For each experiment, different configurations were analysed. Each configuration included an error level, α, a non-conformity measure ($s_{\mathrm{APS}},s_{\mathrm{RAPS}},s_{\mathrm{THR}}$), a taxonomy (standard, classwise, cluster), and a leave-one-cell-type-out strategy. We set κ=1 and η=0.01 when employing the $s_{\mathrm{RAPS}}$ non-conformity score. The clustering procedure used in the *cluster* taxonomy was carried out by running *k*-means on the quantile-based non-conformity scores embedding, as in the original work (Ding *et al.* 2023), implemented by default in TorchCP (Huang *et al.* 2024). All configurations were repeated 20 times using different random seeds to ensure robust estimates. Minority cell groups were excluded from the reference dataset, but retained in the query set to simulate OOD observations. The anomaly detector was applied using the automatic $\alpha_{o}$ selection method described in Section S3.1, available as supplementary data at *Bioinformatics* online, except for experiment 1 of gastrula tissue, where an $\alpha_{o}=0.15$ was fixed due to sample size considerations.

## 3 Results

### 3.1 Anomaly detector accurately identifies OOD cells

**Figure 1.**
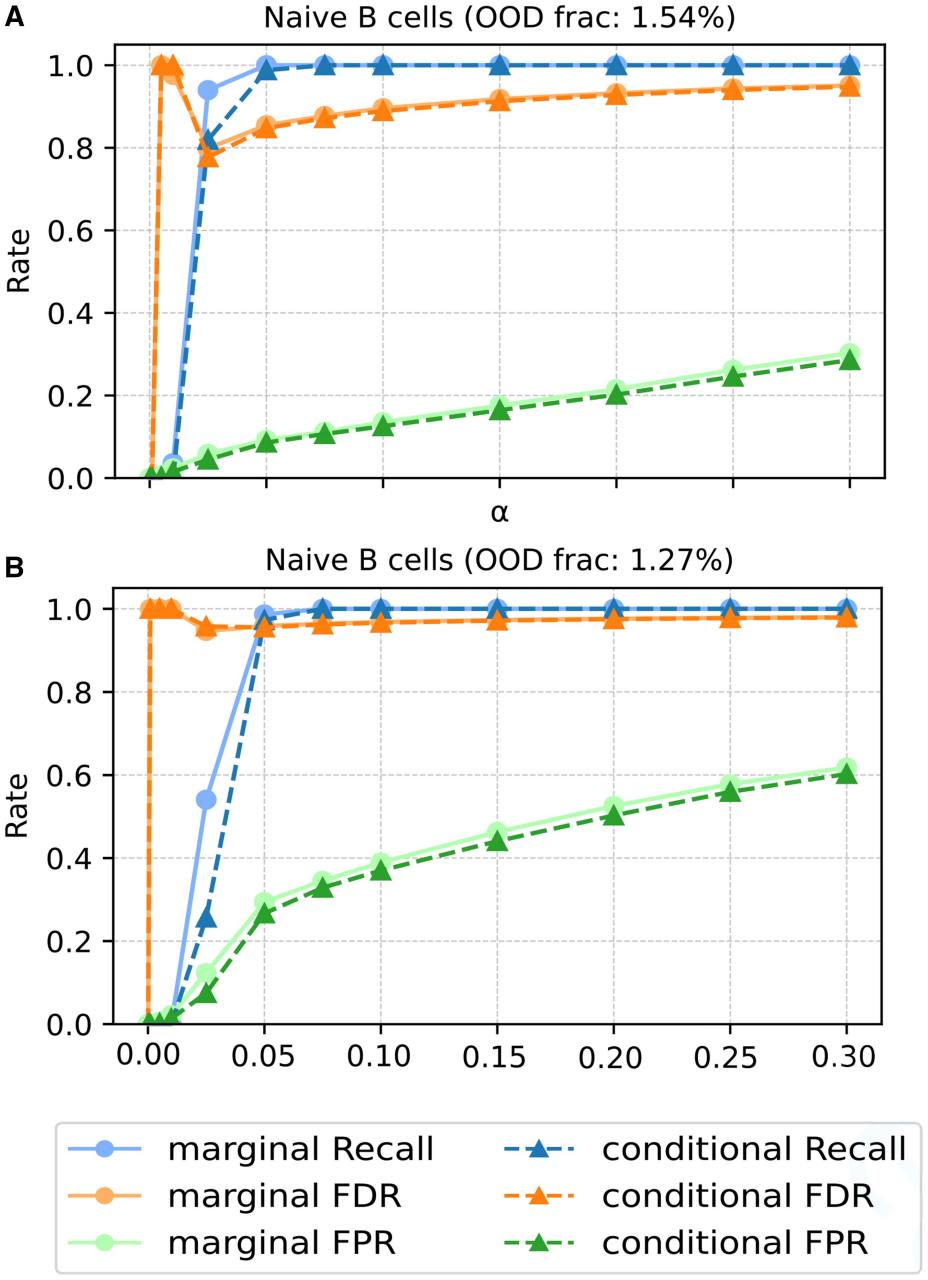
Performance of the anomaly detector in a leave-one-cell-type-out evaluation. Detection of *Naive B cells* is displayed for (A) a healthy query, and, (B) a query from human patients with lung tumors, representing 1.54% and 1.27% of the query cells, respectively. Metrics reported include statistical Power (blue), FDR (orange), and FPR (green). The fraction of true OOD cells among all the query cells is also shown. The $\alpha_{0}$ values range from $10^{-3}$ to 0.3.

Figure 1 presents marginal and conditional conformal *p*-values for Naive B cells flagged as OOD in the two lung-tissue queries considered. Over the range of significance levels tested $\alpha_{o}$, we observe that conformal *p*-values achieves the FPR guarantee given by Equation (5) in panel A, but not in panel B. This outcome is expected since observations represented in panel A were collected from healthy patients, whereas those from panel B were collected from patients with a lung tumor. As a consequence, the exchangeability assumption is broken between the reference and query in panel B. The conformalized detector effectively identifies OOD cells in both scenarios, achieving high power scores at low significance thresholds. A similar trend is observed for the rest of cell types tested in the leave-one-cell-type-out experiments (Figs S3 and S4, available as supplementary data at *Bioinformatics* online), as well as in the other tissues evaluated (Figs S5–S7, available as supplementary data at *Bioinformatics* online). To achieve a high power score is a critical step for the reliability of a conformalized single-cell annotator. A low power score inherently leads to numerous misclassified cells, which in turn can violate the coverage guarantees provided by the conformal prediction framework.

**Figure 2.**
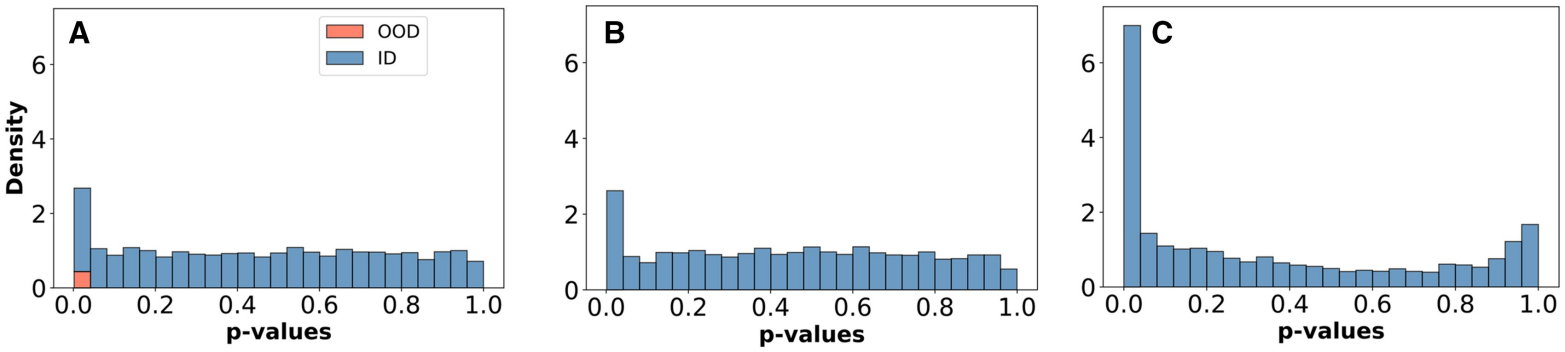
Probability distributions of marginal conformal *p*-values derived from the anomaly detector for the lung-tissue experiments. Blue bars indicate *p*-values derived from ID cells, while red bars depict *p*-values associated to the following OOD cells: *MNP/T doublets, Memory B cell*, and *Naive B cells*. Panels A and B display results for the healthy patients query, whereas Panel C corresponds to lung tumor patients. Panel A shows a scenario where the identified OOD cells are present.

The suboptimal FDR performance is related with the proportion of OOD cells in the query. As illustrated in Fig. 2A, the FDR is large when OOD cells are rare relative to ID cells. This phenomenon is a direct consequence of the statistical properties of conformal *p*-values: for exchangeable ID cells, these *p*-values follow a uniform distribution on [0, 1]. Consequently, a baseline proportion of ID cells will inherently receive low *p*-values. When the true OOD cell population is small, the number of correctly identified OOD cells is overwhelmed by this larger number of false-positive ID cells, leading to a high FDR.

### 3.2 Beyond OOD cell detection

A central challenge in single-cell analysis is the presence of covariate shifts between reference and query datasets. These shifts can arise from technical sources (e.g. batch effects) or biological variation (e.g. different tissues or disease states), compromising the exchangeability assumption required by conformal prediction. The statistical properties of the conformal *p*-values generated by our anomaly detector offers a principled way to assess whether the exchangeability is broken. By theoretical considerations, these *p*-values must be uniformly distributed on the interval [0, 1] when exchangeability holds. Any significant deviation from this uniformity in the distribution of the conformal *p*-values provides evidence of a broken exchangeability.

The results of the experiments carried out with immune cells highlight three possible scenarios (see Fig. 2). Specifically, Fig. 2A and B shows the *p*-value distribution for the healthy lung query dataset. In Fig. 2A, OOD cells are present, and the detector correctly identifies them as a very strange cells, i.e. they have an associated low *p*-value. In Fig. 2B, no OOD cells where included, however, the distribution of the *p*-values shows a modest peak of low *p*-values, suggesting small residual batch effects or subtle biological differences, while the rest of the distribution is uniform. Finally, Fig. 2C shows the distribution for the tumor lung query dataset. It clearly illustrates a major covariate shift due to the disease state of the patients, confirming that exchangeability is violated.

### 3.3 Uncertainty-aware cell type annotation results

**Figure 3.**
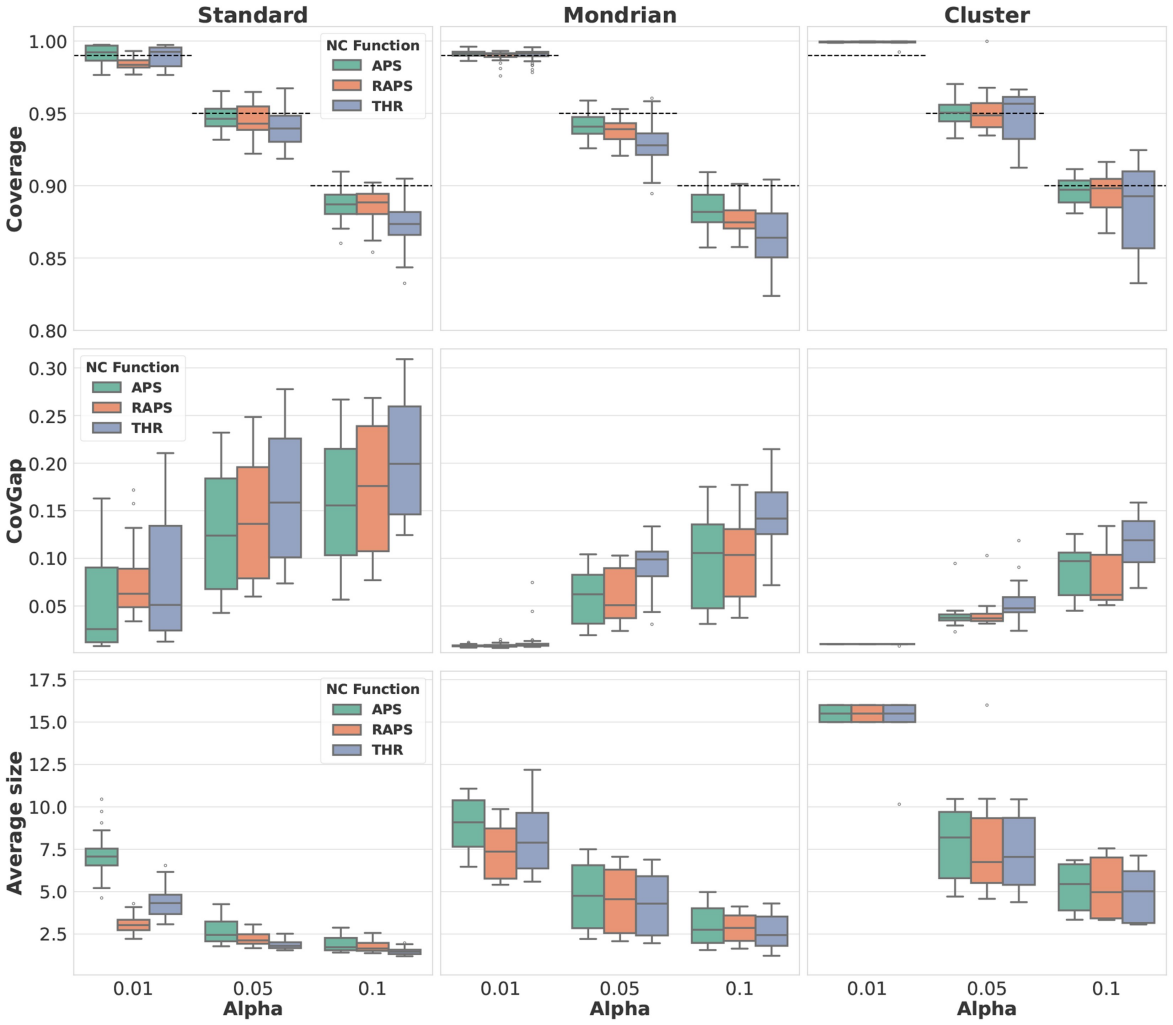
Average results for the breast tissue query data using TorchNet as undelying supervised model. The columns represent the three taxonomies: standard, classwise, and cluster. Each row displays the results for one of the three measured performance scores, namely coverage, covgap, and set size. Within each chart, every column corresponds to a nominal error level α=0.01,0.05,0.10, and the different non-conformity functions: $s_{\mathrm{APS}}$, $s_{\mathrm{RAPS}}$ and $s_{\mathrm{THR}}$, are indicated by different colors.

Figure 3 illustrates the average performance of the annotator in experiments conducted on breast tissue data using TorchNet, with the results stratified by error level, performance score, taxonomy, and non-conformity function. The results using Scmap and CellTypist as underlying models are reported in Figs S21 and S22, available as supplementary data at *Bioinformatics* online, respectively. Scmap performs well under the classwise and cluster taxonomies, likely due to its cluster-based classification approach, but fails under the standard taxonomy. CellTypist results do not achieve the coverage guarantee, probably due to the non-addressed technical variations in the preprocessing stage. By contrast, TorchNet achieves near-theoretical coverage across all taxonomies, with only minor deviations on the standard scheme likely due to a small fraction of OOD cells challenging to detect.

Global results for the experiments conducted using healthy lung, tumor‐patient lung, gastrula, and pancreatic tissues are provided in Figs S23–S26, available as supplementary data at *Bioinformatics* online. Although the observed variability is greater in the query data compared to the internal tests where OOD cells are absent and batch effects are negligible (see Figs S12–S15, available as supplementary data at *Bioinformatics* online), the coverage guarantees remain closely satisfied. Small deviations in the healthy lung-tissue experiment are observed. For this particular experiment, the reference/query pairs were taken from completely independent datasets. We could therefore expect that a small fraction of cells, even after the quality checks and the detection of anomalies, may still violate exchangeability. Nevertheless, coverage and performance scores remain notably closer to the theoretical values than in the tumor‐patient query where the biological batch clearly breaks the exchangeability assumption.

The robust uncertainty quantification provided by the prediction sets allows for enhanced downstream analyses: practitioners can control the overall annotation error rate, i.e. α, *a priori*. In addition, they can also identify which specific query observations are more challenging to annotate based on the size of their prediction sets, and take appropriate actions accordingly.

The annotation approach can be adapted according to the user’s requirements. For instance, when efficient set predictions (i.e. sets containing a single class) are desired, the standard taxonomy shown in Equation (1) is an appropriate choice. By contrast, in unbalanced reference datasets the coverage guarantee may not be fulfilled for rare cell types. Using the classwise taxonomy from Equation (10) ensures the coverage property is satisfied for every cell type in the reference dataset, see Fig. S16, available as supplementary data at *Bioinformatics* online, at the cost of generating larger prediction sets. Furthermore, calibration for extremely rare cell types can become unstable due to insufficient samples, resulting in higher CovGap score than with the standard taxonomy, see Figs S16–S19, available as supplementary data at *Bioinformatics* online. The cluster taxonomy provides a tradeoff between standard and classwise approaches. In addition, the cluster taxonomy shown in Equation (11) can also be tailored to the specific characteristics of the annotation problem (e.g. results of a hierarchical clustering in a sub-cell annotation problem). However, we report that the cluster taxonomy becomes unstable under very strict coverage requirements, e.g. α≥0.99, producing completely uninformative prediction sets.

Finally, the choice of a non-conformity score plays a role in the efficiency of the annotations. Although the $s_{\mathrm{THR}}$ function yields efficient set predictions, it may occasionally produce empty prediction sets. An empty set constitutes a rejection of the prediction that naturally emerges from the conformal prediction framework. This is an indication that the model found that no cell type meets the confidence requirement, reflecting that the model cannot make a reliable annotation for the query due to extreme uncertainty or changes in the distribution on X. In complex scenarios, we may be interested in a more adaptable-to-condition prediction tool. The prediction sets provided by $s_{\mathrm{APS}}$ and $s_{\mathrm{RAPS}}$ functions offer the advantage of considering the overall distribution of class probabilities in their construction, providing more informative and adaptive prediction sets when compared to $s_{\mathrm{THR}}$.

### 3.4 Conformal prediction for uncertainty-aware annotations: interpreting prediction sets

The cardinality of the prediction sets returned by a conformal predictor represents a measure of uncertainty for each query cell. In a human-in-the-loop scenario, this could lead to several benefits. For example, we could let the annotator automatically assign a cell type in cases where the conformal predictor is confident enough (e.g. in case of singleton predictions).

Beyond producing reliable measures of confidence, the returned sets contain the most probable types the query cell should belong to, which could speed the final identification. Let assume an annotated query observation whose prediction set contains three specific types: $c_{1}$, $c_{3}$, and $c_{5}$, out of K≫5 possible values. In that case, we could say that we have evidence at the significance level α that such query cell belongs to one of those types. Additionally, each type has a specific *p*-value associated, $p_{c_{1}}$, $p_{c_{3}}$, and $p_{c_{5}}$, which represents how much the query cell conforms with each of the types. This knowledge can be exploited in different ways, as in the case when a final annotation is needed, manually forcing the predictor to classify the query cell as the type with the highest associated *p*-value. Another possibility for a final cell type categorization is that an expert could perform an informed analysis on the differential expression between those possible types or compare the expression profiles of specific genes of relevance. In this way, the human workload can be significantly reduced, as experts no longer need to analyse the entire space of possible cell types but can focus on a much smaller, more relevant subset.

### 3.5 Limitations

**Figure 4.**
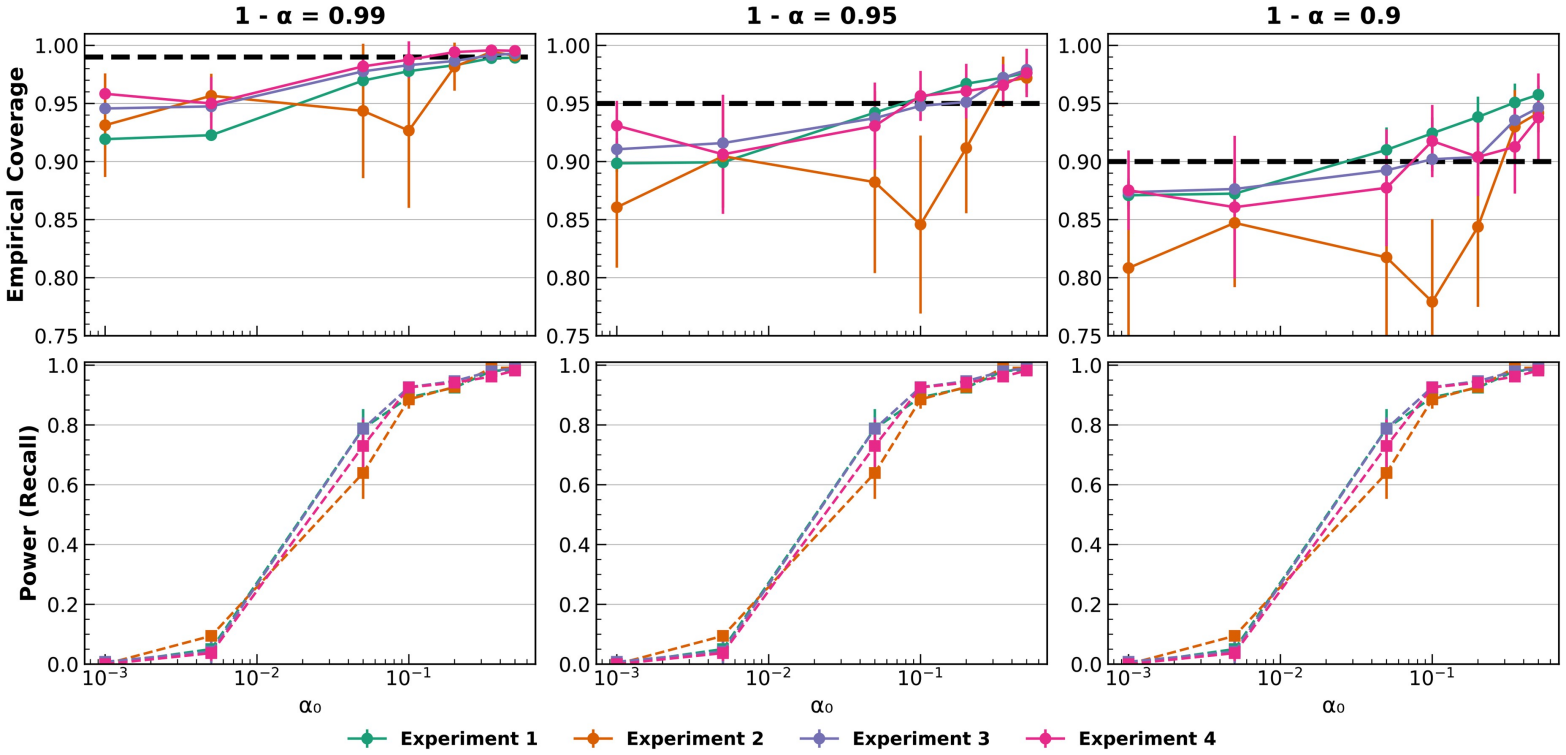
Power of the anomaly detector and empirical coverage behavior as a function of the significance threshold $\alpha_{o}$ in pancreatic-tissue experiments. All four experiments flag the same six cell types as OOD: *quiescent_stellate, macrophage, mast, schwann, epsilon* and *t_cell* thereby sampling different OOD distributions across experiments. Dashed horizontal black lines shows the expected theoretical coverage. $\alpha_{o}\in \{5\cdot 10^{-3},1\cdot 10^{-3},5\cdot 10^{-2},0.1,0.2,0.35,0.5\}$.

Despite the statistical advantages offered by our annotator based on conformal prediction, a poor recall performance in the anomaly detector can compromise the theoretical guarantees. Figure 4 illustrates this situation for the four experiments conducted using pancreatic tissue. At very low significance thresholds $\alpha_{o}$, the detector rarely flags OOD cells, yielding a pronounced gap between empirical and theoretical coverage. As $\alpha_{o}$ increases, the probability of detecting OOD cells grows, resulting in improved power scores, and the gap between empirical and theoretical coverage is progressively closed. We can also observe this trend in Fig. 4 for experiments 1, 3, and 4. Experiment 2, however, exhibits an interesting trend. As previously discussed, bad addressed ID cells also break the exchangeability assumption. For Experiment 2, at $\alpha_{o}=0.1$, our detector achieves relatively high power detecting those OOD cells which are present in the query, yet empirical coverage remains substantially below its theoretical value. Further increases in $\alpha_{o}$ lead to an abrupt elimination of this gap. This pattern suggests that a subset of ID cells violates the exchangeability assumption. Once these cells are removed using larger significance thresholds $\alpha_{o}$, the gap is closed. Finally, although the automatic $\alpha_{o}$ selector is based on a robust rationale for determining the optimal value of $\alpha_{o}$, be aware that its process may conclude before all OOD samples are identified. However, experiments conducted in Section S5.3, available as supplementary data at *Bioinformatics* online, show its capacity to largely mitigate or fully remove the impact of exchangeability violations when batch effects are present at different degrees (see Section S5.3, available as supplementary data at *Bioinformatics* online, for additional details).

## 4 Conclusions

This work presents a robust methodological approach for scRNA‐seq annotation with statistical guarantees. The approach, based in conformal prediction theory, effectively addresses two critical challenges for the annotation process: (i) the detection of OOD cells, and, (ii) well-calibrated uncertainty quantification for the automatic annotations. Conformal prediction ensures both reliability and trustworthiness of the results.

OOD cells are identified using a multiple hypothesis testing approach that leverages valid *p*-values derived from the conformal framework. These guarantees guard the proper construction of a valid null distribution to test whether a query cell belongs to the same distribution as the reference cohort. As a result, the anomaly detector provides a statistically grounded rationale for accepting or rejecting query cells at the established significance level. The uncertainty-aware scRNA‐seq annotator is afterwards applied. We evaluated a broad spectrum of different taxonomies and non-conformity functions and discussed the conditions under which they can be more advantageous to use. Final annotations are provided in the form of valid set predictions, each of which quantifies the uncertainty associated with each individual query cell through the length of the predicted set. This enhanced knowledge on the annotation process increases the confidence in the automatic annotations and provides confidence in further downstream analyses.

Future work will involve a further comparison across a wide range of well-established underlying models. Another interesting direction would be to study if introducing cluster-level information into the anomaly detector improves its performance on the most challenging to identify cell types. Our proposal is available through an open source repository including functions ready to be applied to scRNA‐seq annotation tasks.

## Supplementary Material

btaf521_Supplementary_Data

## Data Availability

The scripts supporting the findings of this study and a library with the annotator are available at https://github.com/digital-medicine-research-group-UNAV/conformalized_single_cell_annotator.
