# Supplementary material for: Conformal inference for reliable single cell RNA-seq annotation
Source: Bioinformatics. 2025 Sep 18;41(10):btaf521. doi: 10.1093/bioinformatics/btaf521 (PMC12506889; doi:10.1093/bioinformatics/btaf521)
Supplement: btaf521_Supplementary_Data [file btaf521_supplementary_data.pdf]

# Conformal Inference for Reliable scRNA-seq Annotation

Marcos López-De-Castro, Alberto García-Galindo,
José González-Gomariz, Rubén Armañanzas

**Supplementary Material**

## Contents

**S1 Conformal prediction in a nutshell** **2**
S1.1 Standard conformal prediction . . . . 2
S1.2 Mondrian conformal prediction . . . . 3
S1.3 Coverage vs. Efficiency vs. Adaptiveness . . . . 3

**S2 Conformal prediction for interpretable anomaly detection** **4**

**S3 Design and implementation** **6**
S3.1 Anomaly detector . . . . 6
S3.1.1 Underlying model . . . . 6
S3.1.2 Automatic $\alpha_o$ selection . . . . 9
S3.2 Underlying classification algorithm . . . . 9
S3.2.1 Scmap . . . . 10
S3.2.2 CellTypist . . . . 10
S3.2.3 TorchNet . . . . 10
S3.3 Conformal prediction implementation . . . . 11
S3.4 Annotations . . . . 11

**S4 Additional experimental details** **11**
S4.1 Cell label harmonization . . . . 11
S4.2 scRNA-seq raw data preprocessing . . . . 12
S4.3 Additional information . . . . 14

**S5 Anomaly detector experiments** **19**
S5.1 Expanded discussion about the FDR role in our anomaly detection problem . 19
S5.2 Results and extended discussion of the anomaly detector . . . . 19
S5.3 Testing the automatic $\alpha_o$ selector . . . . 21

**S6 Additional supporting results** **29**
S6.1 Tabular results . . . . 44

# S1 Conformal prediction in a nutshell

Conformal prediction is a machine learning framework for quantifying uncertainty in the predictions made by a statistical learning algorithm[1]. It produces reliable prediction sets that provide strong and distribution-free guarantees from the outputs of any machine learning model, regardless of their complexity or structure [2]. Although conformal prediction can be applied to any learning task, we here focus on classification tasks.

## S1.1 Standard conformal prediction

Let us initially assume a supervised classification setting and an available set of i.i.d. samples $(\mathbf{X}_i, y_i) \sim P, i = 1, \ldots, n$, in which each sample is formed by an input $\mathbf{X}_i \in \mathcal{X}$ (*e.g.*, a transcriptomic gene signature) and a class label $y_i \in \mathcal{Y}$ (*e.g.*, a specific cell type), from a distribution $P$ on $\mathcal{X} \times \mathcal{Y}$. The general goal of standard conformal prediction is to construct a set-valued predictor $\mathcal{C} : \mathcal{X} \to 2^{\mathcal{Y}}$ with the following ground truth coverage property:

$$\mathbb{P}\Big(y_{n+1} \in \mathcal{C}(\mathbf{X}_{n+1})\Big) \geq 1 - \alpha, \tag{1}$$

where $(x_{n+1}, y_{n+1})$ is a new test sample drawn from $P$ and $\alpha \in (0, 1)$ is a nominal error level. To this end, a central component of conformal prediction is the definition of a non-conformity score $s : \mathcal{X} \times \mathcal{Y} \to \mathbb{R}^+$, used to quantify the degree of relative uncertainty of the new sample with respect to a collection of samples. In practice, the non-conformity score is usually derived from the output of a supervised machine learning model. For example, we can consider a neural network and take $s(x_i, y_i) = 1 - \hat{f}_{y_i}(x_i)$ as the non-conformity score, where $\hat{f}_{y_i}(x)$ is the softmax output of the true class.

Despite conformal prediction was originally proposed within a transductive scheme [3], we work with a computationally efficient version, namely inductive/split conformal prediction [4]. In split conformal prediction, we first divide the training set into two disjoint sets:

- The proper training set $\mathcal{D}_{\textsf{train}}$, which is employed to learn a single predictive model $\hat{f}$.
- The calibration set, $\mathcal{D}_{\textsf{cal}}$, on which to compute the non-conformity scores:

$$s_i = s(x_i, y_i), \quad i \in \mathcal{D}_{\textsf{cal}} \text{ and } |\mathcal{D}_{\textsf{cal}}| = n_{cal} < n. \tag{2}$$

For a new test sample $\mathbf{X}_{n+1}$, we now can compute a valid conformal p-value for each $y \in \mathcal{Y}$ as follows:

$$p_y = \frac{1 + \sum_{i=1}^{n} \mathbb{1}\{s_i \geq s_{n+1}\}}{n_{\textsf{cal}}}. \tag{3}$$

This is a valid p-value because the i.i.d. property is required in the samples, forcing the non-conformity scores, $S = \cup\{s_i\}\ \forall i \in \mathcal{D}_{\textsf{cal}}$, to be exchangeable. From this, when $y$ is true, *i.e.*, $y = y_{n+1}$, the vector of non-conformity measures is uniformly distributed among $\{1, ..., n_{cal}+1\}$, so that $p_y$ is also uniformly distributed over $\{\frac{1}{n_{cal}+1}, ..., 1\}$. This translates into each rank position being attained with probability $1/(n_{cal}+1)$. Consequently, the probability that the non-conformity score associated with the new test sample $x_{n+1}$ is at least as large as rank $k$ with respect to the $n_{cal}$ calibration non-conformity scores is $k/(n_{cal}+1)$. For this

reason, $p_y$ is a valid p-value in the sense that $\mathbb{P}(p^y \leq \alpha) \leq \alpha$. Finally, to build the prediction interval with statistical guarantees $\mathcal{C}(\mathbf{X}_{n+1})$ at a confidence level of $1-\alpha$, we only need to accept all the trial values $y$ fulfilling:

$$\mathcal{C}(\mathbf{X}_{n+1}) = \{y : p_y \leq \alpha\}. \tag{4}$$

## S1.2 Mondrian conformal prediction

The ground truth coverage guarantees of standard conformal prediction are marginal, *i.e.*, on average over all the data, which in practice may lead to undercover some specific groups of samples (*e.g.*, low sample size cell populations on unbalanced data). To overcome this limitation, Mondrian conformal prediction was proposed as an extension of standard conformal prediction to construct confidence predictors with guarantees in a group-wise style. Mondrian conformal prediction mainly relies on performing the calibration step (*i.e.*, the computation of the non-conformity scores) separately on each of the categories given by some taxonomy. Formally, a taxonomy is a measurable function $\kappa : \mathcal{X} \times \mathcal{Y} \to \mathcal{K}$ that maps each $i$-th sample to a specific category $\kappa_i \in \mathcal{K}$, for a given discrete space $\mathcal{K}$.

As in standard procedure, Mondrian conformal prediction can be approached in a similar computationally efficient manner. Specifically, given a taxonomy $\kappa$, the split Mondrian conformal prediction computes a prediction set by means of a $\kappa$-conditional conformal p-value for each $\bar{y} \in \mathcal{Y}$, which is given by

$$p_{\bar{y}} = \frac{|\{i = 1, \ldots, m+1 : \kappa_i = \kappa_{m+1}, s_i > s_{m+1}\}|}{|\{i = 1, \ldots, m+1 : \kappa_i = \kappa_{m+1}\}|}, \tag{5}$$

where $s_i$ and $\kappa_i$ are the non-conformity score and the category related to the $i$-th calibration sample, respectively.

The prediction sets produced by a Mondrian conformal prediction satisfy validity conditioned on each of the categories defined by the chosen taxonomy. For example, we can consider the classwise taxonomy $\kappa : \mathcal{X} \times \mathcal{Y} \to \mathcal{Y}$, which maps each sample to the label space. In this case, the resulting set-valued predictor achieves class-conditional coverage guarantees:

$$\mathbb{P}\Big(y_{n+1} \in \mathcal{C}(x_{n+1}) \mid y_{n+1} = y\Big) \geq 1-\alpha, \quad \text{for all } y \in \mathcal{Y} \tag{6}$$

## S1.3 Coverage vs. Efficiency vs. Adaptiveness

Although the marginal coverage guarantee provided by conformal prediction is a desirable property for any statistical learning algorithm, one might aspire to achieve the stronger notion of conditional coverage:

$$\mathbb{P}\Big(y_{n+1} \in \mathcal{C}(x_{n+1}) \mid x_{n+1} = X\Big) \geq 1-\alpha. \tag{7}$$

Achieving that conditional coverage in a distribution-free setting is challenging. However, approximate conditional coverage can be reached while maintaining marginal coverage. To this end, Romano *et al.* [5] introduced the Adaptive Prediction Sets (APS), an approach to defining non-conformity functions aimed at approximating conditional coverage. The rationale is to adapt the size of the prediction sets based on the model's confidence: larger sets

for hard-to-classify examples and smaller sets for confident predictions. This is accomplished by sorting the predicted class probabilities in descending order and including classes in the prediction set until the cumulative probability exceeds a predefined threshold corresponding to the desired confidence level $\alpha$. Angelopoulos *et al.* [6] later presented an evolution, namely Regularized Adaptive Prediction Sets (RAPS), to address certain limitations inherent in the APS non-conformity function. By introducing a regularization component, RAPS penalizes the inclusion of unlikely classes in the cumulative probability, thereby enhancing the adaptivity of the conformal predictors and improving robustness in long-tailed distributions at the expense of including two extra hyperparameters $\kappa_{reg}$ and $\lambda$. The parameter $\kappa_{reg}$ represents the number of classes in the prediction set from which an extra $\lambda$ penalty is applied compared with APS. Although these parameters can be inferred using the procedure outlined by the authors in [6], we chose to penalize prediction sets larger than one, *i.e.*, $\kappa_{reg} = 1$, as singleton predictions are preferred for cell annotation, and a small penalty $\lambda = 0.01$ to maintain flexibility and promote adaptiveness.

# S2 Conformal prediction for interpretable anomaly detection

This supplementary Section focuses on the use of the conformal prediction framework for anomaly detection, where anomalies in our context are defined as cells not present in the reference data. This fully nonparametric approach determines whether test observations originate from the same underlying distribution as the reference data, without imposing any specific modeling assumptions. The rationale for the inclusion of this mechanism lies in the potentially large number of cells present in the query data, but absent from the reference data, a well-known limitation of single-cell annotation methods [7, 8]. In addition, single-cell data are particularly affected by batch effects as well as potentially biological confounders, like state-dependent cellular variations. The conformal anomaly detection framework thus provides a practical tool for identifying and addressing these scenarios where exchangeability may be broken. Conformal anomaly detection was first proposed by Laxhammar *et al.* [9] and has been recently revisited by Bates *et al.* [10].

Formally, out-of-distribution (OOD) detection-also referred to as anomaly detection or novelty detection, involves determining whether each sample in a test dataset, $\mathcal{D}_{test}$, is drawn from the same underlying distribution, $P_X$, that characterizes an observed dataset $\mathcal{D}_{ref}$ [11]. Samples not following $P_X$ are known as outliers, whereas those following $P_X$ are considered inliers. Despite the sophisticated and complex prediction algorithms developed to accomplish this task [11], such algorithms are not backed by any statistical guarantee for their predictions. Therefore, the main idea behind conformal anomaly detection is to construct valid p-values for nonparametric outlier detection, in the sense that we can leverage the power of statistical hypothesis testing to determine whether a novel observation is an outlier or an inlier. We are thus interested in testing the following null hypothesis: $\mathcal{H}_{0,i} : X_i \sim P_X$, for any $X_i \in \mathcal{D}_{test}$. Conformal anomaly detection returns a valid p-value for this hypothesis. First, we define an anomaly scoring function $\hat{s}_o : \mathcal{X} \rightarrow \mathbb{R}$ and split the reference data into mutually exclusive training and calibration sets, as detailed in the previous Supplementary Section S1.1. Any one-class model fitted on the training set is a strong candidate to serve as anomaly scoring function. A one-class model is a machine learning model trained on data with only a single class [11].

After fitting the model, we apply it to the calibration set to obtain non-conformity scores $s_i = \hat{s}_o(X_i),\ X_i \in D_{cal}$. Larger scores $s_i$ imply higher likelihood for $X_i$ to be an anomaly. To test whether an unseen cell $X_{n+1} \in \mathcal{D}_{test}$ from the query set is out-of-distribution relative to the reference data, we use Equation (3) to compute a p-value for that particular cell $p_{n+1}$. Intuitively, this p-value quantifies how extreme the new cell is relative to the calibration set. Since the calibration samples are exchangeable with the training samples, the non-conformity scores $\hat{s}(D_{cal})$ follow a uniform distribution. This implies that, if the test sample is also exchangeable with the calibration set, the conformal p-value, $p_{n+1}$, is a marginally valid p-value for the hypothesis $\mathcal{H}_{0,n+1} : X_{n+1} \sim P_X$ because is uniformly distributed on the set $\{1/(n+1), ..., 1\}$, which guarantees that

$$\mathbb{P}\Big(p_{n+1} \leq \alpha\ \Big) \leq \alpha,\ \forall \alpha \in (0,1), \tag{8}$$

when exchangeability holds. This provides control of the Type I error on average, meaning the expected False Positive Rate (FPR) across all possible draws of the calibration set $\mathcal{D}_{\text{cal}}$ is bounded by $\alpha$:

$$\mathbb{E}_{\mathcal{D}_{\text{cal}}}[FPR(\alpha, \mathcal{D}_{\text{cal}})] \leq \alpha. \tag{9}$$

However, this marginal guarantee can be insufficient for a single experiment. An "unlucky" draw of $\mathcal{D}_{\text{cal}}$ could lead to an FPR substantially higher than the nominal level $\alpha$. Stronger guarantees were proposed by Bates et al. [10] introducing conformal p-values that satisfies the property of calibration-conditional validity. This property ensures that, with probability $1 - \delta$, the predicted p-values are valid conditional on the specific calibration set used:

$$\mathbb{P}\left[\mathbb{P}\Big(p_{n+1} \leq \alpha \mid \mathcal{D}_{\text{cal}}\Big) \leq \alpha, \text{for any ID sample } X_{n+1} \text{ and } \forall \alpha \in (0,1)\right] \geq 1 - \delta. \tag{10}$$

In addition, conditional p-values provide a more appropriate set of p-values for the standard procedures to control the FDR level, such as the Benjamini–Hochberg correction [10]. Unfortunately, the toll for the user is that they tend to decrease the statistical power of the overall anomaly detection, *i.e.*, the number of detected true anomalies.

Our code includes the option to use this conditional methodology. Specifically, the conditional p-value adjustment can be induced following Theorem 4 from [10]. The central idea of this approach is to identify an appropriate adjustment function that yields conditionally valid p-values. For a more comprehensive discussion on the theoretical and methodological details, we refer the reader to the original publication (see Section 3). We also implemented the Monte Carlo adjustment procedure, as it unifies the advantages of two analytical approaches proposed in the same paper [10].

# S3 Design and implementation

Figure S1 shows the scheme of our methodology. Code for the annotation tool is available at https://github.com/digital-medicine-research-group-UNAV/conformalized_single_cell_annotator

## S3.1 Anomaly detector

### S3.1.1 Underlying model

Numerous classical models for anomaly detection, such as one-class SVM and isolation forest, have been developed. However, reports in the literature indicate these may not be the most suitable options for high-dimensional or complex data [12, 13, 14]. We implemented an unsupervised anomaly scoring function based on the reconstruction error of an autoencoder (AE), an approach previously used by [15]. The underlying idea of AE is to learn an efficient low-dimensional representation of typical samples and identifying those samples that deviate significantly from this learned manifold. The AE architecture comprises two feed-forward, multilayer neural networks: an encoder and a decoder. The encoder maps the original input data $\mathbf{X} \in \mathbb{R}^p$ to a reduced latent space $Z \in \mathbb{R}^{p'}$, where $p' < p$. The decoder maps the reduced space back again to the original space, yielding reconstructed data $\mathbf{X}' \in \mathbb{R}^p$. Both networks are jointly trained to minimize the reconstruction error between $\mathbf{X}$ and $\mathbf{X}'$. Typically, this error is quantified using a loss function based on a distance metric.

Our architecture first applies per-sample Layer Normalization to remove the dependency on mini-batch statistics. During training we included in each normalized vector an additive isotopic Gaussian noise ($\mathcal{N}(0, , \sigma^2)$ with $\sigma = 0.1$), which encourages the model to learn stable representations robust to minor perturbations [16]. Both networks, encoder and decoder, are jointly trained to minimize the following loss function designed to enhance the robustness of the induced representation through multiple regularization terms:

$$\mathcal{L} = \mathcal{L}_{\text{recon}} + \lambda_{\text{sparse}}|Z| + \lambda_{\text{contract}}\|J_f(x)\|_F^2, \tag{11}$$

where:

- $\mathcal{L}_{\text{recon}}$ is implemented through the *torch.nn.SmoothL1Loss()* pytorch class. It is sensitive to outliers and may prevent exploding gradients.
- $\lambda_{\text{sparse}}$ encourages the model to activate only a subsets of neurons via an $L_1$ penalty, resulting in a more efficient representations.
- $\lambda_{\text{contract}}\|J_f(z)\|_F^2$ term enhances the robustness of the detector to small perturbations [17].

The AE used in our experiments consisted of two symmetric encoder and decoder networks, each wrapped with weight normalization, followed by a Sigmoid Linear Unit (SiLU) activation function. Dropout regularization is also applied after each activation layer to mitigate overfitting. Training was performed with the AdamW optimizer with decoupled weight decay for better generalization. Finally, samples are scored by their mean squared reconstruction error (MSE):

$$MSE = 1/n \sum_{i=1}^{n} (\mathbf{X}_i - \mathbf{X}'), \tag{12}$$

between input and output. Higher reconstruction errors indicate a greater likelihood of being an outlier. The model hyperparameters can be manually tunned to adapt the detector to specific datasets. All code is comptaible with the PyTorch library.

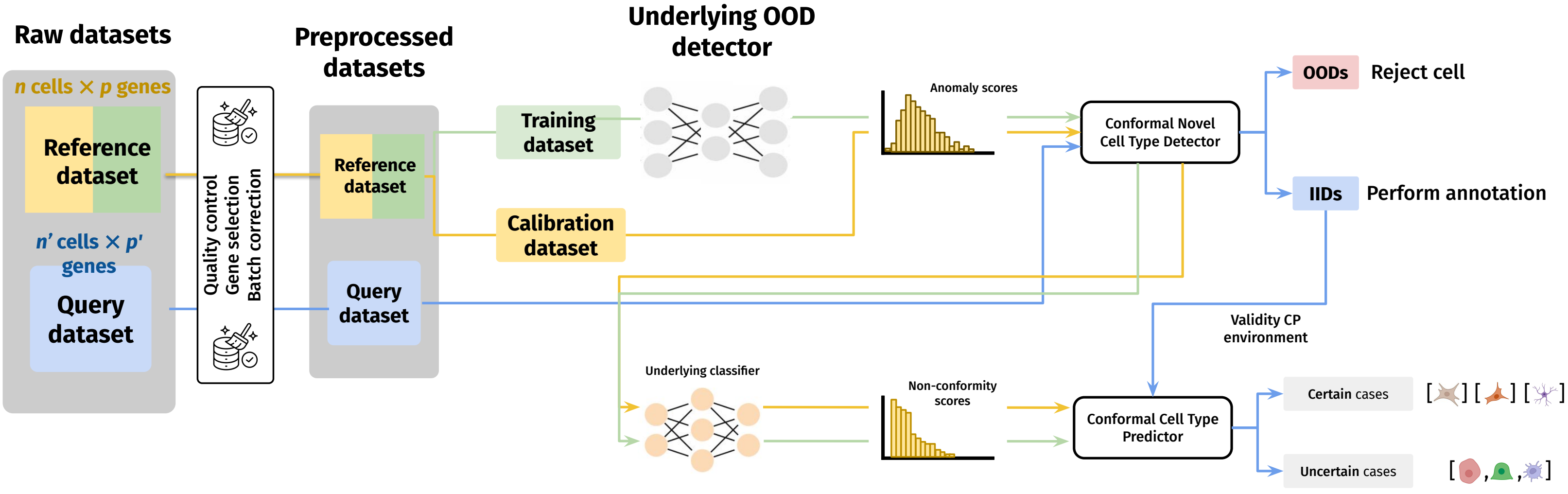


Figure S1: Overview of the proposed sequential machine learning pipeline. Green boxes and lines correspond to training data, yellow to calibration data, and blue to query data. After preprocessing the datasets, the reference data splits into $D_{train}$ and $D_{cal}$. Next, the anomaly detector is trained, and out-of-distribution cells are filtered from the query data. Finally, our uncertainty-aware annotator provides reliable predictions in the form of valid prediction sets.

### S3.1.2 Automatic $\alpha_o$ selection

This Supplementary Subsection describes a procedure for selecting an appropriate $\alpha_o$ for the anomaly detector, instead of fixing it *a priori.*

When the exchangeability assumption is violated, the marginal conformal $p$-values are no longer uniformly distributed (see Figure 1 of the main manuscript). Nevertheless, a subset of observations can still be i.i.d. and remain exchangeable. Our goal is therefore to identify the smallest significance level $\alpha_o \in [0,1]$ such that, after discarding all observations whose conformal $p$-value fall below $\alpha_o$, the empirical distribution of the remaining query $p$-values becomes statistically indistinguishable from an i.i.d. sample drawn from the reference data. The procedure is summarized in Algorithm 1.

**Algorithm 1** Greedy search for a suitable $\alpha_o$.

**Input:** Query $p$-values $\mathcal{P}^{\text{query}} = \{p_i\}_{i=1}^{n}$; $p$-values from an unseen sample from the reference $\mathcal{P}^{\text{test}} = \{p_i^{\text{test}}\}_{i=1}^{m}$; Number of candidates $K$; Significance level $\gamma$.
**Initialize:** $\alpha_{best} \leftarrow$ `None`; $\alpha_{max} \leftarrow 0.5$; List $\boldsymbol{\alpha} \leftarrow \{\alpha_k = k/K \mid k = 0, \dots, K-1\}$.
**for** $\alpha \in \boldsymbol{\alpha}$ **do**
    $S_{\text{q}} \leftarrow \{p \in \mathcal{P}^{\text{query}} : p \geq \alpha\}$
    $S_{\text{t}} \leftarrow \{p \in \mathcal{P}^{\text{test}} : p \geq \alpha\}$
    $\tilde{S}_{\text{q}} \leftarrow \{(p-\alpha)/(1-\alpha) : p \in S_{\text{q}}\}$
    $\tilde{S}_{\text{t}} \leftarrow \{(p-\alpha)/(1-\alpha) : p \in S_{\text{t}}\}$
    $p_{\text{KS}} \leftarrow$ `Kolmogorov-Smirnov`$(\tilde{S}_{\text{q}}, \tilde{S}_{\text{t}})$
    **if** $p_{\text{KS}} \geq \gamma$ **then**
        $\alpha_{best} \leftarrow \alpha$; **break**
    **end if**
    **if** $\alpha > \alpha_{max}$ **then break**
    **end if**
**end for**
**Output:** $\alpha_o = \alpha_{best}$; `warn` if $\alpha_{best} =$ `None`.

The resulting $\alpha_o$ can be used throughout the conformalized anomaly detector: all $p$-values below $\alpha_o$ are flagged as OOD cells, thereby approximately restoring uniformity and enabling valid downstream inference. The accuracy of this procedure improves as function of the calibration dataset size. If this size is no larger enough, it can result in a "sharpened" distribution. If the practitioner suspects the presence of heavy batch effects or OOD observations, the option of halting the process at $\alpha_{max} = 0.5$ can be deactivated or amplified to, for example, $\alpha_{max} = 0.9$.

## S3.2 Underlying classification algorithm

Conformal inference guarantees are independent of the underlying model employed. We provide experiments with three different models: Scmap, Celltypist, and a Pytorch-based feed-forward network, we refer to as "TorchNet". The three algorithms are implemented in the code repository.

#### S3.2.1 Scmap

Scmap is a method for projecting cells from a scRNA-seq experiment onto cell types identified in a different experiment [18]. It is particularly useful for cross-dataset comparisons and integrating data from different scRNA-seq experiments. We have implemented Scmap in Python and wrapped it in a PyTorch-compatible class to facilitate fast conformal prediction calibration through torchCP [19]. We have implemented the scmap-cell algorithm described in the original publication. The method expects genes already selected and carrying their original meaning-*i.e.*, non-embedded data. Thus, the data could be stored in the adata.X object or in adata.layer["name_of_the_layer"].

#### S3.2.2 CellTypist

CellTypist performs automated cell type annotation using logistic regression classifiers optimized by stochastic gradient descent [20]. It can be used with pretrained models; however, in our implementation, we focus on training user-provided references. This tool supports raw log-normalized data. Similar to Scmap, we have wrapped CellTypist in a PyTorch-compatible class to enable fast conformal prediction calibration through TorchCP.

#### S3.2.3 TorchNet

We implemented a feed-forward deep neural network in PyTorch $\hat{f} : \mathcal{X} \to \Delta^k$ as the underlying multi-class classifier. This classifier outputs, for each new cell, a softmax score for each of the $K$ types. We selected this approach due to its adaptability and ability to handle large-scale data effectively. The network is trained on $\mathcal{D}_{\text{train}}$ to minimize a weighted categorical cross-entropy loss, where weights are inversely proportional to cell-type frequencies alleviating possible class imbalance. The network employs ReLU activations and uses Adam optimizer. The learning rate is reduced by a factor of 0.1 if the validation loss does not decrease for three consecutive epochs, and early stopping is implemented to prevent overfitting. In the provided annotator, users can define the network architecture, number of neurons per layer, dropout rates, batch size, and initial learning rate.

The general structure is as follows:

- **Input layer**: The size of the layer is equal to the number of genes given.
- **Hidden layers:** Each hidden layer is implemented as a fully connected layer. Then, a batch normalization layer is applied. This helps stabilize and accelerate the training by reducing internal covariate shift. Dropout is applied after batch normalization at the specified percentage to mitigate possible overfitting. Non-linear transformations are introduced by applying the ReLU activation function, which is a commonly used activation function in neural networks training.
- **Output layer:** The final layer is a linear mapping from the last hidden layer's size to the number of unique classes, *i.e.*, cell types, yielding logits for each class.

We trained the network parameters by minimizing the weighted categorical cross-entropy loss. Class weights $\omega_k$ are computed using a balanced strategy and are inversely proportional

to the frequency of each cell type in the training data. The loss function is defined as

$$\mathcal{L}(y, \hat{y}) = \lambda \sum_{k=1}^{K} \omega_k \cdot y_k \, \log \hat{y}_k, \tag{13}$$

where $y$ and $\hat{y}$ denote true and predicted cell type, $K$ is the total number of classes, and $\lambda$ is a scaling factor set to 1. Model parameters are optimized using the Adam optimizer with an initial learning rate specified by the user (we used $10^{-4}$). To improve convergence, a learning rate scheduler monitors the validation loss and reduces the learning rate by a factor of 0.1 if no improvement is observed over 3 epochs. Early stopping is also implemented with patience to avoid overfitting. GPU-accelerated training is available.

### S3.3 Conformal prediction implementation

After training, the model is calibrated building a conformal predictor using a separate calibration dataset. We implemented the conformal prediction methodology using TorchCP [19], which allows for fast conformal calibration in python. Non-conformity functions and taxonomies are provided by the user.

### S3.4 Annotations

The scheme established in Figure S1 returns an `adata` object with the cell type annotations in `adata.obs`. Specifically, for each nominal error level $\alpha$, the conformal predictor returns a valid set prediction at the $\alpha$ level. We also provide the p-values associated to each possible class as well as the classical outcome provided by the underlying model.

## S4 Additional experimental details

In this Supplementary Section, we provide additional information about the data used in our experiments.

### S4.1 Cell label harmonization

Cell type labels between each reference–query pair for pancreas, breast and gastrula tissues are consistent. However, cell type labels for the lung experiments require harmonization in order to use uniform cell type labels between the reference and the query.

To harmonize lung cell type annotations across datasets, we adopted the nomenclature from the reference atlas and created the mapping summarized in Tables S1 and S2. The harmonization process involved three stages. First, we automatically relabeled cell populations with unambiguously corresponding names. Most of the harmonization process is on this part. In the case of the query of healthy donors these were *Classical Monocytes, Non-classical Monocytes, Natural Killer cells, Basophils/Mast cells, Dendritic cells, Macrophages, Plasma cells,* and *Proliferating NK/T cells*; in tumor donors they were: *B cells memory, Plasma cells, Macrophages M0, Monocytes, Macrophages M2, Dendritic cells resting, T cells regulatory (Tregs), Dendritic cells activated, Mast cells activated, B cells naive* and *T cells CD4 naive.* Secondly, cell types with no counterpart in the reference were excluded. They

were *Neutrophils, Mast cells resting* and *Eosinophils*. Finally, for ambiguous cell types that could potentially map to multiple reference populations, *i.e.*, *B cells, CD8+ T cells, CD4+ T cells,* and *Platelets* for healthy samples, and *Macrophages M0, Macrophages M2, T cells CD4 memory resting, T cells CD8, NK cells resting* and *NK cells activated* in tumor samples, we performed a targeted annotation. We ran CellTypist on these specific subsets and assigned the most likely identity based on an examination of the resulting UMAP visualizations.

| **Original label name** | **Harmonized label** |
|---|---|
| Natural Killer | NK_CD16+(1318) |
| Classical Monocyte | Classical monocytes (869) |
| CD4+ T | Trm_Th1/Th17 (529)<br>Teffector/EM_CD4 (461) |
| Nonclassical Monocyte | Nonclassical monocytes (468) |
| Basophil/Mast | Mast cells (387) |
| CD8+ T | Trm_Th1/Th17 (376) |
| Dendritic | DC2 (289) |
| Macrophage | Alveolar macrophages (267) |
| B | Memory B cells (197)<br>Naive B cells (83) |
| Plasma | Plasma cells(134) |
| Proliferating NK/T | Cycling T&NK (66) |
| Proliferating Immune | Cycling (51) |
| Neutrophil | — |
| Platelet | MNP/T doublets (11)<br>Mast cells (8) |

Table S1: Map between reference and lung healthy query set.

## S4.2 scRNA-seq raw data preprocessing

We here provide additional details about the preprocessing pipeline. For each dataset, cell type populations with less than 45 observations were excluded due to sample size considerations. Then, we flagged doublets and low quality cells as those with a mitochondrial gene ratio $> 10\%$, a ribosomal gene ratio $> 60\%$, or a hemoglobin gene ratio $> 60\%$, and filtered them out. Potential doublets were identified and also rejected from further analysis using the Scrublet algorithm [21], with additional safeguards to exclude cells yielding non-finite doublet scores. Genes detected in fewer than three cells were also removed to reduce technical noise. Prior to downstream processing, count matrices were normalized to 10,000 counts per cell, and then log-transformed $\log_2(x+1)$. Subsequently, we regressed out the effects of mitochondrial gene content to mitigate technical confounding and scaled the data to unit variance, clipping extreme values at $\pm 10$. Batch and donor-specific effects, as well as confounding variables (*e.g.*, sequencing, chemistry), were corrected via ridge regression as implemented in BBKNN package [22].

| Original label name | Harmonized label |
|---|---|
| T cells CD4 memory resting | Teffector/EM_CD4+(1372) |
| B cells memory | Memory B cells(1051) |
| Plasma cells | Plasma cells (832) |
| Macrophages M0 | Alveolar macrophages (773) |
| Monocytes | Classical monocytes (474) |
| Macrophages M2 | Intermediate macrophages (413) |
| T cells follicular helper | Trm_Th1/Th17 (330) |
| Dendritic cells resting | DC2 (295) |
| T cells regulatory (Tregs) | Tregs (246) |
| T cells CD8 | Trm/em_CD8 (210) |
| Dendritic cells activated | migDC (141) |
| Mast cells activated | Mast cells (236) |
| NK cells resting | NK_CD16+ (111) |
| B cells naive | Naive B cells (74) |
| Macrophages M1 | MNP/T doublets (61) |
| NK cells activated | NK_CD56bright_CD16- (24) |
| T cells CD4 naive | Tnaive/CM_CD4 (4) |
| Mast cells resting | – |
| Mast cells resting | – |
| Eosinophils | – |
| Neutrophils | – |

Table S2: Map between reference and lung tumoral query set.

Feature selection was performed on the reference dataset by identifying the top 2,000 most relevant genes using the Seurat v3, focusing on genes with the greatest cell-to-cell expression variability. Prior to reference-query data integration, query datasets were filtered to retain only genes highly expressed in the reference set. The integration of reference and query datasets was carried out using Harmony [23] to correct for batch effects. The preprocessing steps were conducted using the Scanpy Python package [24].

In the case of experiments with samples from pancreatic tissue, we identified and excluded a minor subset of ductal cells. We observed that two distinct ductal clusters emerged in UMAPs, see S2, one of which contained a mixture of ductal and acinar cells. Quality-control analysis revealed that ductal cells in this mixed cluster exhibited a significantly higher number of detected genes ($p \ll 0.05$) than acinar cells but not elevated total counts. This may indicate ductal-cell doublets. In addition, we observed evidence of Acinar-to-Ductal Metaplasia (ADM): the mixed cluster co-expressed high levels of the acinar marker CELA3B, strong MUC1, and low KRT19, indicative of transitional cells ($p \ll 0.05$). These samples were removed from downstream analyses due to the difficulty of re-classify them.

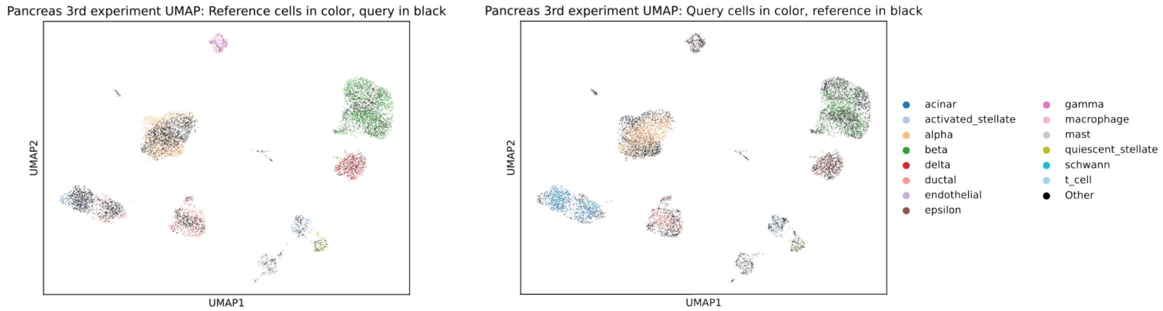


Figure S2: UMAP visualizations from the third pancreatic tissue experiment. Left panel: query cells highlighted in color; right panel: reference cells highlighted in color.

## S4.3 Additional information

Table S3 shows additional information about the reference-query scRNA-seq datasets used in the experiments. Tables S4, S5, S6, and S7 present the cell types distribution in each experiment. Supplementary Table S8 shows the cell types used to simulate OOD observations in each experiment used to assess the annotator performance.

| **Tissue** | **Experiment** | **Ref/Que** | **Nº obs** | **Nº Genes** | **Nº Cell types** |
|---|---|---|---|---|---|
| lung | Healthy | Reference | 24,951 | 1,693 | 25 |
| | | Query | 5,378 | 1,693 | 13 |
| | Lung tumoral | Reference | 24,967 | 1,740 | 26 |
| | | Query | 5,815 | 1,740 | 16 |
| Breast | 1 | Reference | 31,102 | 1,993 | 19 |
| | | Query | 18,288 | 1,993 | 19 |
| | 2 | Reference | 18,288 | 1,993 | 19 |
| | | Query | 31,102 | 1,993 | 19 |
| Gastrula | 1 | Reference | 1,843 | 1,986 | 14 |
| | | Query | 4,394 | 1,986 | 14 |
| | 2 | Reference | 4,394 | 1,986 | 14 |
| | | Query | 1,843 | 1,986 | 22 |
| Pancreas | 1 | Reference | 6,534 | 1,574 | 14 |
| | | Query | 1,903 | 1,574 | 14 |
| | 2 | Reference | 6,706 | 1611 | 14 |
| | | Query | 1,705 | 1,611 | 14 |
| | 3 | Reference | 4,725 | 1693 | 14 |
| | | Query | 3,518 | 1,693 | 14 |
| | 4 | Reference | 7141 | 1464 | 14 |
| | | Query | 1,279 | 1,464 | 14 |

Table S3: scRNA-seq datasets used in the experiments. The information corresponds with post-processed datasets.

| Cell Type | Healty | | Lung tumoral | |
|---|---|---|---|---|
| | **Ref** | **Query** | **Ref** | **Query** |
| Alveolar macrophages | 11,094 (44.5%) | 267 (5.0%) | 11,101 (44.5%) | 773 (13.3%) |
| Classical monocytes | 2,601 (10.4%) | 869 (16.2%) | 2,601 (10.4%) | 474 (8.2%) |
| Cycling | 162 (0.6%) | 51 (0.9%) | 162 (0.6%) | 0 (0.0%) |
| Cycling T&NK | 181 (0.7%) | 66 (1.2%) | 181 (0.7%) | 0 (0.0%) |
| DC1 | 75 (0.3%) | 0 (0.0%) | 75 (0.3%) | 0 (0.0%) |
| DC2 | 278 (1.1%) | 289 (5.4%) | 278 (1.1%) | 295 (5.1%) |
| Intermediate macrophages | 1,956 (7.8%) | 0 (0.0%) | 1,956 (7.8%) | 413 (7.1%) |
| MAIT | 111 (0.4%) | 0 (0.0%) | 111 (0.4%) | 0 (0.0%) |
| MNP/T doublets | 440 (1.8%) | 11 (0.2%) | 449 (1.8%) | 61 (1.0%) |
| Mast cells | 550 (2.2%) | 395 (7.3%) | 550 (2.2%) | 236 (4.1%) |
| Memory B cells | 94 (0.4%) | 197 (3.7%) | 94 (0.4%) | 1,051 (18.1%) |
| NK_CD16+ | 2,260 (9.1%) | 1,318 (24.5%) | 2,260 (9.1%) | 111 (1.9%) |
| NK_CD56bright_CD16- | 406 (1.6%) | 0 (0.0%) | 406 (1.6%) | 24 (0.4%) |
| Naive B cells | 69 (0.3%) | 83 (1.5%) | 69 (0.3%) | 74 (1.3%) |
| Nonclassical monocytes | 649 (2.6%) | 466 (8.7%) | 649 (2.6%) | 0 (0.0%) |
| Teffector/EM_CD4 | 821 (3.3%) | 461 (8.6%) | 821 (3.3%) | 1,372 (23.6%) |
| Tem/emra_CD8 | 633 (2.5%) | 0 (0.0%) | 633 (2.5%) | 0 (0.0%) |
| Tgd_CRTAM+ | 99 (0.4%) | 0 (0.0%) | 99 (0.4%) | 0 (0.0%) |
| Tnaive/CM_CD4 | 165 (0.7%) | 0 (0.0%) | 165 (0.7%) | 4 (0.1%) |
| Tregs | 220 (0.9%) | 0 (0.0%) | 220 (0.9%) | 246 (4.2%) |
| Trm/em_CD8 | 307 (1.2%) | 0 (0.0%) | 307 (1.2%) | 210 (3.6%) |
| Trm_Th1/Th17 | 1,585 (6.4%) | 905 (16.8%) | 1,585 (6.3%) | 330 (5.7%) |
| Trm_gut_CD8 | 64 (0.3%) | 0 (0.0%) | 64 (0.3%) | 0 (0.0%) |
| migDC | 70 (0.3%) | 0 (0.0%) | 70 (0.3%) | 141 (2.4%) |
| pDC | 61 (0.2%) | 0 (0.0%) | 61 (0.2%) | 0 (0.0%) |
| **Total** | 24,951 | 5,378 | 24,967 | 5,815 |

Table S4: Cell Type distribution for experiments using **Lung** tissue.

| Cell Type | Experiment 1 | | Experiment 2 | |
|---|---|---|---|---|
| | Ref | Query | Ref | Query |
| AP | 5033 (16.2%) | 3788 (20.7%) | 3788 (20.7%) | 5033 (16.2%) |
| BA_doublets | 50 (0.2%) | 20 (0.1%) | 20 (0.1%) | 50 (0.2%) |
| BAa | 457 (1.5%) | 25 (0.1%) | 25 (0.1%) | 457 (1.5%) |
| BAb | 1801 (5.8%) | 712 (3.9%) | 712 (3.9%) | 1801 (5.8%) |
| BL | 3959 (12.7%) | 1541 (8.4%) | 1541 (8.4%) | 3959 (12.7%) |
| F1 | 200 (0.6%) | 61 (0.3%) | 61 (0.3%) | 200 (0.6%) |
| F2 | 77 (0.2%) | 1610 (8.8%) | 1610 (8.8%) | 77 (0.2%) |
| F3 | 4397 (14.1%) | 1458 (8.0%) | 1458 (8.0%) | 4397 (14.1%) |
| F_doublets | 59 (0.2%) | 53 (0.3%) | 53 (0.3%) | 59 (0.2%) |
| HS_doublets | 829 (2.7%) | 134 (0.7%) | 134 (0.7%) | 829 (2.7%) |
| HSa | 1970 (6.3%) | 430 (2.4%) | 430 (2.4%) | 1970 (6.3%) |
| HSb | 5630 (18.1%) | 4611 (25.2%) | 4611 (25.2%) | 5630 (18.1%) |
| I1_Myeloid | 348 (1.1%) | 256 (1.4%) | 256 (1.4%) | 348 (1.1%) |
| I2_NK | 74 (0.2%) | 40 (0.2%) | 40 (0.2%) | 74 (0.2%) |
| I3_Tcell | 697 (2.2%) | 531 (2.9%) | 531 (2.9%) | 697 (2.2%) |
| I5_PlasmaCell | 72 (0.2%) | 123 (0.7%) | 123 (0.7%) | 72 (0.2%) |
| VL1_LE | 73 (0.2%) | 99 (0.5%) | 99 (0.5%) | 73 (0.2%) |
| VL2_VE | 3848 (12.4%) | 2313 (12.6%) | 2313 (12.6%) | 3848 (12.4%) |
| VL3_PE | 1528 (4.9%) | 483 (2.6%) | 483 (2.6%) | 1528 (4.9%) |
| **Total** | 31,102 | 18,288 | 18,288 | 31,102 |

Table S5: Cell Type distribution for experiments using **Breast** tissue.

| Cell Type | Experiment 1 | | Experiment 2 | |
|---|---|---|---|---|
| | Ref | Query | Ref | Query |
| Blood progenitors 1 | 52 (2.8%) | 149 (3.4%) | 149 (3.4%) | 52 (2.8%) |
| Blood progenitors 2 | 56 (3.0%) | 212 (4.8%) | 212 (4.8%) | 56 (3.0%) |
| Caudal epiblast | 80 (4.3%) | 338 (7.7%) | 338 (7.7%) | 80 (4.3%) |
| Def. endoderm | 79 (4.3%) | 156 (3.6%) | 156 (3.6%) | 79 (4.3%) |
| Epiblast | 283 (15.4%) | 641 (14.6%) | 641 (14.6%) | 283 (15.4%) |
| Gut | 56 (3.0%) | 108 (2.5%) | 108 (2.5%) | 56 (3.0%) |
| Haematoendothelial progenitors | 69 (3.7%) | 185 (4.2%) | 185 (4.2%) | 69 (3.7%) |
| Mesenchyme | 218 (11.8%) | 379 (8.6%) | 379 (8.6%) | 218 (11.8%) |
| Mixed mesoderm | 190 (10.3%) | 390 (8.9%) | 390 (8.9%) | 190 (10.3%) |
| Nascent mesoderm | 160 (8.7%) | 485 (11.0%) | 485 (11.0%) | 160 (8.7%) |
| Primitive Streak | 143 (7.8%) | 395 (9.0%) | 395 (9.0%) | 143 (7.8%) |
| Rostral neurectoderm | 300 (16.3%) | 736 (16.8%) | 736 (16.8%) | 300 (16.3%) |
| Surface ectoderm | 53 (2.9%) | 92 (2.1%) | 92 (2.1%) | 53 (2.9%) |
| Visceral endoderm | 104 (5.6%) | 128 (2.9%) | 128 (2.9%) | 104 (5.6%) |
| **Total** | 1,843 | 4,394 | 4,394 | 1,843 |

Table S6: Cell Type distribution for experiments using **Gastrula** tissue.

| Cell Type | Experiment 1 | | Experiment 2 | | Experiment 3 | | Experiment 4 | |
|---|---|---|---|---|---|---|---|---|
| | **Ref** | **Query** | **Ref** | **Query** | **Ref** | **Query** | **Ref** | **Query** |
| acinar | 844 (13%) | 947 (14%) | 114 (2%) | 948 (13%) | 108 (6%) | 3 (0%) | 829 (24%) | 2 (0%) |
| activated_stellate | 221 (3%) | 192 (3%) | 178 (4%) | 222 (3%) | 48 (3%) | 80 (5%) | 95 (3%) | 48 (4%) |
| alpha | 2069 (32%) | 1634 (24%) | 1186 (25%) | 2016 (28%) | 233 (12%) | 672 (39%) | 1117 (32%) | 283 (22%) |
| beta | 1635 (25%) | 2124 (32%) | 1729 (37%) | 2007 (28%) | 865 (45%) | 370 (22%) | 769 (22%) | 487 (38%) |
| delta | 386 (6%) | 472 (7%) | 439 (9%) | 498 (7%) | 213 (11%) | 125 (7%) | 160 (5%) | 101 (8%) |
| ductal | 927 (14%) | 739 (11%) | 508 (11%) | 769 (11%) | 114 (6%) | 293 (17%) | 351 (10%) | 270 (21%) |
| endothelial | 119 (2%) | 211 (3%) | 149 (3%) | 228 (3%) | 124 (7%) | 22 (1%) | 86 (2%) | 7 (1%) |
| epsilon | 5 (0%) | 16 (0%) | 15 (0%) | 15 (0%) | 13 (1%) | 1 (0%) | 2 (0%) | 1 (0%) |
| gamma | 182 (3%) | 166 (2%) | 218 (5%) | 191 (3%) | 69 (4%) | 84 (5%) | 35 (1%) | 63 (5%) |
| macrophage | 40 (1%) | 36 (1%) | 40 (1%) | 44 (1%) | 14 (1%) | 17 (1%) | 14 (0%) | 10 (1%) |
| mast | 17 (0%) | 16 (0%) | 18 (0%) | 24 (0%) | 8 (0%) | 9 (1%) | 7 (0%) | 1 (0%) |
| quiescent_stellate | 76 (1%) | 142 (2%) | 114 (2%) | 161 (2%) | 88 (5%) | 21 (1%) | 50 (1%) | 4 (0%) |
| schwann | 8 (0%) | 6 (0%) | 12 (0%) | 12 (0%) | 5 (0%) | 6 (0%) | 1 (0%) | 1 (0%) |
| t_cell | 5 (0%) | 5 (0%) | 5 (0%) | 6 (0%) | 1 (0%) | 2 (0%) | 2 (0%) | 1 (0%) |
| **Total** | 6,534 | 6,706 | 4,725 | 7,141 | 1,903 | 1,705 | 3,518 | 1,279 |

Table S7: Cell Type distribution for experiments using **Pancreas** tissue.

| Tissue | Experiment | Set of cells simulating OOD |
|---|---|---|
| Lung | Healthy | Naive B cells, Cycling, Memory B cells, Cycling T&NK |
| | Lung tumoral | MNP/T doublets, Naive B cells |
| Breast | 1 | VL1_LE, I2_NK, I5_PlasmaCell, BA_doublets |
| | 2 | F1, VL1_LE, I5_PlasmaCell |
| Gastrula | 1 | Haematoendothelial progenitors, Blood progenitors 1, Surface ectoderm, Gut, Blood progenitors 2 |
| | 2 | Surface ectoderm, Gut, Visceral endoderm |
| Pancreas | 1 | quiescent_stellate, macrophage, mast, schwann, epsilon, t_cell |
| | 2 | macrophage, mast, schwann, epsilon, t_cell |
| | 3 | quiescent_stellate, gamma, macrophage, mast, schwann, epsilon, t_cell |
| | 4 | quiescent_stellate , gamma, macrophage, mast, schwann, epsilon, t_cell |

Table S8: Cell types held out to simulate OOD conditions across the experimental scenarios designed for the evaluation of the annotator. For each tissue and experiment, the specified cell populations were omitted from the reference set and treated as OOD in the query.

# S5 Anomaly detector experiments

In this section, we further extend the discussion on the anomaly detector and provide additional results.

## S5.1 Expanded discussion about the FDR role in our anomaly detection problem

The performance of an anomaly detector is typically assessed using *Statistical Power* and the *False Discovery Rate* (FDR) scores. Statistical power, usually known as *Recall*, measures the proportion of actual anomalies, *i.e.*, OOD cells, correctly identified by the detector among all OOD cells. FDR, on the other hand, measures the expected proportion of false positives among all detected anomalies. It is defined as $FP/(FP + TP)$, where FP denotes false positives and TP denotes true positives –here, a positive indicates an OOD sample is detected correctly. Although FDR is commonly employed to assess the performance of the anomaly detector, it may not be suitable in our scenario. Specifically, when the number of ID cells vastly exceeds the number of OOD cells, the FDR can be high even for a well-performing detector. This is just because if $FP \gg TP$, then $(FP + TP) \approx FP$, even if $FP \ll (FP + TP + FN + TN)$.

To illustrate this point, let us consider the following scenario: we have a query of $2 \cdot 10^5$ cells, among which only 200 cells are OOD. The detector correctly identifies 180 of these OOD cells, achieving a power (recall) of 90%, but it also incorrectly labels 400 ID cells as OOD, the resulting FDR would be 68.96%. This high FDR might suggest poor performance. However, those 400 false positives represent only 0.2% of the total ID cell population. Hence, the detector effectively identified the majority of true anomalies at the cost of misclassifying a very small fraction (0.2%) of ID samples. This interpretation must be handled with caution, as the impact of false positives varies significantly depending on whether the misflagged cells belong to an over- or under-represented cell type, a distinction that is generally challenging to discern *a priori*.

## S5.2 Results and extended discussion of the anomaly detector

Here, we report additional results of our anomaly detector under the leave-one-cell-type-out evaluation scheme, *i.e.*, we remove all samples that belong to a specific cell type from the reference set, and then assess whether the model correctly flags those cells as OOD in the query set. We evaluate the performance scores across various values of $\alpha_0$ and different train/-calibration configurations. All evaluated reference–query pairs were preprocessed using the TorchNet pipeline. Figures S3 and S4 show the results for healthy-immune and tumoral-immune lung query cells, respectively. We observe that the healthy-cell experiments follow the statistical guarantees described by Equations 9 and 10 regarding the FPR, whereas the tumoral samples exhibited evident deviations, as expected.

Likewise, Figures S5, S6, and S7 show the performance on pancreas, gastrula, and breast tissues, where the theoretical FPR guarantees are again achieved. Across all tissues, we observed that the FDR tend to be larger when the fraction of OOD cells is large, even if

the performance of the anomaly detector is strong. Finally, we note that the detector is not infallible: in a few cases, some OOD cell types were never detected. These failures may occur if the biological distance with another cell present in the data is very close.

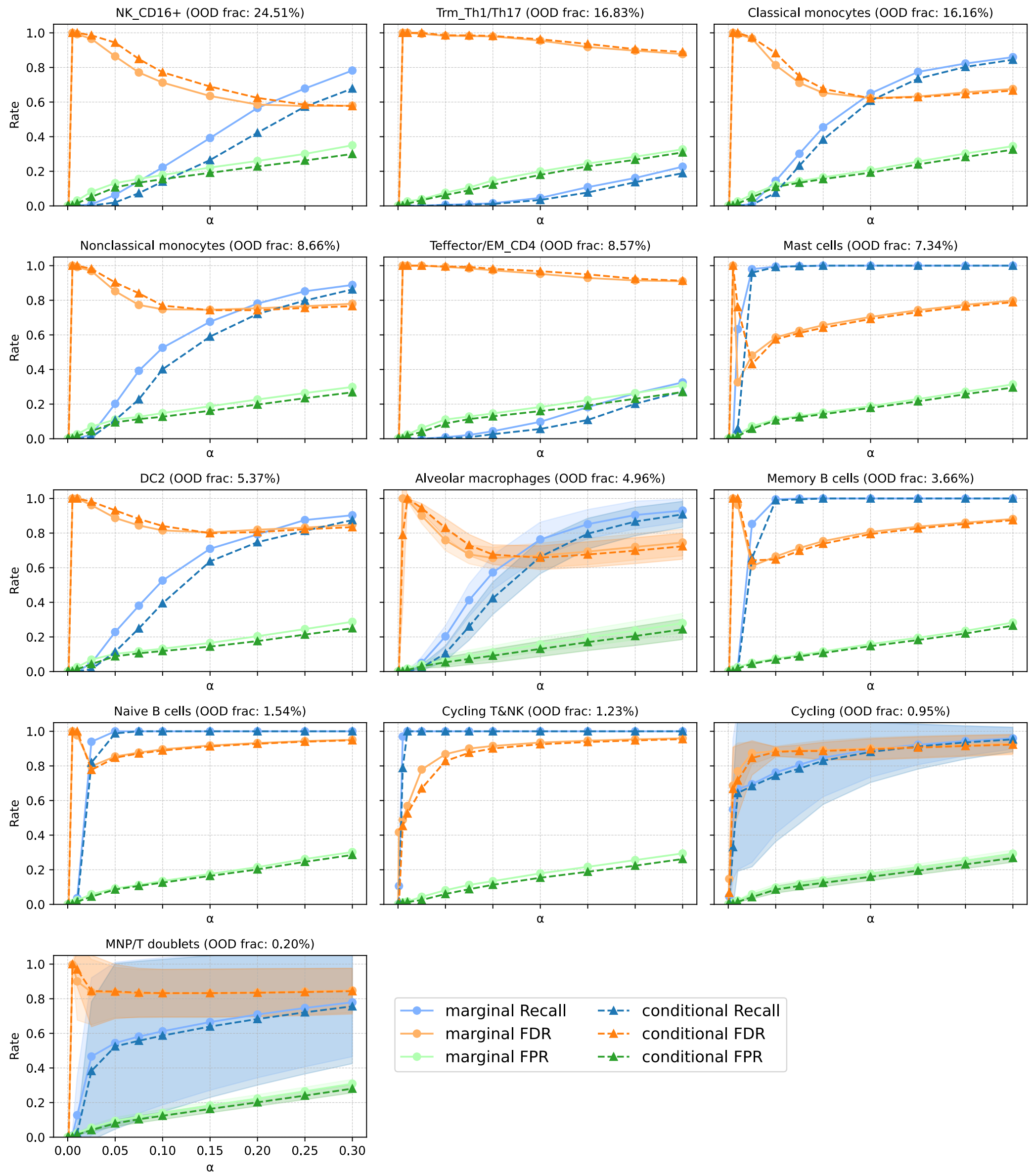


Figure S3: Performance of the anomaly detector on a **Lung healthy immune** cell query. The detector was evaluated using a leave-one-cell-type-out scheme. In each fold of the evaluation, one cell type is held out from the reference and designated as the true OOD target in the query. Metrics reported include statistical Power (blue), FDR (orange), and FPR (green). The fraction of true OOD cells among all the query cells is also reported. The x-axis $\alpha_0$ values range from $10^{-3}$ to 0.3.

### S5.3 Testing the automatic $\alpha_o$ selector

We present a comprehensive set of experiments designed to show the reader how the automatic $\alpha_o$ selector, described in Algorithm 1, is expected to perform across a different range of batch effects and OOD concentrations.

We conducted a series of experiments using the two immune-lung query datasets: the healthy-lung dataset, which is expected to be largely free of batch effects as reported in the Section 2.4 of the main manuscript, and the diseased-lung dataset, characterized by expected transcriptomic alterations due to the known biological batch. In these experiments, we compare the empirical coverage obtained by the annotator using the automatic $\alpha_o \in [0, 1]$ selection with a baseline that fixes $\alpha_o = 0$. To systematically assess performance under varying degrees of batch effects, we introduced OOD cells, *i.e.* absent from the training and calibration sets, at several proportions, see Table S9. Each experiment was repeated 20 times with different random data partitions to ensure robust results. The outcomes of these experiments are reported in Figures S8, S9, S10, and S11.

| **Experiment** | **Healthy % OOD cells** | **Ill % OOD cells** | **Cells excluded** |
|---|---|---|---|
| 1 | 0% | 0% | - |
| 2 | 5.4% | 5.1% | *DC2* |
| 3 | 11% | 13.2% | *Naive B cells*, *Cycling*, *Naive B cells*, *Cycling*, *Memory B cells* and *Cycling T&NK* |
| 4 | 25.6% | 24.1% | *NK_CD16+*, *NK_CD56bright_CD16-*, *intermediate macrophages* and *Tregs* |

Table S9: Experiments to assess the empirical coverage behavior when significance threshold $\alpha_o$ is automatically selected in lung healthy and tumoral queries, respectively. We present the corresponding percentage of samples that cells excluded from the training data represents in each of the query sets, as well as the names of the excluded cell types.

Figure S8 illustrates the scenario without OOD cells. For those experiments where $\alpha_o = 0$ is fixed, the results of the experiments using the tumoral query data (orange) show a substantial deviation of the empirical coverage versus the theoretical value. This fact is consistent with the broken exchangeability due to the known batch effect present between the healthy reference and the tumoral dataset. Notably, for the tumoral data our automatic procedure selects an $\alpha_o$ that brings the empirical coverage to the theoretical target by effectively filtering batch-affected observations. This correction, however, requires a relatively large $\alpha_o$, leading to the exclusion of many query samples. For the healthy data (green), the procedure identifies batch effects (*i.e.*, $\alpha_o$ does not halt immediately) and yields modest improvements in empirical coverage, which seems to achieve the theoretical value in expectation. This likely reflects residual batch effects arising from differences in the source of the reference and query sets, a common situation in single-cell studies. Finally, the power for OOD detection remains zero in both cases, as no OOD cells are present in these experiments.

Figures S9 and S10 show a strong performance of the automatic $\alpha_o$ detector. The recall

scores indicate that nearly all OOD samples are detected. Compared with the baseline, *i.e.*, the same experiments with $\alpha_o = 0$, the experiments with the automatic selector produce an empirical coverage in agreement with the theoretical value. Figure S11, which includes a larger fraction of OOD cells, further illustrates how the procedure detects, on average, 80% of the OOD cells, thereby improving empirical coverage relative to the baseline.

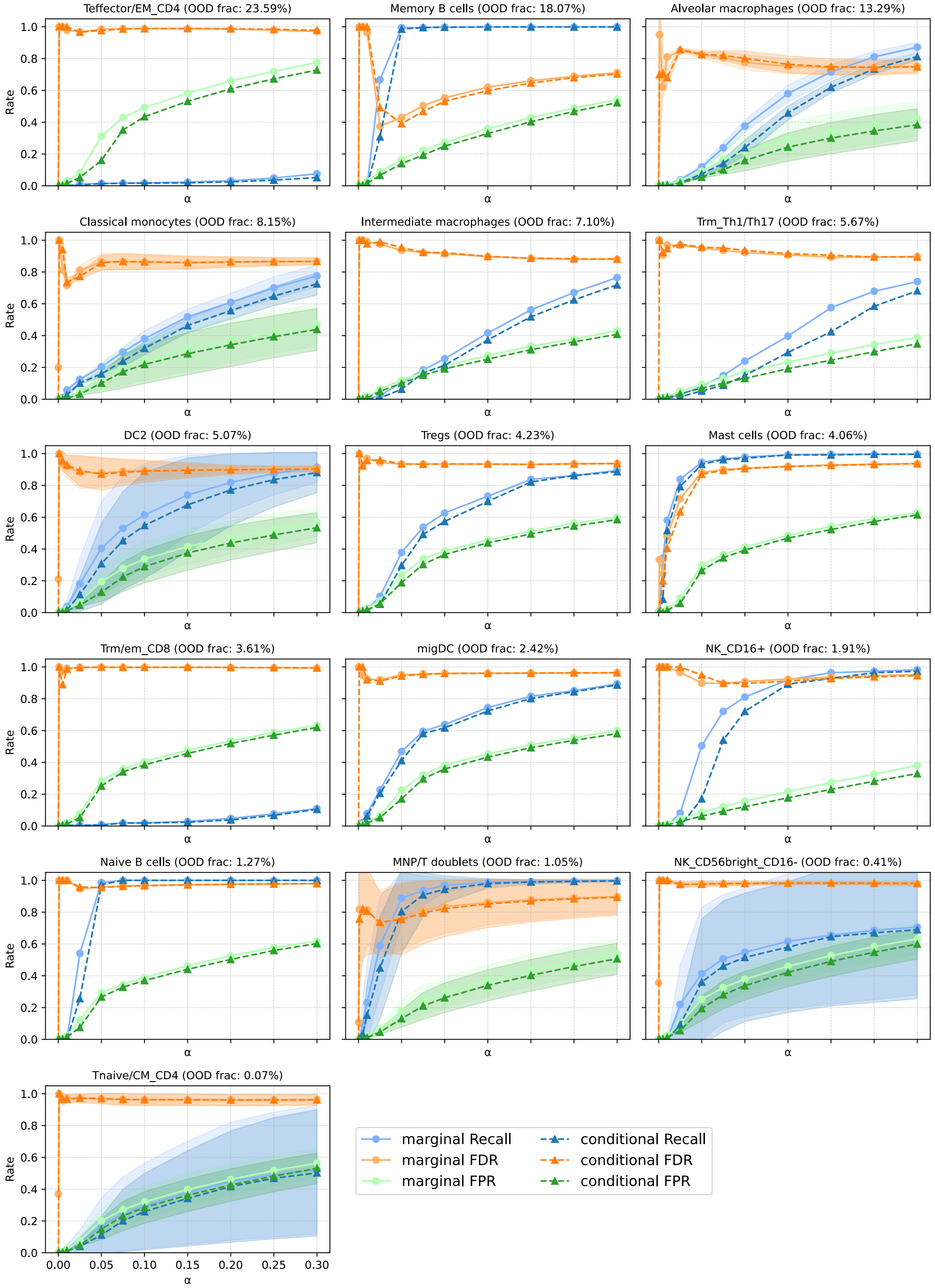


Figure S4: Performance of the anomaly detection model for **Lung tumoral immune query** cells. The detector was evaluated using a leave-one-cell-type-out scheme. In each fold of the evaluation, one cell type is held out from the reference and designated as the true OOD target in the query. Metrics reported include statistical Power (blue), FDR (orange), and FPR (green). The fraction of true OOD cells among all the query cells is also reported. The $\alpha_0$ values range from $10^{-3}$ to 0.3.

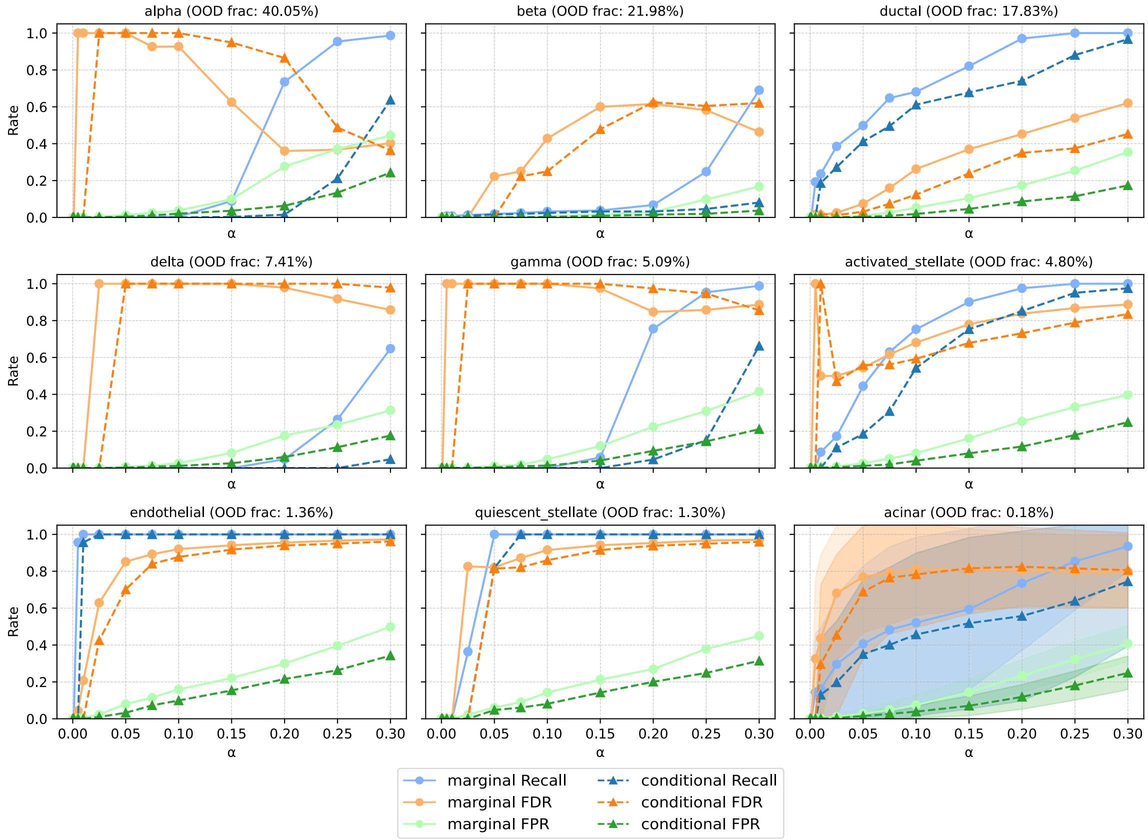


Figure S5: Performance of the anomaly detection model for **Pancreas** experiments. The detector was evaluated using a leave-one-cell-type-out scheme. In each fold of the evaluation, one cell type is held out from the reference and designated as the true OOD target in the query. Metrics reported include statistical Power (blue), FDR (orange), and FPR (green). The fraction of true OOD cells among all the query cells is also reported. The $\alpha_0$ values range from $10^{-3}$ to 0.3.

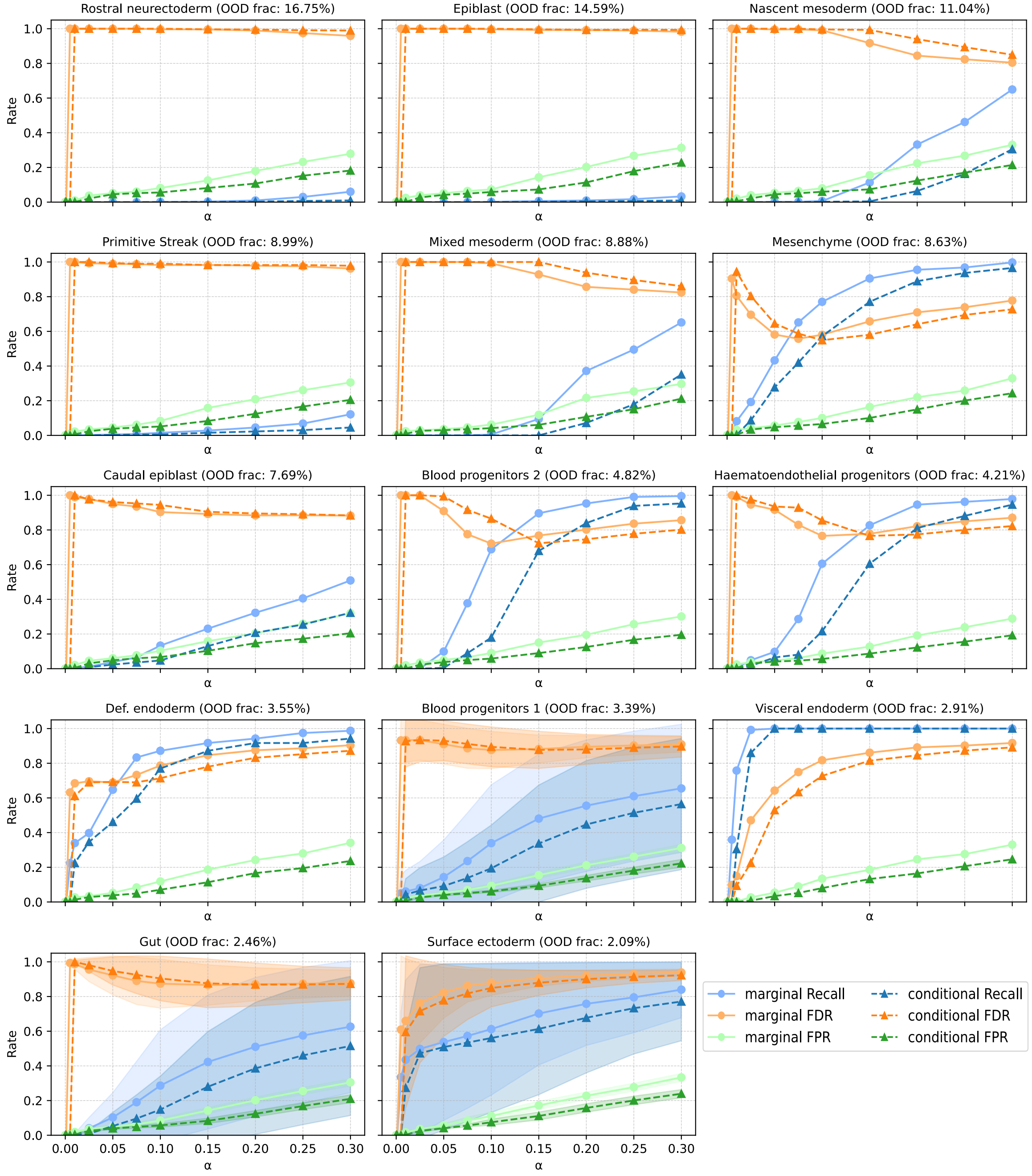


Figure S6: Performance of the anomaly detection model for **Gastrulation** experiments. The detector was evaluated using a leave-one-cell-type-out scheme. In each fold of the evaluation, one cell type is held out from the reference and designated as the true OOD target in the query. Metrics reported include statistical Power (blue), FDR (orange), and FPR (green). The fraction of true OOD cells among all the query cells is also reported. The $\alpha_0$ values range from $10^{-3}$ to 0.3.

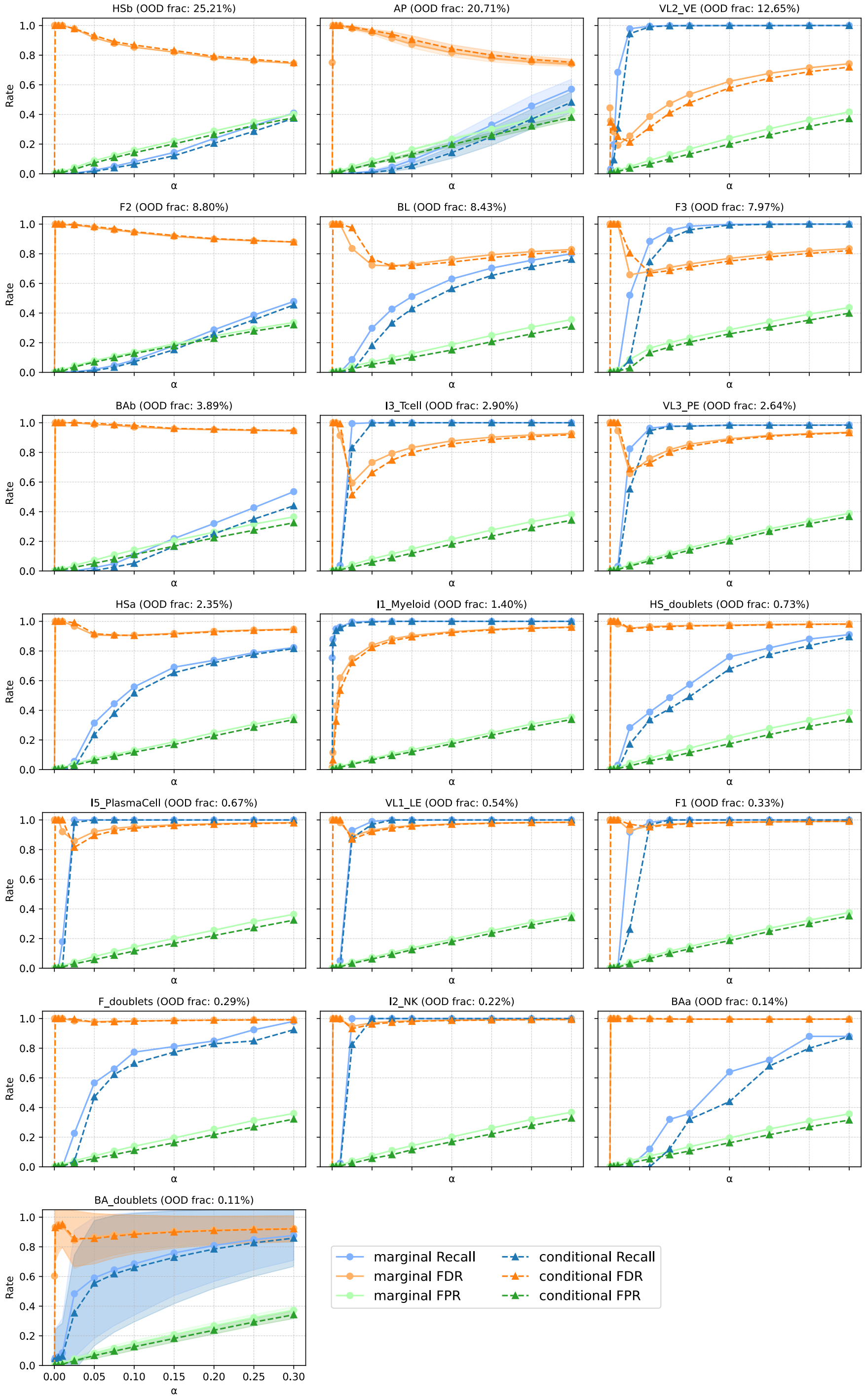


Figure S7: Performance of the anomaly detection model for **Breast** experiments. The detector was evaluated using a leave-one-cell-type-out scheme. In each fold of the evaluation, one cell type is held out from the reference and designated as the true OOD target in the query. Metrics reported include statistical Power (blue), FDR (orange), and FPR (green). The fraction of true OOD cells among all the query cells is also reported. The $\alpha_0$ values range from $10^{-3}$ to 0.3.

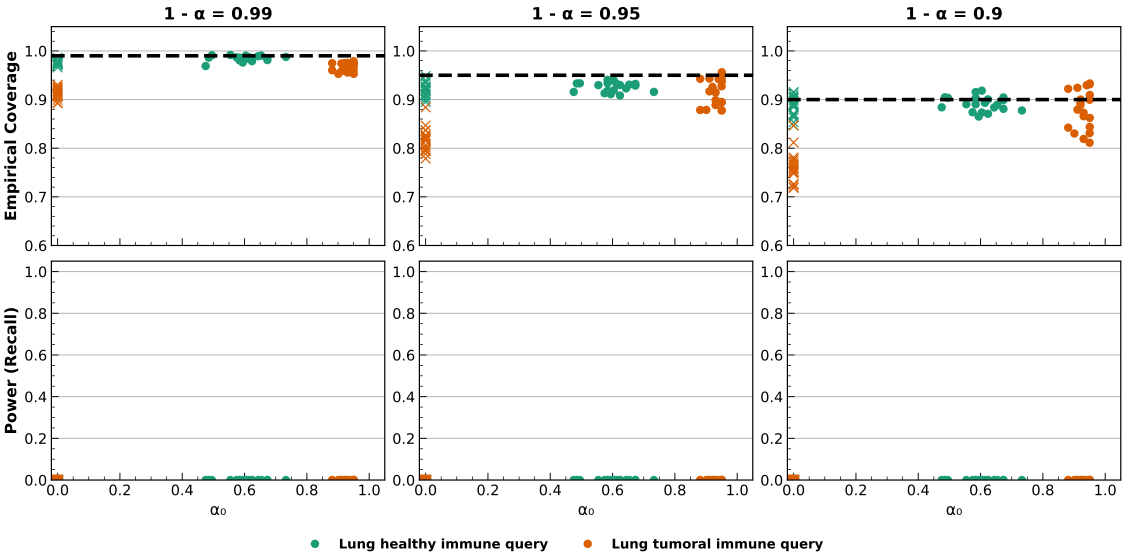


Figure S8: Results of experiment 1 from Table S9. Each point represents a single experimental realization. The top panel displays the empirical coverage achieved, with the theoretical value indicated by the dashed horizontal line. The bottom panel shows the corresponding performance of the anomaly detector in identifying OOD samples. Each point in the upper panel is uniquely associated with a point in the lower panel, representing the same experimental instance (They have the same $\alpha_o$ value).

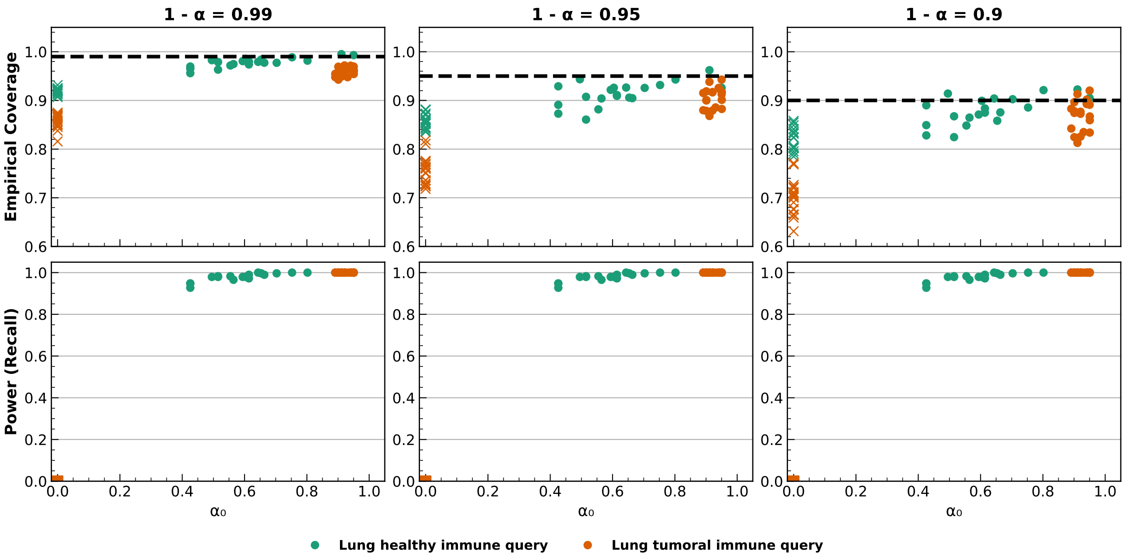


Figure S9: Results of experiment 2 from Table S9. Each point represents a single experimental realization. The top panel displays the empirical coverage achieved, with the theoretical value indicated by the dashed horizontal line. The bottom panel shows the corresponding performance of the anomaly detector in identifying OOD samples. Each point in the upper panel is uniquely associated with a point in the lower panel, representing the same experimental instance (They have the same $\alpha_o$ value).

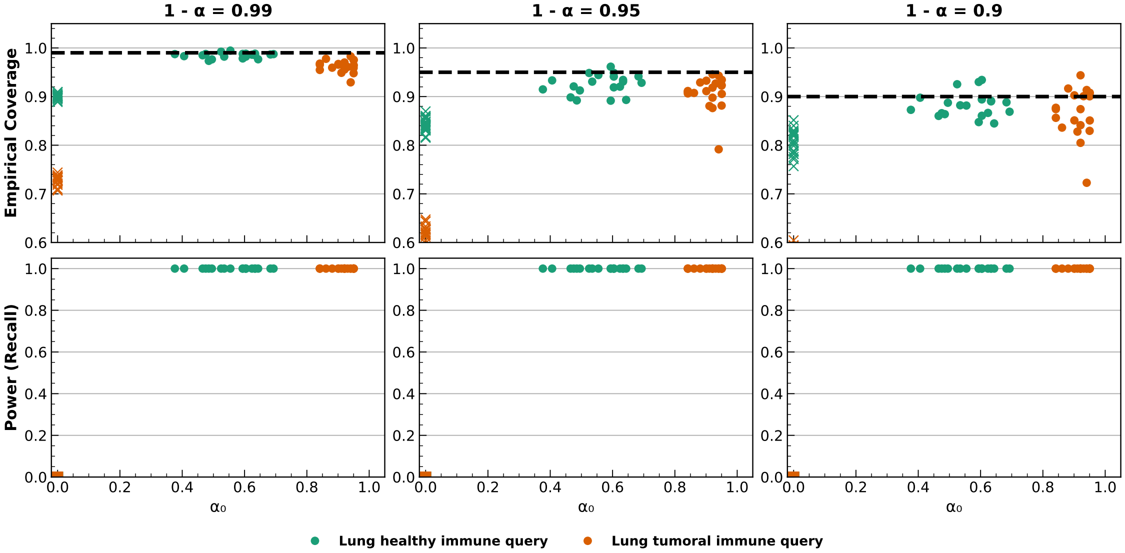


Figure S10: Results of experiment 3 from Table S9. Each point represents a single experimental realization. The top panel displays the empirical coverage achieved, with the theoretical value indicated by the dashed horizontal line. The bottom panel shows the corresponding performance of the anomaly detector in identifying OOD samples. Each point in the upper panel is uniquely associated with a point in the lower panel, representing the same experimental instance (They have the same $\alpha_o$ value).

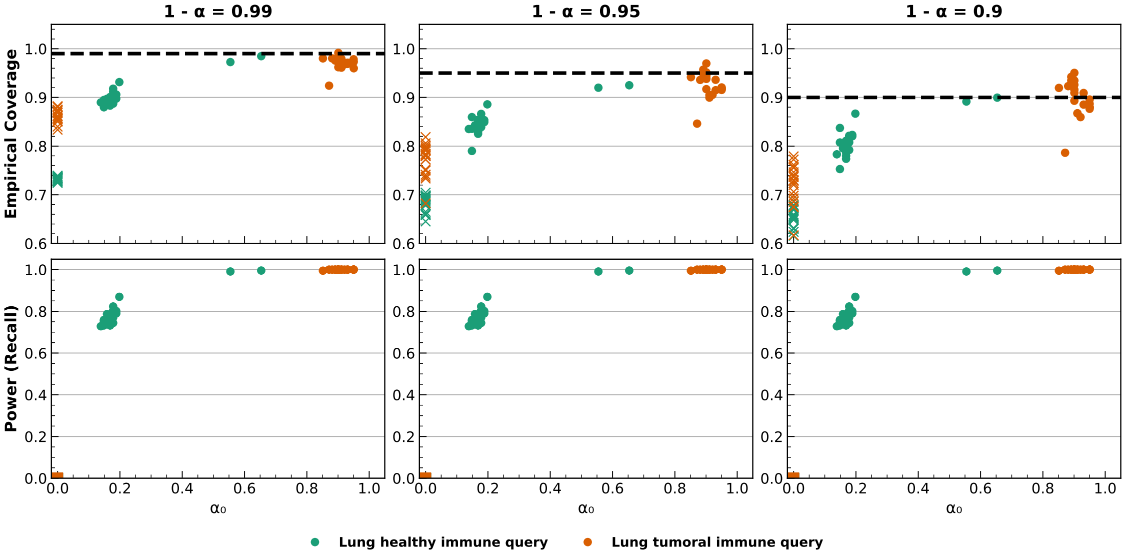


Figure S11: Results of experiment 4 from Table S9.Each point represents a single experimental realization. The top panel displays the empirical coverage achieved, with the theoretical value indicated by the dashed horizontal line. The bottom panel shows the corresponding performance of the anomaly detector in identifying OOD samples. Each point in the upper panel is uniquely associated with a point in the lower panel, representing the same experimental instance (They have the same $\alpha_o$ value).

## S6 Additional supporting results

Ensuring the statistical assumption of exchangeability in single-cell data between reference and query sets is challenging due to the inherent structure of the data and likely technical variance. With this in consideration, we present here the calibration curves achieved by our annotator for the experiments designed in Table S8. The calibration curves shown in Figure S12, reveal that, for the healthy lung tissue query in panel a), the empirical coverage appears well-calibrated, showing only slight under-coverage with the classwise taxonomy. These deviations are probably due to remaining small batch effects. In contrast, the tumor tissue query shown in panel b) exhibits a systematic deviation, as expected due to the presence of a known biological batch (tumor cells). Figures S13, S14, and S15 exhibit well-calibrated patterns, with the query results aligning closely to the theoretical behavior. The internal-test calibration curves are perfectly calibrated. This happens because they are derived from a held-out portion of the reference data that was never seen during training, so by design of the experiment, they are exchangeable with no hidden batch effects.

As discussed in the manuscript, classwise taxonomy computes a separate calibration for each class. While this label-conditional approach ensures accurate per-class coverage, it significantly reduces calibration sample sizes per class, leading to instability in coverage for minority classes. Moreover, when many classes have small sample sizes, the average coverage gap (CovGap) can increase because the metric assigns equal weight to each class regardless of its frequency. This is a common scenario in real-world applications with many classes or imbalanced datasets [25]. Figures S16, S17, S18, and S19 show the CovGap score for standard and classwise taxonomies in all the experiments. Figure S20 shows an illustrative example of the coverage results stratified by cell types in the Gastrula tissue experiments.

We evaluated the annotator's performance using three different underlying models: TorchNet, Scmap, and CellTypist. Results of these evaluations are reported in Figure 3 of the main manuscript and in Supplementary Figures S21 and S22. Although the conformal guarantees are agnostic to the underlying classifier, we observed empirical differences in the results. These differences likely arise from the different preprocessing pipelines used. Specifically, CellTypist requires either log-normalized or raw input, whereas Scmap follows the "cell" configuration, so the data for the experiments with this model also uses the same log-normalized data, *i.e.*, none of them were Harmony-integrated. These differences only affect the prediction stage. The anomaly detector was consistently trained on identical batch-corrected data, yielding uniform performance across experiments. We observed in Figure S21 that Scmap achieved strong performance results under the classwise and cluster taxonomies, but failed with the standard taxonomy. This is likely due to the Scmap's cluster-based methodology, which aligns naturally with the calibration of the classwise and cluster taxonomies. In contrast, the standard taxonomy returned a substantial number of empty prediction sets, which are considered fails, degrading its performance. By comparison, TorchNet delivered near-theoretical coverage across all taxonomies, with only minor deviations under the standard taxonomy (see Figure 3 of the main manuscript). Finally, experiments using CellTypist in Figure S22 showed a clear deviation between theoretical and empirical coverage, likely due to preprocessing requirements specific to this annotator- log-normalized or raw counts. We conducted these illustrative experiments to show the reader that our methodology can be used with any underlying model.

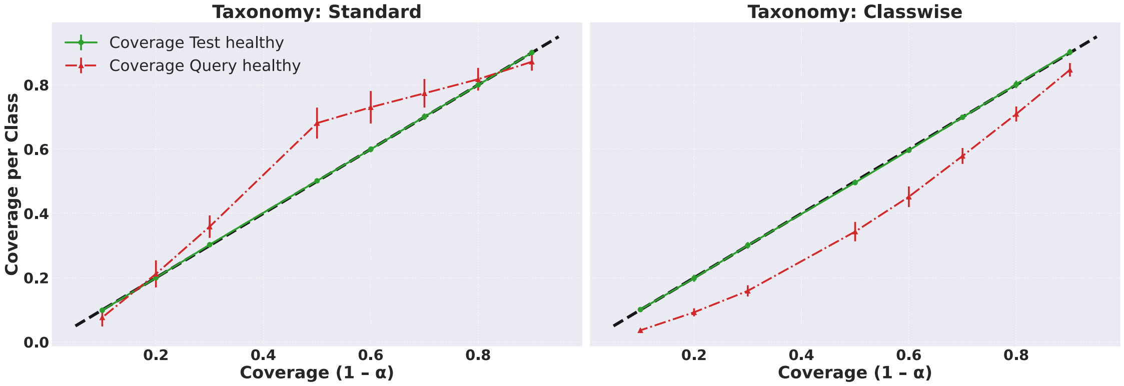


(a) Calibration performance for **healthy** Lung tissue query.

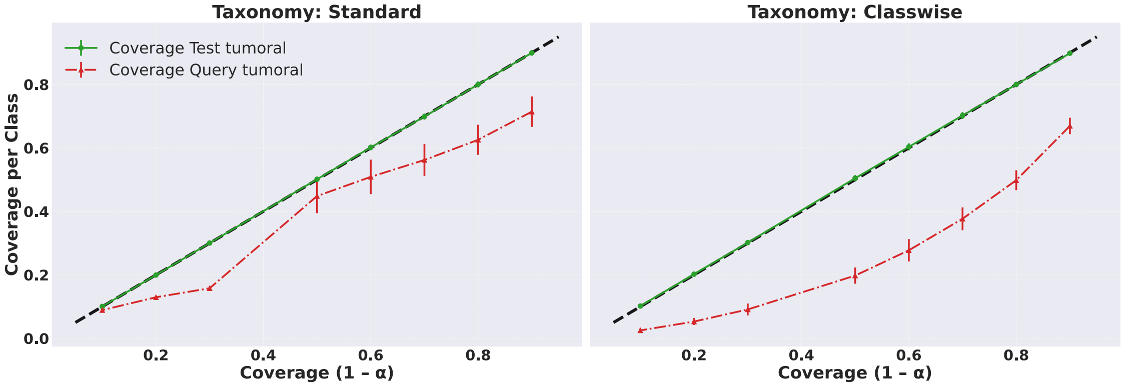


(b) Calibration performance for **tumor** Lung tissue query

Figure S12: Calibration curves of the annotation model, using TorchNet as underlying model, applied to immune **Lung** cell experiments. Each panel shows both the query-based predictions and the internal test coverage. Panel (a) presents results for healthy lung tissue query; Panel (b) shows the results for lung tissue query obtained from tumor samples. Black diagonal line represents a perfect calibrated result.

We evaluated TorchNet as the underlying annotator across the remaining tissues. Results are illustrated in Supplementary Figures S23, S24, S25, and S26, corresponding to healthy lung immune tissue, lung immune tissue from patients with lung tumors, gastrula, and pancreatic tissues, respectively. In these experiments, specific cell types listed in Supplementary Table S8 were excluded from the reference but retained in the query samples to simulate OOD cells. As exposed in the main manuscript (see Section 2.4), the same reference dataset was used for healthy lung samples and lung tumor patient samples. Despite sharing a common origin (lung immune cells), samples from patients with lung tumors reported a large divergence between theoretical and empirical coverage, along with notable deviations in the CovGap metric, due to the biological batch effects. For gastrula and pancreatic tissues, theoretical coverage was nearly achieved by the empirical, with minimal deviations likely due to slight leakage of OOD cells from the anomaly detector. Finally, we observed that the

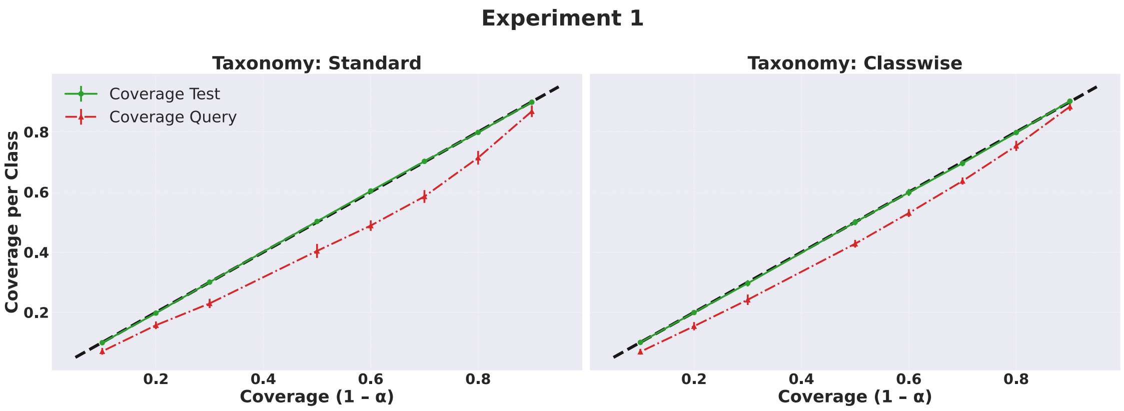


(a) Calibration performance for Breast Experiment 1.

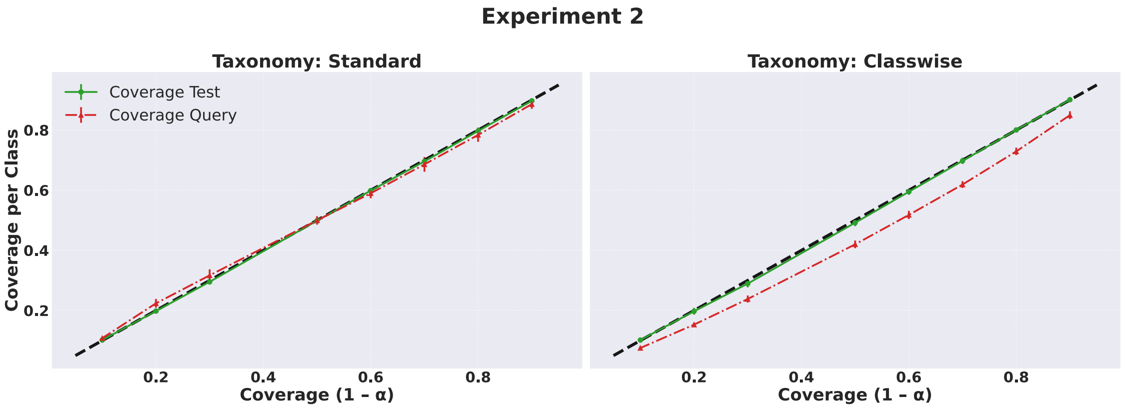


(b) Calibration performance for Breast Experiment 2.

Figure S13: Calibration curves of the annotation model, using TorchNet as underlying model, applied to **breast** cell experiments. Each panel shows both the query-based predictions and the internal test coverage. Panel (a) presents results for the first experiment on this tissue, whereas Panel (b) includes the second sub-cohort. Black diagonal line represents perfect calibrated results.

stability of cluster taxonomy becomes unstable under very strict coverage requirements, *e.g.* $1 - \alpha \geq 0.99$, often returning uninformative prediction sets.

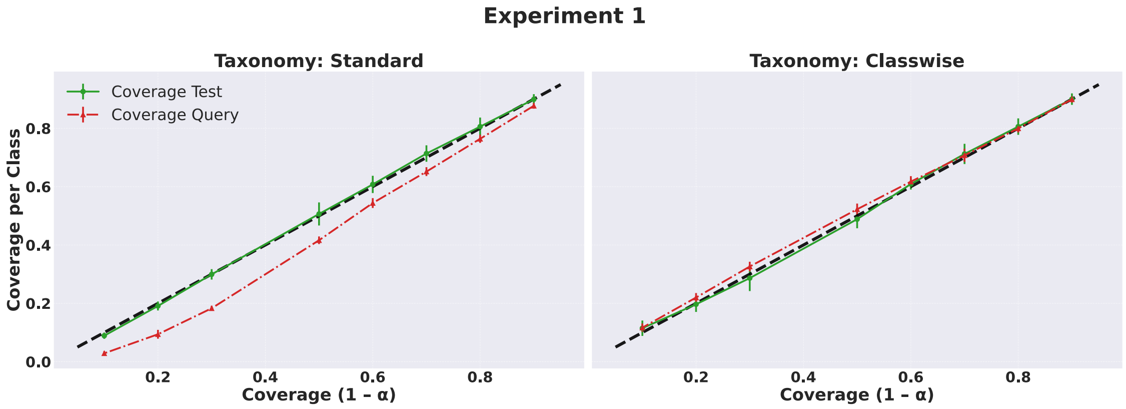


(a) Calibration performance for Gastrula Experiment 1.

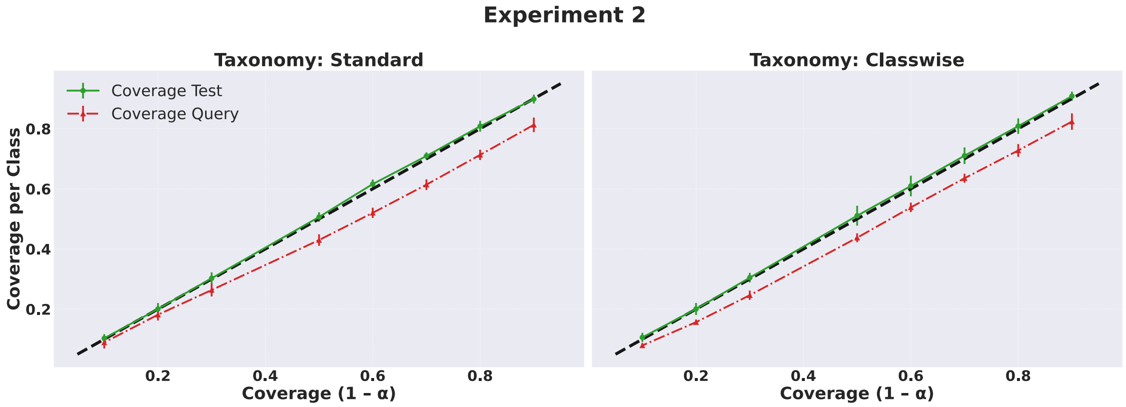


(b) Calibration performance for Gastrula Experiment 2.

Figure S14: Calibration curves of the annotation model, using TorchNet as underlying model, applied to **Gastrula** cell experiments. Each panel shows both the query-based predictions and the internal test coverage. Panel (a) presents results for the first experiment on this tissue, whereas Panel (b) includes the second sub-cohort. Black diagonal line represents perfect calibrated results.

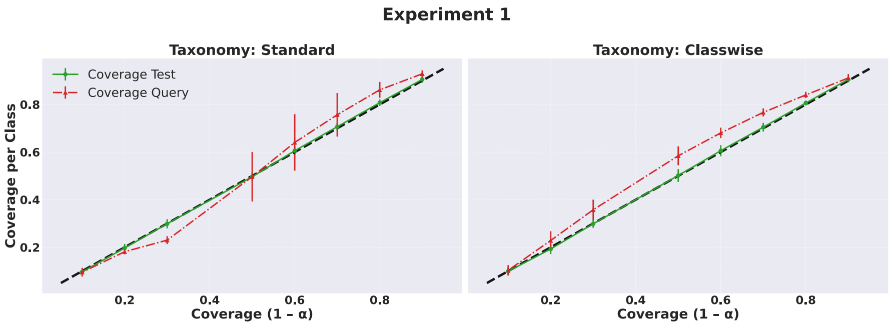


(a) Calibration performance for Pancreas Experiment 1.

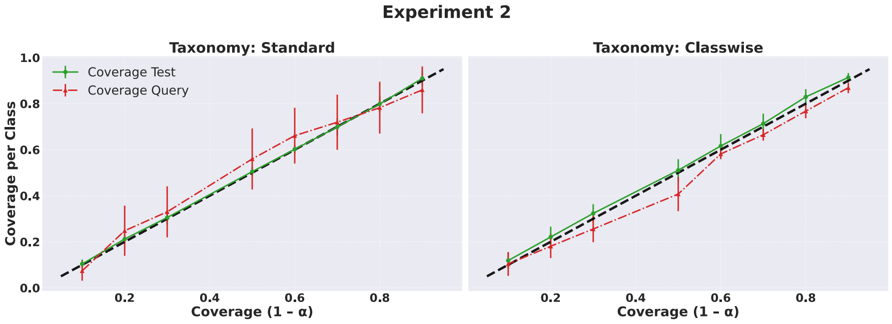


(b) Calibration performance for Pancreas Experiment 2.

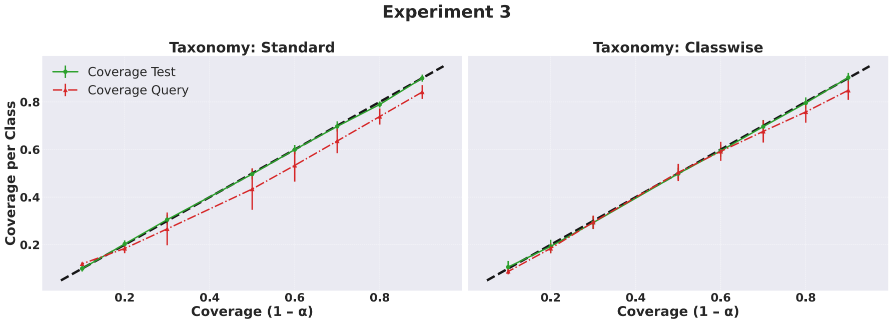


(c) Calibration performance for Pancreas Experiment 3

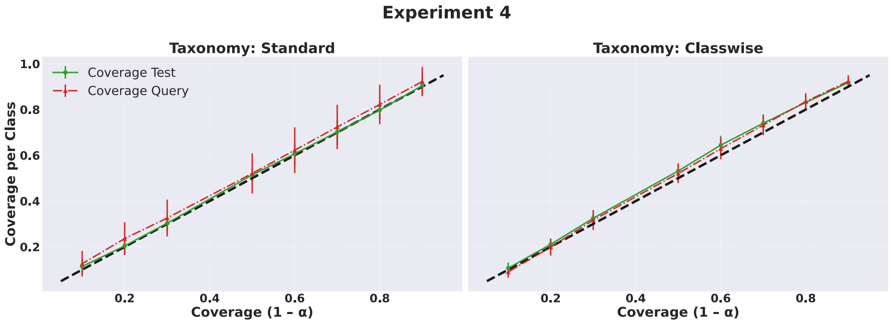


(d) Calibration performance for Pancreas Experiment 4

Figure S15: Calibration curves of the annotation model, using TorchNet as underlying model, applied to **Pancreas** cell experiments. Each panel shows both the query-based predictions and the internal test coverage. Figures (a),(b),(c), and (d) represents experiments 1,2,3, and 4, respectively. Black diagonal line represents perfect calibrated results.

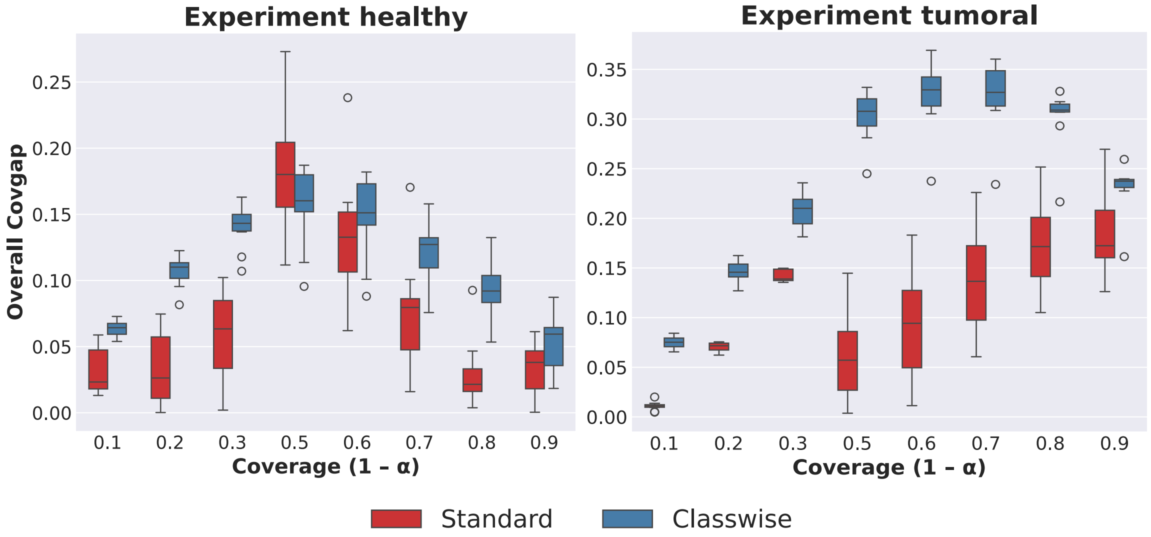


Figure S16: Results of the CovGap score for the standard and classwise taxonomies in the experiments for the **Lung** tissue, measured at different coverages levels $1-\alpha$.

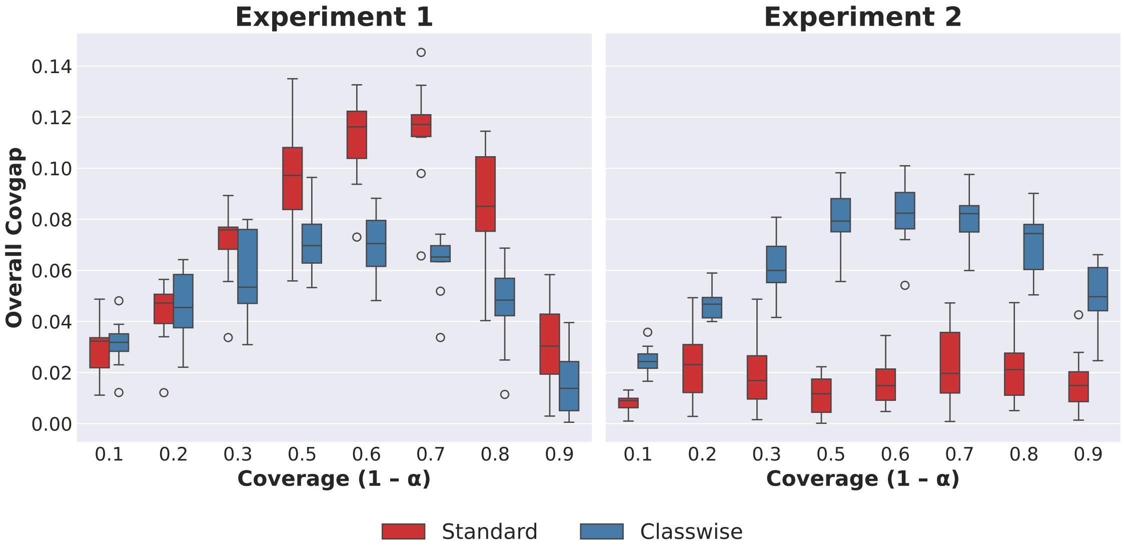


Figure S17: Results of the CovGap score for the standard and classwise taxonomies in the experiments for the **Breast** tissue, measured at different coverages levels $1-\alpha$.

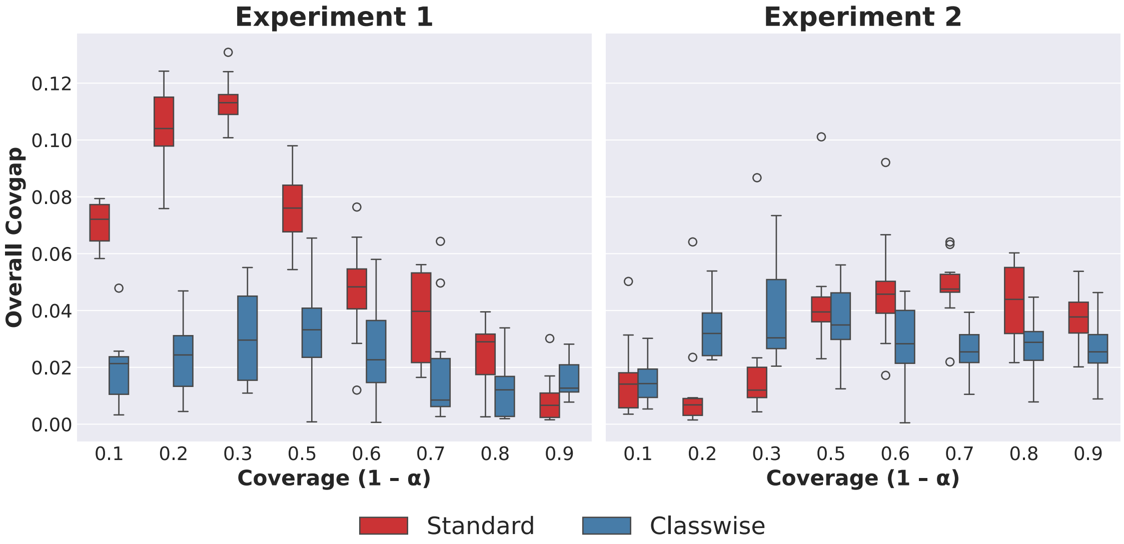


Figure S18: Results of the CovGap score for the standard and classwise taxonomies in the experiments for the **Gastrula** tissue, measured at different coverages levels $1 - \alpha$. CovGap score performs better for classwise taxonomy as expected, except for high coverage levels where both taxonomies perform similarly. The rationale is that high coverage levels usually implies larger prediction sets.

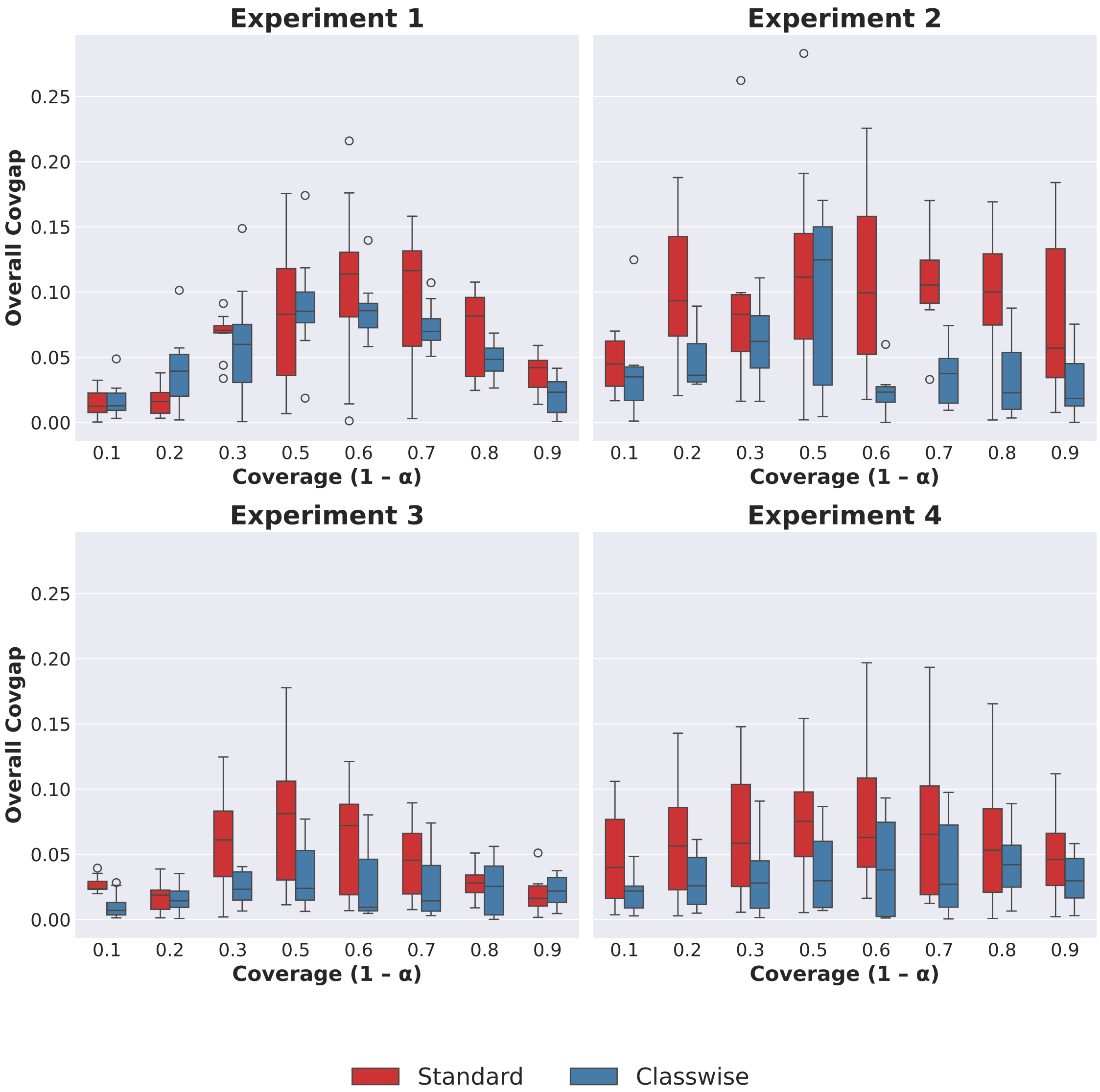


Figure S19: Results of the CovGap score for the standard and classwise taxonomies in the experiments for the **Pancreas** tissue, measured at different coverages levels $1 - \alpha$.

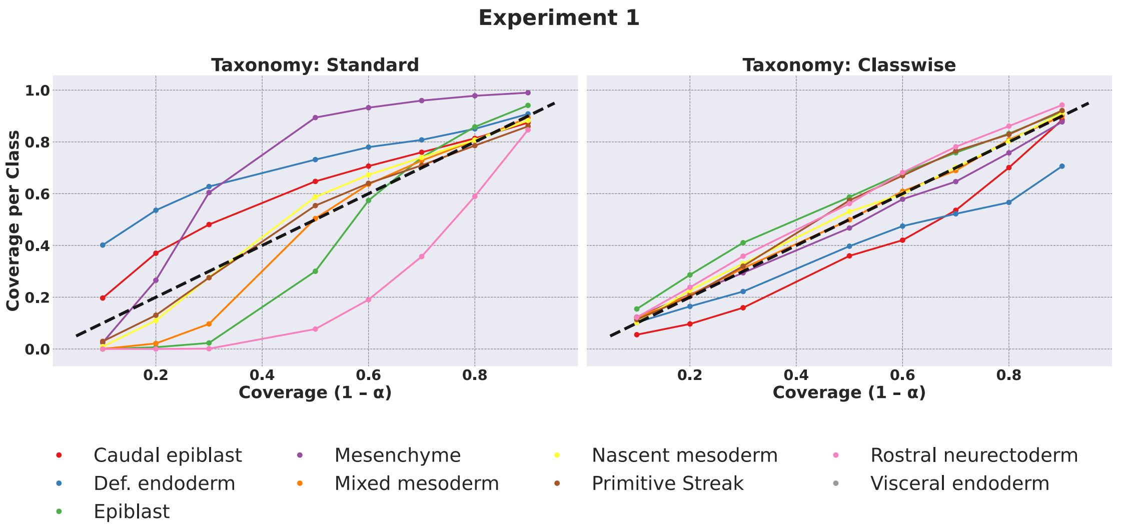


(a) Results for experiment 1.

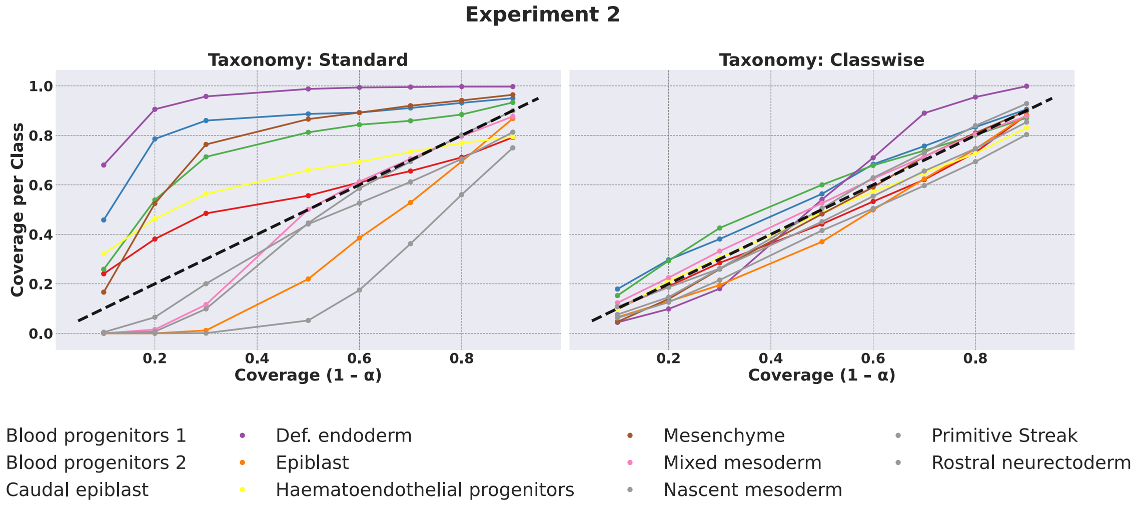


(b) Results for experiment 2.

Figure S20: Illustrative example of the coverage results stratified by cell types in experiments with **Gastrula** tissue. We illustrate the differences in coverage deviation between a standard conformal predictor—which employs a single, global nonconformity threshold—and the class-wise conformal predictor that calibrates thresholds separately for each class.

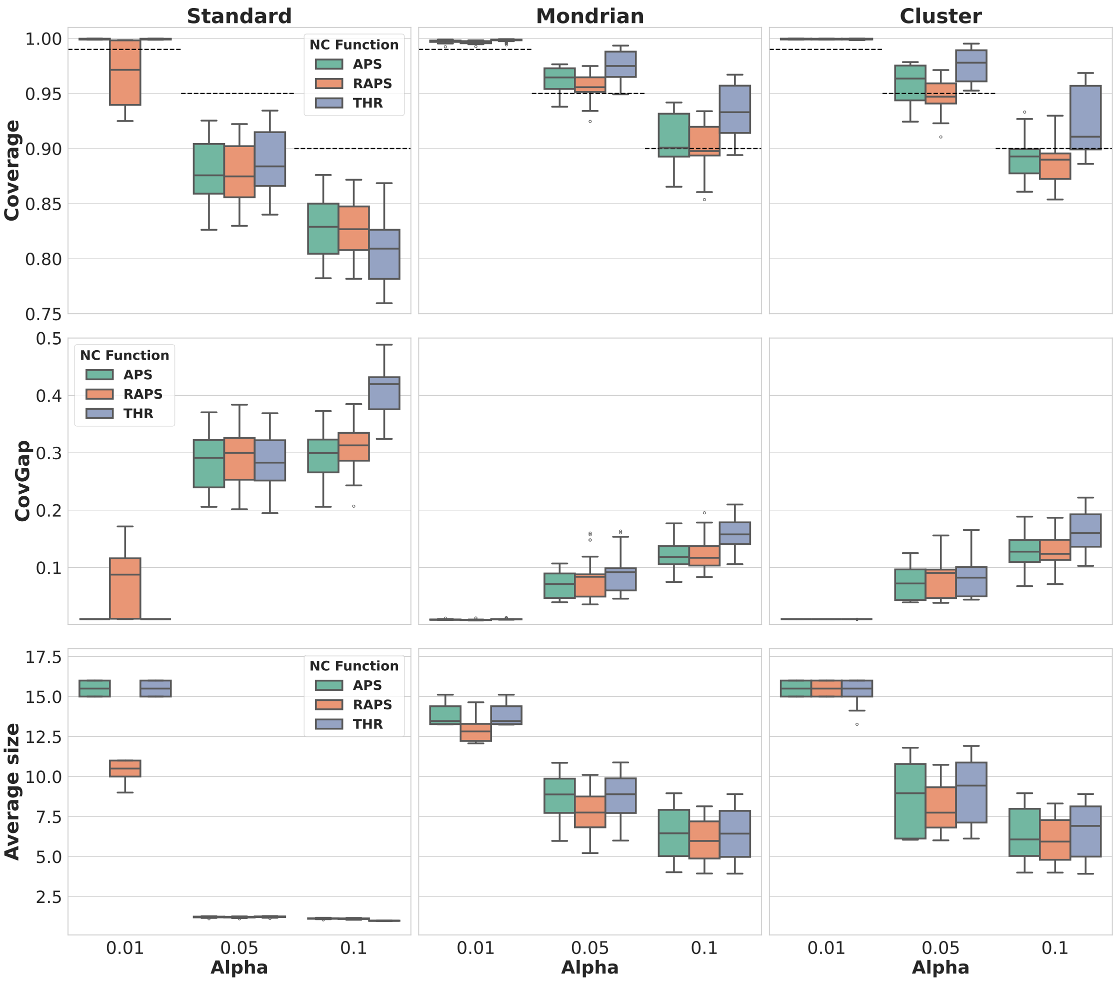


Figure S21: Averaged results for the **breast** tissue query data using **SCmap** as underlying model. The columns represent the three taxonomies compared: standard, classwise and cluster. Each row displays the results for one of the three measured performance scores: coverage, covgap, and set size. Within each chart, every column corresponds to a nominal error level $\alpha = 0.01, 0.05, 0.10$, and the different non-conformity functions: $s_{\mathsf{APS}}$, $s_{\mathsf{RAPS}}$ and $s_{\mathsf{THR}}$, are indicated by distinct colors.

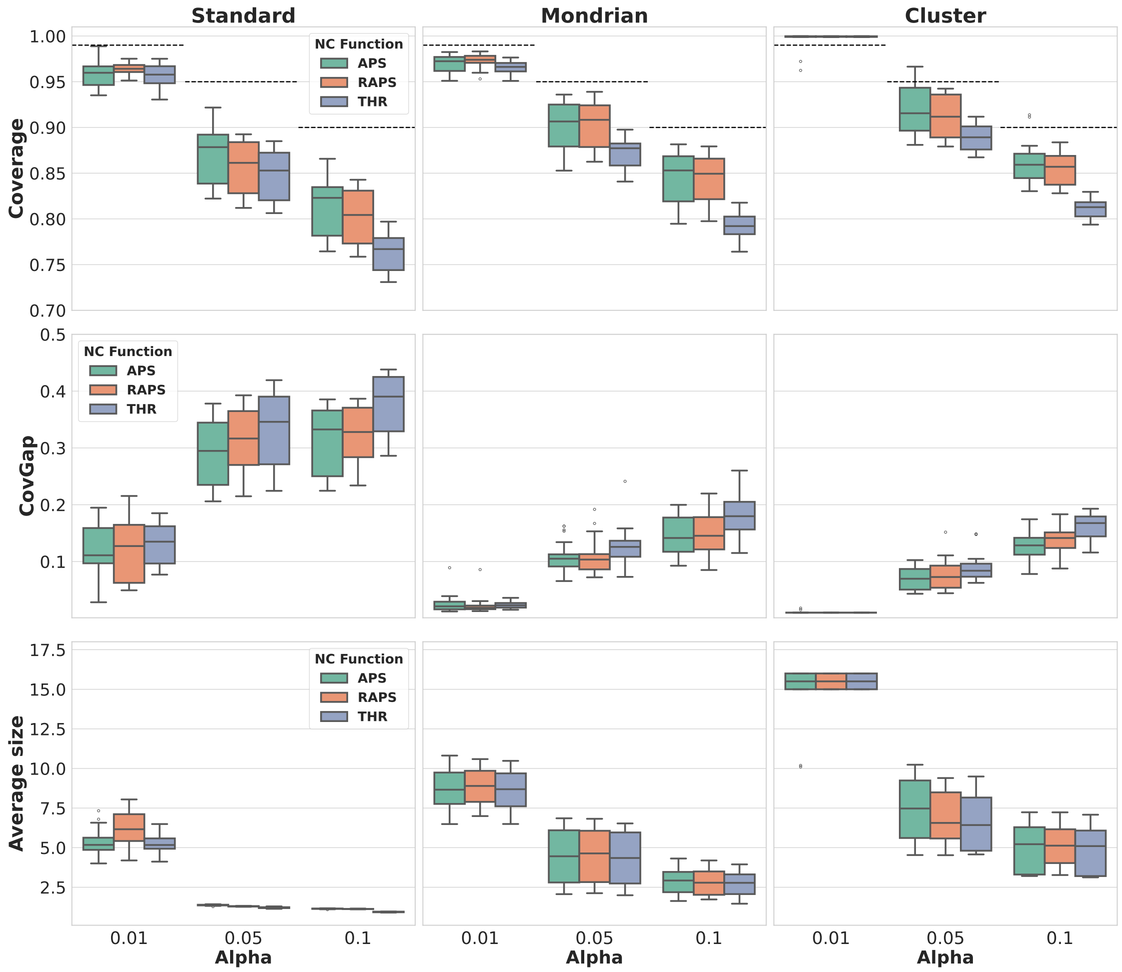


Figure S22: Averaged results for the **breast** tissue query data using **Celltypist** as underlying model. The columns represent the three taxonomies compared: standard, classwise and cluster. Each row displays the results for one of the three measured performance scores: coverage, covgap, and set size. Within each chart, every column corresponds to a nominal error level $\alpha = 0.01, 0.05, 0.10$, and the different non-conformity functions: $s_{\mathsf{APS}}$, $s_{\mathsf{RAPS}}$ and $s_{\mathsf{THR}}$, are indicated by distinct colors.

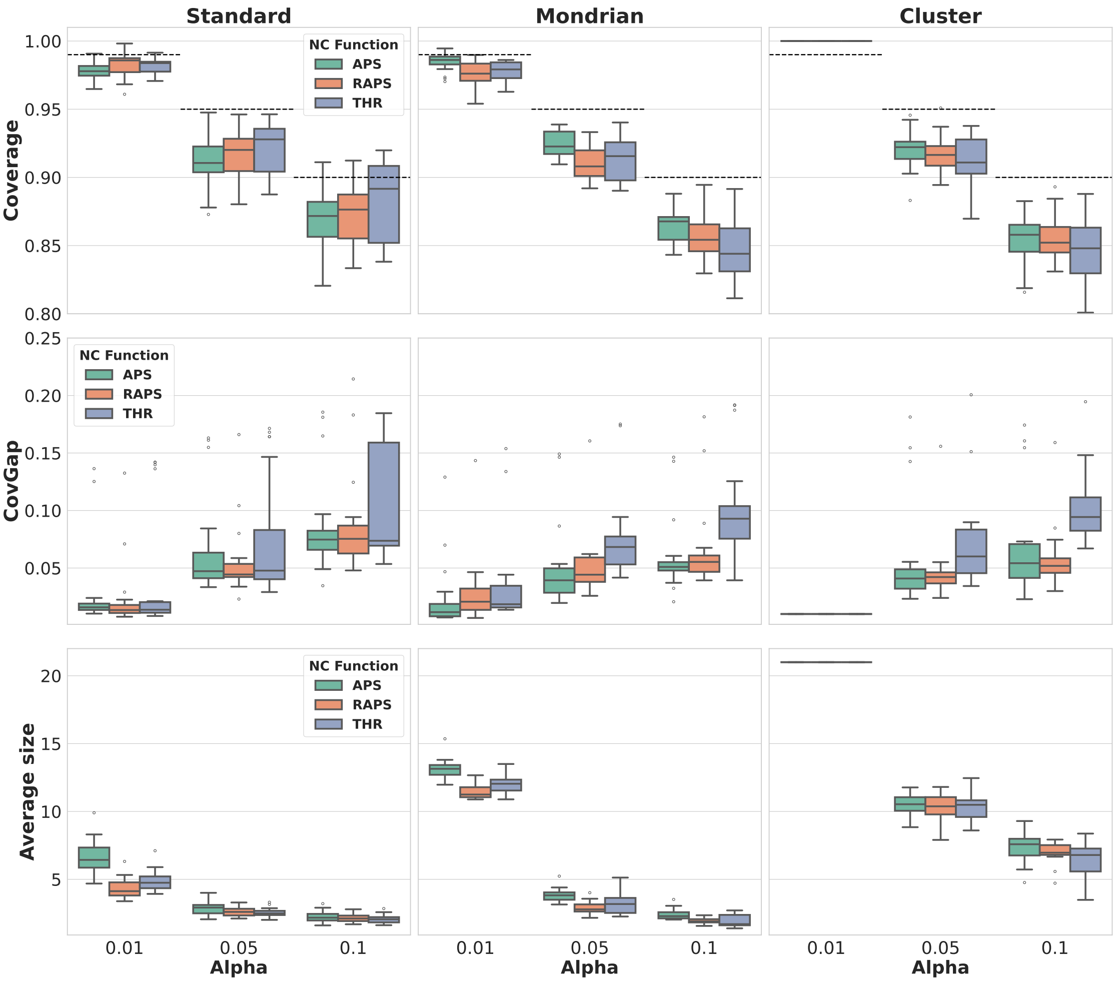


Figure S23: Averaged results for the **Lung healthy** query data using **TorchNet** as underlying model. The columns represent the three taxonomies compared: standard, classwise and cluster. Each row displays the results for one of the three measured performance scores: coverage, covgap, and set size. Within each chart, every column corresponds to a nominal error level $\alpha = 0.01, 0.05, 0.10$, and the different non-conformity functions: $s_{\mathsf{APS}}$, $s_{\mathsf{RAPS}}$ and $s_{\mathsf{THR}}$, are indicated by distinct colors.

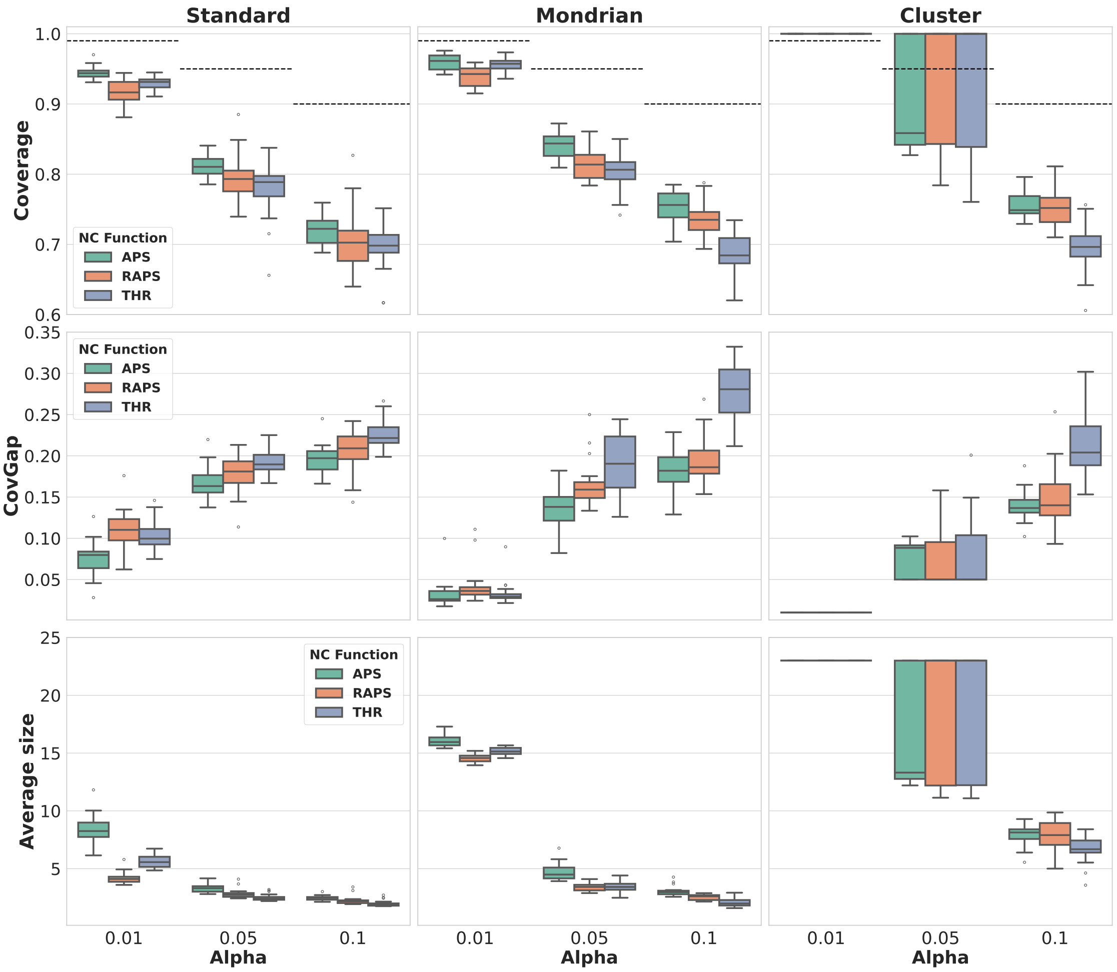


Figure S24: Averaged results for the **Lung tumoral** query data using **TorchNet** as underlying model. The columns represent the three taxonomies compared: standard, classwise and cluster. Each row displays the results for one of the three measured performance scores: coverage, covgap, and set size. Within each chart, every column corresponds to a nominal error level $\alpha = 0.01, 0.05, 0.10$, and the different non-conformity functions: $s_{\text{APS}}$, $s_{\text{RAPS}}$ and $s_{\text{THR}}$, are indicated by distinct colors.

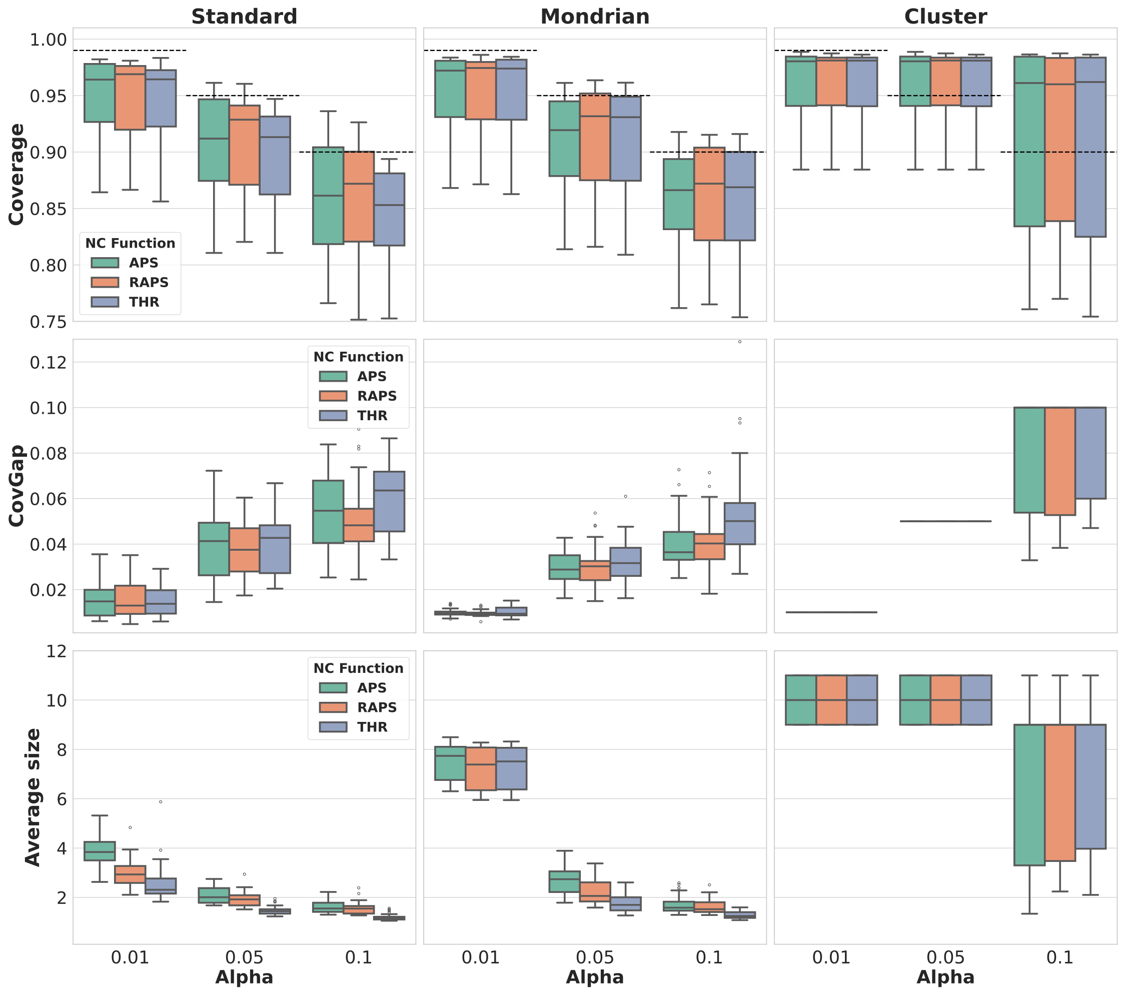


Figure S25: Averaged results for the **Gastrula** tissue query data using **TorchNet** as underlying model. The columns represent the three taxonomies compared: standard, classwise and cluster. Each row displays the results for one of the three measured performance scores: coverage, covgap, and set size. Within each chart, every column corresponds to a nominal error level $\alpha = 0.01, 0.05, 0.10$, and the different non-conformity functions: $s_{\mathsf{APS}}$, $s_{\mathsf{RAPS}}$ and $s_{\mathsf{THR}}$, are indicated by distinct colors.

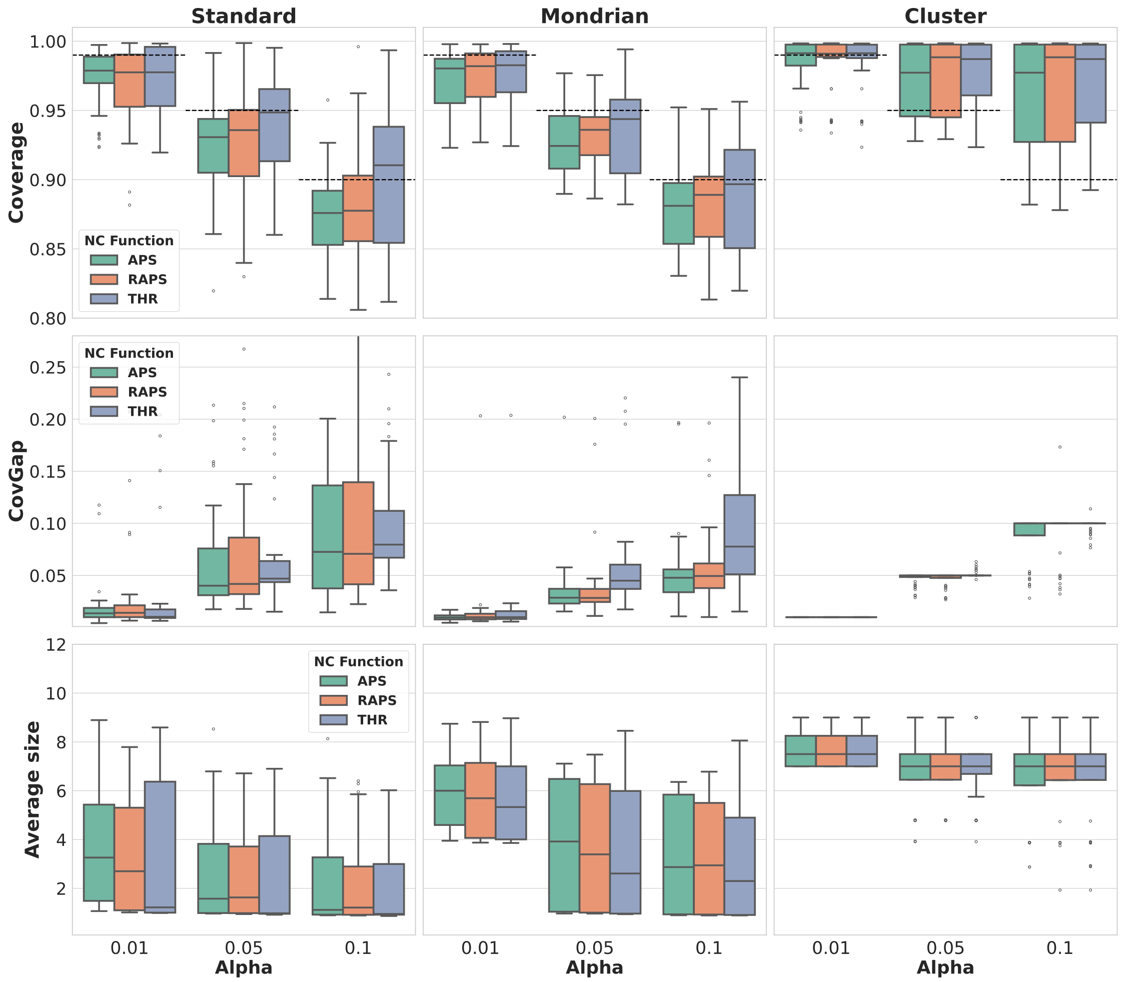


Figure S26: Averaged results for the **Pancreas** tissue query data using **TorchNet** as underlying model. The columns represent the three taxonomies compared: standard, classwise and cluster. Each row displays the results for one of the three measured performance scores: coverage, covgap, and set size. Within each chart, every column corresponds to a nominal error level $\alpha = 0.01, 0.05, 0.10$, and the different non-conformity functions: $s_{\text{APS}}$, $s_{\text{RAPS}}$ and $s_{\text{THR}}$, are indicated by distinct colors.

### S6.1 Tabular results

Finally, we provide the results of our experimental analyses summarized in tabular form. Tables S10, S11, and S12 detail the outcomes derived from experiments using the three distinct underlying models. In addition, Tables S13, S14, S15, and S16 present the results corresponding to experiments conducted with healthy lung immune tissues, lung immune tissue from patients with lung tumors, gastrula, and pancreatic tissues, respectively.

| Taxonomy | NC-funct | $\alpha$ | Test data | | | Query data | | |
|---|---|---|---|---|---|---|---|---|
| | | | Coverage (mean ± std) | CovGap (mean ± std) | Set size (mean ± std) | Coverage (mean ± std) | CovGap (mean ± std) | Set size (mean ± std) |
| standard | APS | .01 | $.99_{.00}$ | $.02_{.01}$ | $6.94_{1.07}$ | $.99_{.01}$ | $.05_{.05}$ | $7.11_{1.15}$ |
| | | .05 | $.95_{.00}$ | $.06_{.02}$ | $2.59_{.73}$ | $.95_{.01}$ | $.13_{.06}$ | $2.66_{.70}$ |
| | | .10 | $.90_{.01}$ | $.08_{.03}$ | $1.84_{.47}$ | $.89_{.01}$ | $.16_{.06}$ | $1.92_{.45}$ |
| | RAPS | .01 | $.99_{.00}$ | $.03_{.02}$ | $3.04_{.53}$ | $.98_{.00}$ | $.07_{.03}$ | $3.06_{.52}$ |
| | | .05 | $.95_{.00}$ | $.06_{.02}$ | $2.16_{.44}$ | $.95_{.01}$ | $.14_{.06}$ | $2.22_{.40}$ |
| | | .10 | $.90_{.01}$ | $.08_{.02}$ | $1.71_{.35}$ | $.89_{.01}$ | $.17_{.06}$ | $1.78_{.32}$ |
| | THR | .01 | $.99_{.00}$ | $.03_{.02}$ | $4.28_{.82}$ | $.99_{.01}$ | $.08_{.06}$ | $4.38_{.85}$ |
| | | .05 | $.95_{.01}$ | $.07_{.02}$ | $1.79_{.27}$ | $.94_{.01}$ | $.16_{.07}$ | $1.86_{.23}$ |
| | | .10 | $.90_{.01}$ | $.11_{.02}$ | $1.44_{.24}$ | $.87_{.02}$ | $.20_{.06}$ | $1.48_{.20}$ |
| classwise | APS | .01 | $.99_{.00}$ | $.01_{.00}$ | $8.90_{1.62}$ | $.99_{.00}$ | $.01_{.00}$ | $9.04_{1.49}$ |
| | | .05 | $.95_{.00}$ | $.03_{.01}$ | $4.65_{2.00}$ | $.94_{.01}$ | $.06_{.03}$ | $4.73_{1.94}$ |
| | | .10 | $.90_{.01}$ | $.05_{.02}$ | $2.97_{1.13}$ | $.88_{.01}$ | $.10_{.05}$ | $3.03_{1.07}$ |
| | RAPS | .01 | $.99_{.00}$ | $.01_{.00}$ | $7.28_{1.63}$ | $.99_{.00}$ | $.01_{.00}$ | $7.35_{1.58}$ |
| | | .05 | $.95_{.00}$ | $.03_{.01}$ | $4.38_{2.04}$ | $.94_{.01}$ | $.06_{.03}$ | $4.44_{1.98}$ |
| | | .10 | $.90_{.01}$ | $.04_{.01}$ | $2.79_{.91}$ | $.88_{.01}$ | $.10_{.04}$ | $2.83_{.84}$ |
| | THR | .01 | $.99_{.00}$ | $.01_{.00}$ | $7.92_{1.91}$ | $.99_{.00}$ | $.01_{.01}$ | $8.02_{1.79}$ |
| | | .05 | $.95_{.00}$ | $.03_{.01}$ | $4.06_{1.92}$ | $.93_{.02}$ | $.09_{.03}$ | $4.14_{1.84}$ |
| | | .10 | $.90_{.01}$ | $.05_{.01}$ | $2.62_{1.04}$ | $.86_{.02}$ | $.15_{.04}$ | $2.66_{.98}$ |
| cluster | APS | .01 | $1.00_{.00}$ | $.01_{.00}$ | $15.39_{.93}$ | $1.00_{.00}$ | $.01_{.00}$ | $15.39_{.92}$ |
| | | .05 | $1.00_{.00}$ | $.05_{.00}$ | $15.50_{.51}$ | $1.00_{.00}$ | $.05_{.00}$ | $15.50_{.51}$ |
| | | .10 | $1.00_{.00}$ | $.10_{.00}$ | $15.50_{.51}$ | $1.00_{.00}$ | $.10_{.00}$ | $15.50_{.51}$ |
| | RAPS | .01 | $1.00_{.00}$ | $.01_{.00}$ | $15.36_{1.07}$ | $1.00_{.00}$ | $.01_{.00}$ | $15.37_{1.05}$ |
| | | .05 | $1.00_{.00}$ | $.05_{.00}$ | $15.50_{.51}$ | $1.00_{.00}$ | $.05_{.00}$ | $15.50_{.51}$ |
| | | .10 | $1.00_{.00}$ | $.10_{.00}$ | $15.50_{.51}$ | $1.00_{.00}$ | $.10_{.00}$ | $15.50_{.51}$ |
| | THR | .01 | $1.00_{.00}$ | $.01_{.00}$ | $15.37_{1.04}$ | $1.00_{.00}$ | $.01_{.00}$ | $15.37_{1.01}$ |
| | | .05 | $1.00_{.00}$ | $.05_{.00}$ | $15.50_{.51}$ | $1.00_{.00}$ | $.05_{.00}$ | $15.50_{.51}$ |
| | | .10 | $1.00_{.00}$ | $.10_{.00}$ | $15.50_{.51}$ | $1.00_{.00}$ | $.10_{.00}$ | $15.50_{.51}$ |

Table S10: Averaged performance scores for **Breast** tissue using **TorchNet**. Test data represent a small, unseen subset of the reference data (10%) containing no OOD samples and free from batch effects (*i.e.*, similar to validation sets in traditional ML pipelines). This set serves as a baseline to compare the performance of the annotator in the query data, which includes both OOD samples and batch effects, against ideal conditions.

| Taxonomy | NC-funct | $\alpha$ | Test data | | | Query data | | |
|---|---|---|---|---|---|---|---|---|
| | | | Coverage (mean ± std) | CovGap (mean ± std) | Set size (mean ± std) | Coverage (mean ± std) | CovGap (mean ± std) | Set size (mean ± std) |
| standard | APS | .01 | $1.00_{.00}$ | $.01_{.00}$ | $15.50_{.51}$ | $1.00_{.00}$ | $.01_{.00}$ | $15.50_{.51}$ |
| | | .05 | $.95_{.00}$ | $.17_{.04}$ | $1.15_{.01}$ | $.88_{.03}$ | $.28_{.05}$ | $1.22_{.03}$ |
| | | .10 | $.90_{.01}$ | $.17_{.04}$ | $1.07_{.01}$ | $.83_{.03}$ | $.29_{.04}$ | $1.13_{.03}$ |
| | RAPS | .01 | $.99_{.00}$ | $.02_{.01}$ | $10.38_{.70}$ | $.97_{.03}$ | $.08_{.05}$ | $10.38_{.70}$ |
| | | .05 | $.95_{.01}$ | $.17_{.04}$ | $1.14_{.01}$ | $.88_{.03}$ | $.30_{.04}$ | $1.21_{.03}$ |
| | | .10 | $.90_{.01}$ | $.17_{.04}$ | $1.06_{.01}$ | $.83_{.02}$ | $.31_{.04}$ | $1.12_{.03}$ |
| | THR | .01 | $1.00_{.00}$ | $.01_{.00}$ | $15.50_{.51}$ | $1.00_{.00}$ | $.01_{.00}$ | $15.50_{.51}$ |
| | | .05 | $.96_{.00}$ | $.16_{.04}$ | $1.16_{.01}$ | $.89_{.03}$ | $.29_{.04}$ | $1.24_{.03}$ |
| | | .10 | $.91_{.01}$ | $.22_{.05}$ | $.99_{.00}$ | $.81_{.03}$ | $.41_{.04}$ | $.99_{.01}$ |
| classwise | APS | .01 | $1.00_{.00}$ | $.01_{.00}$ | $13.72_{.58}$ | $1.00_{.00}$ | $.01_{.00}$ | $13.72_{.57}$ |
| | | .05 | $.96_{.01}$ | $.03_{.00}$ | $8.76_{1.64}$ | $.96_{.01}$ | $.07_{.02}$ | $8.80_{1.62}$ |
| | | .10 | $.91_{.01}$ | $.05_{.01}$ | $6.20_{1.47}$ | $.91_{.02}$ | $.12_{.03}$ | $6.24_{1.47}$ |
| | RAPS | .01 | $1.00_{.00}$ | $.01_{.00}$ | $12.80_{.64}$ | $1.00_{.00}$ | $.01_{.00}$ | $12.86_{.63}$ |
| | | .05 | $.96_{.01}$ | $.03_{.01}$ | $7.75_{1.38}$ | $.96_{.01}$ | $.08_{.03}$ | $7.87_{1.37}$ |
| | | .10 | $.90_{.01}$ | $.05_{.01}$ | $5.69_{1.19}$ | $.90_{.02}$ | $.12_{.03}$ | $5.81_{1.21}$ |
| | THR | .01 | $1.00_{.00}$ | $.01_{.00}$ | $13.72_{.58}$ | $1.00_{.00}$ | $.01_{.00}$ | $13.72_{.57}$ |
| | | .05 | $.98_{.01}$ | $.04_{.01}$ | $8.76_{1.65}$ | $.97_{.01}$ | $.09_{.03}$ | $8.80_{1.63}$ |
| | | .10 | $.93_{.01}$ | $.06_{.01}$ | $6.19_{1.46}$ | $.93_{.02}$ | $.16_{.03}$ | $6.19_{1.47}$ |
| cluster | APS | .01 | $1.00_{.00}$ | $.01_{.00}$ | $15.50_{.50}$ | $1.00_{.00}$ | $.01_{.00}$ | $15.50_{.50}$ |
| | | .05 | $.99_{.02}$ | $.04_{.01}$ | $13.22_{3.50}$ | $.99_{.02}$ | $.06_{.02}$ | $13.24_{3.48}$ |
| | | .10 | $.97_{.05}$ | $.08_{.02}$ | $12.49_{4.41}$ | $.96_{.05}$ | $.11_{.02}$ | $12.50_{4.39}$ |
| | RAPS | .01 | $1.00_{.00}$ | $.01_{.00}$ | $15.50_{.50}$ | $1.00_{.00}$ | $.01_{.00}$ | $15.50_{.50}$ |
| | | .05 | $.99_{.02}$ | $.04_{.01}$ | $12.99_{3.72}$ | $.98_{.03}$ | $.06_{.02}$ | $13.02_{3.68}$ |
| | | .10 | $.97_{.05}$ | $.08_{.02}$ | $12.27_{4.70}$ | $.96_{.05}$ | $.11_{.02}$ | $12.29_{4.67}$ |
| | THR | .01 | $1.00_{.00}$ | $.01_{.00}$ | $15.46_{.60}$ | $1.00_{.00}$ | $.01_{.00}$ | $15.46_{.60}$ |
| | | .05 | $.99_{.01}$ | $.05_{.01}$ | $13.40_{3.23}$ | $.99_{.01}$ | $.06_{.02}$ | $13.42_{3.20}$ |
| | | .10 | $.98_{.03}$ | $.09_{.02}$ | $12.54_{4.37}$ | $.97_{.04}$ | $.12_{.04}$ | $12.54_{4.37}$ |

Table S11: Averaged performance scores for **Breast** tissue using **Scmap**. Test data represent a small, unseen subset of the reference data (10%) containing no OOD samples and free from batch effects (*i.e.*, similar to validation sets in traditional ML pipelines). This set serves as a baseline to compare the performance of the annotator in the query data, which includes both OOD samples and batch effects, against ideal conditions.

| Taxonomy | NC-funct | $\alpha$ | Test data | | | Query data | | |
|---|---|---|---|---|---|---|---|---|
| | | | Coverage (mean ± std) | CovGap (mean ± std) | Set size (mean ± std) | Coverage (mean ± std) | CovGap (mean ± std) | Set size (mean ± std) |
| standard | APS | .01 | $.99_{.00}$ | $.05_{.03}$ | $6.06_{.95}$ | $.96_{.01}$ | $.13_{.04}$ | $5.27_{.71}$ |
| | | .05 | $.95_{.00}$ | $.17_{.05}$ | $1.21_{.04}$ | $.87_{.03}$ | $.29_{.06}$ | $1.37_{.03}$ |
| | | .10 | $.90_{.01}$ | $.18_{.05}$ | $1.06_{.02}$ | $.81_{.03}$ | $.31_{.06}$ | $1.14_{.02}$ |
| | RAPS | .01 | $.99_{.00}$ | $.04_{.03}$ | $6.24_{1.15}$ | $.96_{.01}$ | $.12_{.05}$ | $6.13_{1.10}$ |
| | | .05 | $.95_{.01}$ | $.17_{.05}$ | $1.17_{.03}$ | $.86_{.03}$ | $.31_{.06}$ | $1.30_{.02}$ |
| | | .10 | $.90_{.01}$ | $.19_{.05}$ | $1.05_{.01}$ | $.80_{.03}$ | $.32_{.05}$ | $1.13_{.01}$ |
| | THR | .01 | $.99_{.00}$ | $.04_{.03}$ | $5.74_{1.11}$ | $.96_{.01}$ | $.13_{.03}$ | $5.19_{.55}$ |
| | | .05 | $.95_{.00}$ | $.19_{.06}$ | $1.13_{.03}$ | $.85_{.03}$ | $.33_{.06}$ | $1.21_{.04}$ |
| | | .10 | $.90_{.01}$ | $.24_{.05}$ | $.97_{.01}$ | $.76_{.02}$ | $.38_{.05}$ | $.94_{.02}$ |
| classwise | APS | .01 | $.99_{.00}$ | $.01_{.00}$ | $8.78_{1.10}$ | $.97_{.01}$ | $.02_{.01}$ | $8.75_{1.17}$ |
| | | .05 | $.95_{.00}$ | $.03_{.01}$ | $4.53_{1.65}$ | $.90_{.03}$ | $.11_{.02}$ | $4.45_{1.72}$ |
| | | .10 | $.90_{.01}$ | $.05_{.02}$ | $2.99_{.77}$ | $.84_{.03}$ | $.15_{.03}$ | $2.87_{.78}$ |
| | RAPS | .01 | $.99_{.00}$ | $.01_{.00}$ | $8.65_{1.12}$ | $.97_{.01}$ | $.02_{.01}$ | $8.87_{1.08}$ |
| | | .05 | $.95_{.01}$ | $.03_{.01}$ | $4.43_{1.71}$ | $.90_{.02}$ | $.11_{.03}$ | $4.48_{1.71}$ |
| | | .10 | $.90_{.01}$ | $.05_{.01}$ | $2.79_{.81}$ | $.84_{.03}$ | $.15_{.03}$ | $2.81_{.78}$ |
| | THR | .01 | $.99_{.00}$ | $.01_{.00}$ | $8.71_{1.10}$ | $.97_{.01}$ | $.02_{.01}$ | $8.68_{1.16}$ |
| | | .05 | $.95_{.00}$ | $.03_{.01}$ | $4.44_{1.67}$ | $.87_{.01}$ | $.13_{.03}$ | $4.33_{1.69}$ |
| | | .10 | $.90_{.01}$ | $.05_{.01}$ | $2.87_{.80}$ | $.79_{.01}$ | $.18_{.03}$ | $2.70_{.75}$ |
| cluster | APS | .01 | $1.00_{.00}$ | $.01_{.00}$ | $15.26_{1.29}$ | $1.00_{.01}$ | $.01_{.00}$ | $15.26_{1.26}$ |
| | | .05 | $.98_{.02}$ | $.04_{.01}$ | $12.73_{4.15}$ | $.97_{.04}$ | $.06_{.01}$ | $12.76_{4.10}$ |
| | | .10 | $.97_{.05}$ | $.08_{.03}$ | $12.01_{5.07}$ | $.95_{.07}$ | $.11_{.02}$ | $12.05_{5.02}$ |
| | RAPS | .01 | $1.00_{.00}$ | $.01_{.00}$ | $15.50_{.50}$ | $1.00_{.00}$ | $.01_{.00}$ | $15.50_{.50}$ |
| | | .05 | $.98_{.02}$ | $.04_{.01}$ | $12.62_{4.25}$ | $.97_{.04}$ | $.06_{.02}$ | $12.67_{4.19}$ |
| | | .10 | $.97_{.05}$ | $.08_{.02}$ | $11.97_{5.10}$ | $.95_{.07}$ | $.11_{.02}$ | $12.01_{5.05}$ |
| | THR | .01 | $1.00_{.00}$ | $.01_{.00}$ | $15.50_{.50}$ | $1.00_{.00}$ | $.01_{.00}$ | $15.50_{.50}$ |
| | | .05 | $.98_{.02}$ | $.04_{.01}$ | $12.52_{4.38}$ | $.96_{.05}$ | $.06_{.03}$ | $12.54_{4.35}$ |
| | | .10 | $.97_{.05}$ | $.08_{.02}$ | $11.93_{5.18}$ | $.94_{.09}$ | $.12_{.03}$ | $11.94_{5.16}$ |

Table S12: Averaged performance scores for **Breast** tissue using **CellTypist**. Test data represent a small, unseen subset of the reference data (10%) containing no OOD samples and free from batch effects (*i.e.*, similar to validation sets in traditional ML pipelines). This set serves as a baseline to compare the performance of the annotator in the query data, which includes both OOD samples and batch effects, against ideal conditions.

| Taxonomy | NC-funct | $\alpha$ | Test data | | | Query data | | |
|---|---|---|---|---|---|---|---|---|
| | | | Coverage (mean $\pm$ std) | CovGap (mean $\pm$ std) | Set size (mean $\pm$ std) | Coverage (mean $\pm$ std) | CovGap (mean $\pm$ std) | Set size (mean $\pm$ std) |
| standard | APS | .01 | $.99_{.00}$ | $.02_{.01}$ | $8.35_{1.27}$ | $.98_{.01}$ | $.03_{.04}$ | $6.61_{1.21}$ |
| | | .05 | $.95_{.01}$ | $.05_{.01}$ | $3.99_{.65}$ | $.91_{.02}$ | $.07_{.04}$ | $2.93_{.52}$ |
| | | .10 | $.90_{.01}$ | $.07_{.01}$ | $3.03_{.53}$ | $.87_{.03}$ | $.09_{.04}$ | $2.25_{.42}$ |
| | RAPS | .01 | $.99_{.00}$ | $.02_{.01}$ | $4.86_{.81}$ | $.98_{.01}$ | $.02_{.03}$ | $4.30_{.76}$ |
| | | .05 | $.95_{.01}$ | $.05_{.01}$ | $3.21_{.43}$ | $.92_{.02}$ | $.06_{.03}$ | $2.62_{.36}$ |
| | | .10 | $.90_{.01}$ | $.07_{.01}$ | $2.69_{.40}$ | $.87_{.02}$ | $.09_{.04}$ | $2.15_{.32}$ |
| | THR | .01 | $.99_{.00}$ | $.02_{.01}$ | $6.48_{1.25}$ | $.98_{.01}$ | $.04_{.05}$ | $4.89_{.74}$ |
| | | .05 | $.95_{.00}$ | $.05_{.01}$ | $3.06_{.51}$ | $.92_{.02}$ | $.07_{.05}$ | $2.54_{.33}$ |
| | | .10 | $.90_{.01}$ | $.08_{.01}$ | $2.40_{.46}$ | $.88_{.03}$ | $.10_{.05}$ | $2.06_{.32}$ |
| classwise | APS | .01 | $.99_{.00}$ | $.01_{.00}$ | $14.57_{.85}$ | $.98_{.01}$ | $.02_{.03}$ | $13.15_{.71}$ |
| | | .05 | $.95_{.00}$ | $.04_{.01}$ | $4.87_{.64}$ | $.92_{.01}$ | $.05_{.04}$ | $3.84_{.48}$ |
| | | .10 | $.90_{.01}$ | $.05_{.01}$ | $3.12_{.53}$ | $.87_{.01}$ | $.06_{.03}$ | $2.41_{.39}$ |
| | RAPS | .01 | $.99_{.00}$ | $.01_{.00}$ | $11.89_{.58}$ | $.97_{.01}$ | $.03_{.03}$ | $11.50_{.59}$ |
| | | .05 | $.95_{.01}$ | $.04_{.01}$ | $3.20_{.46}$ | $.91_{.01}$ | $.05_{.03}$ | $2.88_{.47}$ |
| | | .10 | $.90_{.01}$ | $.05_{.01}$ | $2.30_{.19}$ | $.86_{.02}$ | $.07_{.04}$ | $1.93_{.18}$ |
| | THR | .01 | $.99_{.00}$ | $.01_{.00}$ | $12.97_{.85}$ | $.98_{.01}$ | $.03_{.04}$ | $12.06_{.67}$ |
| | | .05 | $.95_{.01}$ | $.04_{.01}$ | $3.65_{.82}$ | $.91_{.01}$ | $.07_{.04}$ | $3.28_{.83}$ |
| | | .10 | $.90_{.01}$ | $.05_{.01}$ | $2.07_{.52}$ | $.85_{.02}$ | $.10_{.04}$ | $1.91_{.45}$ |
| cluster | APS | .01 | $1.00_{.00}$ | $.01_{.00}$ | $21.00_{.00}$ | $1.00_{.00}$ | $.01_{.00}$ | $21.00_{.00}$ |
| | | .05 | $.95_{.01}$ | $.03_{.00}$ | $11.27_{.74}$ | $.92_{.02}$ | $.06_{.05}$ | $10.51_{.70}$ |
| | | .10 | $.90_{.01}$ | $.06_{.01}$ | $7.91_{1.13}$ | $.85_{.02}$ | $.07_{.04}$ | $7.39_{1.12}$ |
| | RAPS | .01 | $1.00_{.00}$ | $.01_{.00}$ | $21.00_{.00}$ | $1.00_{.00}$ | $.01_{.00}$ | $21.00_{.00}$ |
| | | .05 | $.95_{.01}$ | $.03_{.00}$ | $10.79_{.99}$ | $.92_{.01}$ | $.05_{.03}$ | $10.35_{1.06}$ |
| | | .10 | $.90_{.01}$ | $.06_{.00}$ | $7.33_{.75}$ | $.86_{.02}$ | $.06_{.03}$ | $7.01_{.76}$ |
| | THR | .01 | $1.00_{.00}$ | $.01_{.00}$ | $21.00_{.00}$ | $1.00_{.00}$ | $.01_{.00}$ | $21.00_{.00}$ |
| | | .05 | $.95_{.01}$ | $.04_{.00}$ | $10.62_{1.10}$ | $.91_{.02}$ | $.07_{.04}$ | $10.28_{1.07}$ |
| | | .10 | $.90_{.01}$ | $.06_{.01}$ | $6.64_{1.13}$ | $.85_{.02}$ | $.10_{.03}$ | $6.48_{1.15}$ |

Table S13: Averaged performance scores for the **Lung** healthy query data using **TorchNet**. Test data represent a small, unseen subset of the reference data (10%) containing no OOD samples and free from batch effects (*i.e.*, similar to validation sets in traditional ML pipelines). This set serves as a baseline to compare the performance of the annotator in the query data, which includes both OOD samples and batch effects, against ideal conditions.

| Taxonomy | NC-funct | $\alpha$ | Test data | | | Query data | | |
|---|---|---|---|---|---|---|---|---|
| | | | Coverage (mean ± std) | CovGap (mean ± std) | Set size (mean ± std) | Coverage (mean ± std) | CovGap (mean ± std) | Set size (mean ± std) |
| standard | APS | .01 | $.99_{.00}$ | $.02_{.01}$ | $9.41_{1.50}$ | $.94_{.01}$ | $.07_{.02}$ | $8.33_{1.27}$ |
| | | .05 | $.95_{.00}$ | $.05_{.01}$ | $3.83_{.64}$ | $.81_{.02}$ | $.17_{.02}$ | $3.30_{.34}$ |
| | | .10 | $.90_{.01}$ | $.07_{.01}$ | $2.77_{.42}$ | $.72_{.02}$ | $.20_{.02}$ | $2.46_{.21}$ |
| | RAPS | .01 | $.99_{.00}$ | $.02_{.01}$ | $4.47_{.61}$ | $.92_{.02}$ | $.11_{.02}$ | $4.15_{.51}$ |
| | | .05 | $.95_{.01}$ | $.05_{.01}$ | $3.04_{.53}$ | $.80_{.03}$ | $.18_{.02}$ | $2.83_{.41}$ |
| | | .10 | $.90_{.01}$ | $.07_{.01}$ | $2.40_{.48}$ | $.70_{.04}$ | $.21_{.02}$ | $2.26_{.37}$ |
| | THR | .01 | $.99_{.00}$ | $.02_{.01}$ | $6.38_{.85}$ | $.93_{.01}$ | $.10_{.02}$ | $5.63_{.56}$ |
| | | .05 | $.95_{.01}$ | $.05_{.01}$ | $2.51_{.44}$ | $.78_{.04}$ | $.19_{.02}$ | $2.51_{.29}$ |
| | | .10 | $.90_{.01}$ | $.08_{.01}$ | $1.98_{.37}$ | $.69_{.05}$ | $.23_{.02}$ | $1.98_{.27}$ |
| classwise | APS | .01 | $.99_{.00}$ | $.01_{.00}$ | $17.08_{.77}$ | $.96_{.01}$ | $.03_{.02}$ | $16.03_{.49}$ |
| | | .05 | $.95_{.01}$ | $.04_{.01}$ | $5.06_{1.13}$ | $.84_{.02}$ | $.14_{.03}$ | $4.68_{.73}$ |
| | | .10 | $.90_{.01}$ | $.05_{.01}$ | $3.20_{.71}$ | $.75_{.02}$ | $.18_{.03}$ | $3.06_{.42}$ |
| | RAPS | .01 | $.99_{.00}$ | $.01_{.00}$ | $14.89_{.44}$ | $.94_{.02}$ | $.04_{.02}$ | $14.53_{.37}$ |
| | | .05 | $.95_{.00}$ | $.04_{.01}$ | $3.36_{.51}$ | $.81_{.02}$ | $.17_{.03}$ | $3.42_{.34}$ |
| | | .10 | $.90_{.01}$ | $.06_{.01}$ | $2.43_{.29}$ | $.74_{.03}$ | $.19_{.03}$ | $2.54_{.24}$ |
| | THR | .01 | $.99_{.00}$ | $.01_{.00}$ | $15.67_{.54}$ | $.96_{.01}$ | $.03_{.01}$ | $15.16_{.34}$ |
| | | .05 | $.95_{.01}$ | $.04_{.01}$ | $3.00_{.55}$ | $.80_{.03}$ | $.19_{.04}$ | $3.42_{.44}$ |
| | | .10 | $.90_{.01}$ | $.06_{.01}$ | $1.63_{.37}$ | $.69_{.03}$ | $.28_{.03}$ | $2.04_{.33}$ |
| cluster | APS | .01 | $1.00_{.00}$ | $.01_{.00}$ | $23.00_{.00}$ | $1.00_{.00}$ | $.01_{.00}$ | $23.00_{.00}$ |
| | | .05 | $.97_{.02}$ | $.04_{.01}$ | $17.13_{4.94}$ | $.91_{.08}$ | $.08_{.02}$ | $16.91_{5.11}$ |
| | | .10 | $.90_{.01}$ | $.05_{.01}$ | $8.09_{.91}$ | $.76_{.02}$ | $.14_{.02}$ | $7.86_{.87}$ |
| | RAPS | .01 | $1.00_{.00}$ | $.01_{.00}$ | $23.00_{.00}$ | $1.00_{.00}$ | $.01_{.00}$ | $23.00_{.00}$ |
| | | .05 | $.98_{.03}$ | $.04_{.01}$ | $18.01_{5.67}$ | $.92_{.09}$ | $.08_{.04}$ | $18.01_{5.67}$ |
| | | .10 | $.90_{.01}$ | $.06_{.01}$ | $7.82_{1.17}$ | $.75_{.03}$ | $.15_{.04}$ | $7.85_{1.17}$ |
| | THR | .01 | $1.00_{.00}$ | $.01_{.00}$ | $23.00_{.00}$ | $1.00_{.00}$ | $.01_{.00}$ | $23.00_{.00}$ |
| | | .05 | $.98_{.02}$ | $.04_{.01}$ | $17.98_{5.70}$ | $.92_{.09}$ | $.08_{.04}$ | $18.06_{5.61}$ |
| | | .10 | $.90_{.01}$ | $.06_{.01}$ | $6.37_{1.18}$ | $.70_{.04}$ | $.21_{.04}$ | $6.61_{1.14}$ |

Table S14: Averaged performance scores for the **Lung** tumoral query data using **TorchNet**. Test data represent a small, unseen subset of the reference data (10%) containing no OOD samples and free from batch effects (*i.e.*, similar to validation sets in traditional ML pipelines). This set serves as a baseline to compare the performance of the annotator in the query data, which includes both OOD samples and batch effects, against ideal conditions.

| Taxonomy | NC-funct | $\alpha$ | Test data | | | Query data | | |
|---|---|---|---|---|---|---|---|---|
| | | | Coverage (mean ± std) | CovGap (mean ± std) | Set size (mean ± std) | Coverage (mean ± std) | CovGap (mean ± std) | Set size (mean ± std) |
| standard | APS | .01 | $.99_{.01}$ | $.02_{.01}$ | $3.66_{.50}$ | $.95_{.04}$ | $.02_{.01}$ | $3.92_{.60}$ |
| | | .05 | $.95_{.02}$ | $.05_{.02}$ | $1.97_{.23}$ | $.91_{.04}$ | $.04_{.01}$ | $2.14_{.31}$ |
| | | .10 | $.90_{.02}$ | $.07_{.02}$ | $1.53_{.18}$ | $.86_{.05}$ | $.06_{.02}$ | $1.64_{.22}$ |
| | RAPS | .01 | $.99_{.01}$ | $.02_{.01}$ | $2.81_{.43}$ | $.94_{.04}$ | $.02_{.01}$ | $3.01_{.54}$ |
| | | .05 | $.95_{.02}$ | $.05_{.02}$ | $1.82_{.22}$ | $.90_{.04}$ | $.04_{.01}$ | $1.95_{.29}$ |
| | | .10 | $.90_{.02}$ | $.07_{.02}$ | $1.46_{.17}$ | $.86_{.05}$ | $.05_{.01}$ | $1.57_{.23}$ |
| | THR | .01 | $.99_{.01}$ | $.02_{.01}$ | $2.42_{.61}$ | $.94_{.04}$ | $.01_{.01}$ | $2.63_{.75}$ |
| | | .05 | $.95_{.01}$ | $.04_{.01}$ | $1.41_{.14}$ | $.90_{.04}$ | $.04_{.01}$ | $1.48_{.18}$ |
| | | .10 | $.90_{.02}$ | $.07_{.02}$ | $1.17_{.11}$ | $.84_{.04}$ | $.06_{.02}$ | $1.20_{.13}$ |
| classwise | APS | .01 | $.99_{.01}$ | $.01_{.00}$ | $7.33_{.64}$ | $.95_{.04}$ | $.01_{.00}$ | $7.45_{.74}$ |
| | | .05 | $.95_{.01}$ | $.04_{.01}$ | $2.51_{.45}$ | $.91_{.04}$ | $.03_{.01}$ | $2.71_{.52}$ |
| | | .10 | $.90_{.02}$ | $.06_{.02}$ | $1.61_{.27}$ | $.86_{.04}$ | $.04_{.01}$ | $1.74_{.33}$ |
| | RAPS | .01 | $1.00_{.01}$ | $.01_{.00}$ | $7.08_{.82}$ | $.95_{.03}$ | $.01_{.00}$ | $7.21_{.92}$ |
| | | .05 | $.96_{.02}$ | $.04_{.01}$ | $2.14_{.48}$ | $.91_{.05}$ | $.03_{.01}$ | $2.31_{.54}$ |
| | | .10 | $.91_{.02}$ | $.05_{.01}$ | $1.53_{.21}$ | $.86_{.05}$ | $.04_{.01}$ | $1.64_{.26}$ |
| | THR | .01 | $1.00_{.00}$ | $.01_{.00}$ | $7.09_{.81}$ | $.95_{.04}$ | $.01_{.00}$ | $7.22_{.91}$ |
| | | .05 | $.96_{.02}$ | $.04_{.01}$ | $1.68_{.31}$ | $.91_{.04}$ | $.03_{.01}$ | $1.78_{.37}$ |
| | | .10 | $.91_{.02}$ | $.05_{.01}$ | $1.23_{.12}$ | $.86_{.05}$ | $.05_{.02}$ | $1.28_{.15}$ |
| cluster | APS | .01 | $1.00_{.00}$ | $.01_{.00}$ | $10.00_{1.01}$ | $.96_{.03}$ | $.01_{.00}$ | $10.00_{1.01}$ |
| | | .05 | $1.00_{.00}$ | $.05_{.00}$ | $10.00_{1.01}$ | $.96_{.03}$ | $.05_{.00}$ | $10.00_{1.01}$ |
| | | .10 | $.96_{.05}$ | $.08_{.02}$ | $6.21_{3.14}$ | $.91_{.08}$ | $.08_{.02}$ | $6.23_{3.12}$ |
| | RAPS | .01 | $1.00_{.00}$ | $.01_{.00}$ | $10.00_{1.01}$ | $.96_{.02}$ | $.01_{.00}$ | $10.00_{1.01}$ |
| | | .05 | $1.00_{.00}$ | $.05_{.00}$ | $10.00_{1.01}$ | $.96_{.02}$ | $.05_{.00}$ | $10.00_{1.01}$ |
| | | .10 | $.96_{.04}$ | $.08_{.02}$ | $6.41_{2.92}$ | $.91_{.07}$ | $.08_{.03}$ | $6.44_{2.89}$ |
| | THR | .01 | $1.00_{.00}$ | $.01_{.00}$ | $10.00_{1.01}$ | $.96_{.04}$ | $.01_{.00}$ | $10.00_{1.01}$ |
| | | .05 | $1.00_{.00}$ | $.05_{.00}$ | $10.00_{1.01}$ | $.96_{.04}$ | $.05_{.00}$ | $10.00_{1.01}$ |
| | | .10 | $.96_{.05}$ | $.08_{.02}$ | $6.79_{2.99}$ | $.91_{.08}$ | $.08_{.02}$ | $6.78_{2.99}$ |

Table S15: Averaged performance scores for **Gastrula** tissue using TorchNet. Test data represent a small, unseen subset of the reference data (10%) containing no OOD samples and free from batch effects (*i.e.*, similar to validation sets in traditional ML pipelines). This set serves as a baseline to compare the performance of the annotator in the query data, which includes both OOD samples and batch effects, against ideal conditions.

| Taxonomy | NC-funct | $\alpha$ | Test data Coverage (mean ± std) | Test data CovGap (mean ± std) | Test data Set size (mean ± std) | Query data Coverage (mean ± std) | Query data CovGap (mean ± std) | Query data Set size (mean ± std) |
|---|---|---|---|---|---|---|---|---|
| | | .01 | $.99_{.01}$ | $.03_{.03}$ | $3.69_{2.28}$ | $.97_{.02}$ | $.02_{.04}$ | $3.66_{2.29}$ |
| | APS | .05 | $.95_{.01}$ | $.06_{.04}$ | $2.74_{2.14}$ | $.92_{.03}$ | $.06_{.05}$ | $2.72_{2.13}$ |
| | | .10 | $.90_{.02}$ | $.08_{.05}$ | $2.40_{2.00}$ | $.87_{.04}$ | $.09_{.06}$ | $2.38_{2.01}$ |
| standard | | .01 | $.99_{.01}$ | $.02_{.02}$ | $3.39_{2.32}$ | $.97_{.03}$ | $.03_{.05}$ | $3.42_{2.33}$ |
| | RAPS | .05 | $.95_{.02}$ | $.06_{.03}$ | $2.60_{1.95}$ | $.93_{.04}$ | $.07_{.06}$ | $2.60_{1.93}$ |
| | | .10 | $.91_{.02}$ | $.09_{.06}$ | $2.16_{1.66}$ | $.88_{.05}$ | $.10_{.07}$ | $2.15_{1.66}$ |
| | | .01 | $.99_{.01}$ | $.01_{.00}$ | $3.47_{2.94}$ | $.97_{.03}$ | $.03_{.05}$ | $3.40_{2.89}$ |
| | THR | .05 | $.95_{.01}$ | $.04_{.01}$ | $2.58_{2.12}$ | $.94_{.05}$ | $.07_{.05}$ | $2.53_{2.11}$ |
| | | .10 | $.90_{.02}$ | $.08_{.02}$ | $2.15_{1.70}$ | $.90_{.06}$ | $.10_{.05}$ | $2.12_{1.71}$ |
| | | .01 | $.99_{.01}$ | $.01_{.00}$ | $5.98_{1.54}$ | $.97_{.02}$ | $.01_{.00}$ | $5.99_{1.54}$ |
| | APS | .05 | $.95_{.01}$ | $.04_{.01}$ | $3.85_{2.59}$ | $.93_{.02}$ | $.03_{.03}$ | $3.90_{2.59}$ |
| | | .10 | $.91_{.02}$ | $.06_{.02}$ | $3.35_{2.45}$ | $.88_{.03}$ | $.05_{.04}$ | $3.35_{2.41}$ |
| classwise | | .01 | $.99_{.01}$ | $.01_{.00}$ | $5.83_{1.77}$ | $.97_{.02}$ | $.02_{.03}$ | $5.83_{1.78}$ |
| | RAPS | .05 | $.96_{.02}$ | $.04_{.01}$ | $3.68_{2.62}$ | $.93_{.03}$ | $.04_{.04}$ | $3.70_{2.62}$ |
| | | .10 | $.91_{.02}$ | $.05_{.02}$ | $3.28_{2.40}$ | $.88_{.03}$ | $.06_{.04}$ | $3.31_{2.41}$ |
| | | .01 | $.99_{.00}$ | $.01_{.00}$ | $5.72_{1.76}$ | $.97_{.02}$ | $.02_{.03}$ | $5.75_{1.81}$ |
| | THR | .05 | $.96_{.02}$ | $.04_{.01}$ | $3.33_{2.53}$ | $.94_{.03}$ | $.06_{.05}$ | $3.40_{2.62}$ |
| | | .10 | $.91_{.02}$ | $.06_{.02}$ | $2.95_{2.25}$ | $.89_{.04}$ | $.10_{.07}$ | $3.01_{2.33}$ |
| | | .01 | $1.00_{.00}$ | $.01_{.00}$ | $7.75_{.83}$ | $.98_{.02}$ | $.01_{.00}$ | $7.75_{.83}$ |
| | APS | .05 | $1.00_{.00}$ | $.05_{.00}$ | $7.75_{.83}$ | $.98_{.02}$ | $.05_{.00}$ | $7.75_{.83}$ |
| | | .10 | $1.00_{.00}$ | $.10_{.00}$ | $7.75_{.83}$ | $.98_{.02}$ | $.10_{.00}$ | $7.75_{.83}$ |
| cluster | | .01 | $1.00_{.00}$ | $.01_{.00}$ | $7.75_{.83}$ | $.98_{.02}$ | $.01_{.00}$ | $7.75_{.83}$ |
| | RAPS | .05 | $1.00_{.00}$ | $.05_{.00}$ | $7.75_{.83}$ | $.98_{.02}$ | $.05_{.00}$ | $7.75_{.83}$ |
| | | .10 | $1.00_{.00}$ | $.10_{.00}$ | $7.75_{.83}$ | $.98_{.02}$ | $.10_{.00}$ | $7.75_{.83}$ |
| | | .01 | $1.00_{.00}$ | $.01_{.00}$ | $7.75_{.83}$ | $.98_{.02}$ | $.01_{.00}$ | $7.75_{.83}$ |
| | THR | .05 | $1.00_{.00}$ | $.05_{.00}$ | $7.75_{.83}$ | $.98_{.02}$ | $.05_{.00}$ | $7.75_{.83}$ |
| | | .10 | $1.00_{.00}$ | $.10_{.00}$ | $7.75_{.83}$ | $.98_{.02}$ | $.10_{.00}$ | $7.75_{.83}$ |

Table S16: Averaged performance scores for **Pancreas** tissue using TorchNet. Test data represent a small, unseen subset of the reference data (10%) containing no OOD samples and free from batch effects (*i.e.*, similar to validation sets in traditional ML pipelines). This set serves as a baseline to compare the performance of the annotator in the query data, which includes both OOD samples and batch effects, against ideal conditions.

# References


[1] Vladimir Vovk, Alexander Gammerman, and Glenn Shafe. *Algorithmic Learning in a Random World*. Springer-Cham, second edition, 2022.

[2] Anastasios Nikolas Angelopoulos and Stephen Bates. Conformal prediction: A gentle introduction. *Found. Trends in Mach. Learn.*, 16(4):494–591, 2023.

[3] Alexander Gammerman, Vladimir Vovk, and Vladimir Vapnik. Learning by transduction. In *Proceedings of the Fourteenth Conference on Uncertainty in Artificial Intelligence*, UAI'98, page 148–155, San Francisco, CA, USA, 1998. Morgan Kaufmann Publishers Inc.

[4] Harris Papadopoulos, Kostas Proedrou, Volodya Vovk, and Alexander Gammerman. Inductive confidence machines for regression. In *Proceedings of the 13th European Conference on Machine Learning*, ECML '02, page 345–356, Berlin, Heidelberg, 2002. Springer-Verlag.

[5] Yaniv Romano, Matteo Sesia, and Emmanuel Candes. Classification with valid and adaptive coverage. *Advances in Neural Information Processing Systems*, 33:3581–3591, 2020.

[6] Anastasios Nikolas Angelopoulos, Stephen Bates, Michael Jordan, and Jitendra Malik. Uncertainty sets for image classifiers using conformal prediction. In *International Conference on Learning Representations*, 2021.

[7] Lauren Theunissen, Thomas Mortier, Yvan Saeys, and Willem Waegeman. Uncertainty-aware single-cell annotation with a hierarchical reject option. *Bioinform.*, 40(3):btae128, 2024.

[8] Lauren Theunissen, Thomas Mortier, Yvan Saeys, and Willem Waegeman. Evaluation of out-of-distribution detection methods for data shifts in single-cell transcriptomics. *Brief. Bioinform.*, 26(3):bbaf239, 2025.

[9] Rikard Laxhammar and Göran Falkman. Conformal prediction for distribution-independent anomaly detection in streaming vessel data. In *Proceedings of the First International Workshop on Novel Data Stream Pattern Mining Techniques*, StreamKDD '10, page 47–55, New York, NY, USA, 2010. Association for Computing Machinery.

[10] Stephen Bates, Emmanuel Candès, Lihua Lei, Yaniv Romano, and Matteo Sesia. Testing for outliers with conformal p-values. *Ann. Stat.*, 51(1):149 – 178, 2023.

[11] Marco A.F. Pimentel, David A. Clifton, Lei Clifton, and Lionel Tarassenko. A review of novelty detection. *Signal Process.*, 99:215–249, 2014.

[12] Lukas Ruff, Robert Vandermeulen, Nico Goernitz, Lucas Deecke, Shoaib Ahmed Siddiqui, Alexander Binder, Emmanuel Müller, and Marius Kloft. Deep one-class classification. In Jennifer Dy and Andreas Krause, editors, *Proceedings of the 35th International Conference on Machine Learning*, volume 80 of *Proceedings of Machine Learning Research*, pages 4393–4402. PMLR, 10–15 Jul 2018.

[13] Bo Zong, Qi Song, Martin Renqiang Min, Wei Cheng, Cristian Lumezanu, Daeki Cho, and Haifeng Chen. Deep autoencoding gaussian mixture model for unsupervised anomaly detection. In *International Conference on Learning Representations*, 2018.

[14] Songqiao Han, Xiyang Hu, Hailiang Huang, Minqi Jiang, and Yue Zhao. Adbench: Anomaly detection benchmark. In S. Koyejo, S. Mohamed, A. Agarwal, D. Belgrave, K. Cho, and A. Oh, editors, *Advances in Neural Information Processing Systems*, volume 35, pages 32142–32159. Curran Associates, Inc., 2022.

[15] Abhaya Abhaya and Bidyut Kr. Patra. An efficient method for autoencoder based outlier detection. *Expert Syst Appl.*, 213:118904, 2023.

[16] Pascal Vincent, Hugo Larochelle, Isabelle Lajoie, Yoshua Bengio, and Pierre-Antoine Manzagol. Stacked denoising autoencoders: Learning useful representations in a deep network with a local denoising criterion. *J. Mach. Learn. Res.*, 11(110):3371–3408, 2010.

[17] Salah Rifai, Pascal Vincent, Xavier Muller, Xavier Glorot, and Yoshua Bengio. Contractive auto-encoders: explicit invariance during feature extraction. In *Proceedings of the 28th International Conference on International Conference on Machine Learning*, ICML'11, page 833–840, Madison, WI, USA, 2011. Omnipress.

[18] Vladimir Yu Kiselev, Andrew Yiu, and Martin Hemberg. scmap: projection of single-cell rna-seq data across data sets. *Nat. methods*, 15(5):359–362, 2018.

[19] Jianguo Huang, Jianqing Song, Xuanning Zhou, Bingyi Jing, and Hongxin Wei. Torchcp: A python library for conformal prediction, 2024.

[20] Cecilia Domínguez Conde, Chuan Xu, Lorna Jarvis, and Daniel Rainbow *et al.* Cross-tissue immune cell analysis reveals tissue-specific features in humans. *Sci.*, 376(6594):eabl5197, 2022.

[21] Samuel L. Wolock, Romain Lopez, and Allon M. Klein. Scrublet: Computational identification of cell doublets in single-cell transcriptomic data. *Cell Syst.*, 8(4):281–291.e9, 2019.

[22] Krzysztof Polański, Matthew D Young, Zhichao Miao, Kerstin B Meyer, Sarah A Teichmann, and Jong-Eun Park. Bbknn: Fast batch alignment of single cell transcriptomes. *Bioinform.*, 36(3):964–965, 2019.

[23] Ilya Korsunsky, Nghia Millard, Jean Fan, Kamil Slowikowski, Fan Zhang, Kevin Wei, Yuriy Baglaenko, Michael Brenner, Po-ru Loh, and Soumya Raychaudhuri. Fast, sensitive and accurate integration of single-cell data with harmony. *Nat. methods*, 16(12):1289–1296, 2019.

[24] F Alexander Wolf, Philipp Angerer, and Fabian J Theis. Scanpy: large-scale single-cell gene expression data analysis. *Genome Biol.*, 19:1–5, 2018.

[25] Tiffany Ding, Anastasios Angelopoulos, Stephen Bates, Michael Jordan, and Ryan J Tibshirani. Class-conditional conformal prediction with many classes. In A. Oh, T. Naumann, A. Globerson, K. Saenko, M. Hardt, and S. Levine, editors, *Advances in Neural Information Processing Systems*, volume 36, pages 64555–64576. Curran Associates, Inc., 2023.
